# Multi-omics analysis of *Taiwanofungus gaoligongensis*: effects of different cultivation methods on secondary metabolites

**DOI:** 10.3389/fmicb.2025.1620693

**Published:** 2025-08-01

**Authors:** Tingwen He, Xiaolong Yuan, Liangjun Xiao, Tanggeran Hu, Yi Wang, Xiaolei Zhao, Lu Li, Chengbo Peng, Hongling Zhang, Yuan Zheng

**Affiliations:** ^1^College of Forestry, Southwest Forestry University, Kunming, China; ^2^College of Biological and Food Engineering, Southwest Forestry University, Kunming, China; ^3^Yunnan Key Laboratory of Biodiversity of Gaoligong Mountain, Yunnan Academy of Forestry and Grass-Land, Kunming, China; ^4^Key Laboratory of State Forestry Administration on Highly-Efficient Utilization of Forestry Biomass Resources in Southwest China, Southwest Forestry University, Kunming, China; ^5^Edible/Medicinal Fungi Research Innovation Team, Modern Industry School of Edible-fungi, Southwest Forestry University, Kunming, China; ^6^Forest Resources Exploitation and Utilization Engineering Research Center for Grand Health of Yunnan Provincial Universities, Southwest Forestry University, Kunming, China

**Keywords:** *Taiwanofungus gaoligongensis*, reference-based transcriptomic analysis, untargeted metabolomic analysis, secondary metabolites, transcriptional regulation, transcription factor

## Abstract

A multi-omics strategy was utilized in this study to investigate the effects of various cultivation methods—including the fruiting bodies cultivation on *Cinnamomum kanehirae* wood logs (GLG), the mycelia cultivation on *C. kanehirae* substrate fungal cultivation bags (NZJB), *Cinnamomum camphora* substrate fungal cultivation bags (XZJB) and rice medium (DM)—on Secondary Metabolites in *Taiwanofungus gaoligongensis*. NZJB and XZJB significantly enhanced terpenoids production in the mycelium, with triterpenoid contents in NZJB and XZJB being sevenfold and 3.9-fold higher, respectively, than those in DM. Antcins were notably increased in fungal cultivation bag cultures: antcin C reached the highest level in XZJB (9.72-fold higher than in DM), antcin I peaked in NZJB (12.83-fold higher than in DM), and antrodin C also reached its maximum in NZJB. Additionally, the antrodin C content in NZJB was 3.2-fold higher than in *GLG* and 4.08-fold higher than in DM. In addition, the levels of steroids, phenolic compounds, and flavonoids were also significantly increased in NZJB and XZJB. Transcriptome analysis revealed significant differences in the expression of genes involved in the biosynthesis of antcins and antrodin C across the different cultivation methods. In particular, the expression of *TgHMGR* was markedly higher in NZJB than in XZJB and DM, correlating with the elevated terpenoids and triterpenoids levels, suggesting that TgHMGR may act as a key rate-limiting enzyme in the terpenoid biosynthesis pathway of *T. gaoligongensis*. The expression levels of terpenoid biosynthesis-related genes were significantly elevated in GLG compared to mycelium, consistent with the higher abundance of terpenoid metabolites. Co-expression analysis of transcription factors (TFs) and promoter binding site predictions indicated that the expression of *TgHMGR* and *TgFPPS 2* may be regulated by *TgHSF4* and *TgMYB6*, respectively. Meanwhile, the expression of *TgErg2*, *TgErg3*, *TgErg5*, and *TgErg6 1* may be regulated by *TgZnF1*, *TgMYB9*, *TgHOX1*, and *TgHMG8*. This study compared the metabolite profiles and gene expression patterns of the fruiting bodies of *T. gaoligongensis* with those of three types of cultivated mycelia. The results provide new insights into the transcriptional regulation of key bioactive compound biosynthesis in *T. gaoligongensis* and suggest potential strategies to enhance the production of active compounds in mycelia through artificial cultivation, thereby improving its medicinal value and production efficiency.

## Introduction

*Taiwanofungus camphoratus* is taxonomically classified within the phylum Basidiomycota, the family Polyporaceae, and the genus *Taiwanofungus*. It contains a variety of bioactive compounds, including polysaccharides, triterpenoids, ubiquinone derivatives, maleic and succinic acid derivatives, benzene derivatives, and glycoproteins, among which triterpenoids exhibit particularly notable antitumor activity (Ganesan et al., 2019; Li et al., 2022; Yeh et al., 2009). The major triterpenoids in *T. camphoratus* include lanostane-type and ergostane-type triterpenoids. Ergostane-type triterpenoids are among the most distinctive constituents of *T. camphoratus*. To date, 75 such compounds have been isolated and characterized, with most possessing Δ^8^ double bonds, and a few containing Δ^7,9(11)^ double bonds (Kuang et al., 2021). Antcins, a unique subgroup of ergostane-type triterpenoids with an ergostane skeleton from *T. camphoratus*, have demonstrated diverse biological activities, including anticancer, anti-inflammatory, antioxidant, antidiabetic, anti-aging, immunomodulatory, hepatoprotective, and hypolipidemic effects (Senthil Kumar et al., 2020; Achudhan et al., 2021; Gokila Vani et al., 2013; Huo et al., 2017; Wang Y. et al., 2019; Chen et al., 2011). Antcins are more abundant in the fruiting bodies of *T. camphoratus*, but their content is significantly reduced or absent in artificially cultured samples (Senthil Kumar et al., 2020).

*Taiwanofungus camphoratus* naturally occurs exclusively on the endangered host plant *C. kanehirae* in the wild, and its fruiting body develops extremely slowly, resulting in severely limited natural resources. Artificial cultivation of *T. camphoratus* fruiting bodies on *C. kanehirae* substrates yields a higher diversity of metabolites, particularly ergostane-type triterpenoids (Lin et al., 2011). In contrast, although mycelial cultures require only a few weeks for cultivation, the variety and content of bioactive compounds are significantly lower than those in the fruiting body (Lu et al., 2013). Different cultivation methods and conditions can lead to significant variations in the composition of active compounds. Moreover, certain additives have markedly enhanced triterpenoid production (Zhang et al., 2019; Qiao et al., 2015). For example, the addition of 1% (v/v) corn oil to the liquid fermentation medium increased triterpenoid production in *T. camphoratus* by fourfold compared to the control (Meng et al., 2021). Linolenic acid supplementation to the liquid fermentation medium enhanced cell membrane permeability, improved cellular metabolic activity, and promoted the biosynthesis of triterpenoid secondary metabolites (Tang et al., 2024). A previous study also reported that the addition of methanolic extracts from the trunks of *C. kanehirae* during deep fermentation effectively promoted terpenoid production in *T. camphoratus*. Specifically, monoterpenes such as linalool and alpha-pinene in the extracts upregulated the expression of key enzymes in the mevalonate (MVA) pathway (Luo et al., 2023). Furthermore, petroleum ether extracts from *C. kanehirae* rhizomes and their main component, Alpha-terpineol, significantly enhanced triterpenoid content and biosynthesis in deep fermentation (Lu et al., 2014). Additionally, the inclusion of ethanol extracts from *C. kanehirae* leaves in solid-state fermentation promoted both the growth of *T. camphoratus* and the production of active metabolites (Zeng et al., 2021).

Antrodin C is a triquinane-type sesquiterpene exhibiting broad-spectrum anticancer activity, as well as notable inhibitory effects against hepatitis C virus and liver fibrosis (Hsieh et al., 2023; Phuong et al., 2009; Kumar et al., 2015; Wang Y. et al., 2019; Xu et al., 2022). Previous studies have demonstrated that antrodin C production in *T. camphoratus* can be significantly enhanced through various artificial cultivation strategies. For instance, liquid fermentation with pH adjustment and glucose supplementation during incubation was shown to increase antrodin C yields (Zhang et al., 2014), Additionally, supplementation with inositol (Jia et al., 2023), *in situ* extractive fermentation using oleic acid as an extractant combined with coenzyme Q_0_ addition (Liu et al., 2020; Liu et al., 2019), and particle-enhanced fermentation employing talc as a carrier (Fan et al., 2023), have all been reported to improve antrodin C production. Furthermore, optimization of inorganic salt composition and cultivation methods in solid-state fermentation using soybean meal as a substrate has also yielded favorable outcomes for antrodin C synthesis (Xia et al., 2014).

The precursor for triterpenoid biosynthesis, 2,3-oxidosqualene, is synthesized from acetyl coenzyme A via the mevalonate (MVA) pathway (Adiguzel et al., 2016), Subsequently, 2,3-oxidosqualene is cyclized into lanosterol and other cyclic triterpenoid products by 2,3-oxidosqualene cyclase (Lin et al., 2015), Additional structural diversification of triterpenoids is mediated by scaffold-modifying enzymes such as cytochrome P450 monooxygenases (P450s), UDP-glycosyltransferases (UGTs), and acyltransferases (ATs; Dinday and Ghosh, 2023; Lee et al., 2010). Functional characterization of three key post-modification enzymes involved in the biosynthesis of lanostane-type triterpenoids in *T. camphoratus* has been reported. Among these, AcSDR6 catalyzes the conversion of antcamphorol K to antcin C through dehydrogenation at the C-3 position (Zhang et al., 2024b). Ergosterol analogs represent a group of natural products derived from lanosterol via dehydrogenation at the C(14) and C(4) positions and methylation at the C(24) position.

The biosynthesis of ergostane-type triterpenoids, such as antcins, may be associated with the ergosterol biosynthetic pathway in fungi. Ergosterol is a vital component of fungal cell membranes and serves as an essential precursor for the production of various bioactive steroidal secondary metabolites. It plays a critical role in fungal growth, development, and adaptation to environmental stresses. Substantial progress has been made in elucidating the ergosterol biosynthesis pathway in *Saccharomyces cerevisiae*. In this organism, ergosterol is synthesized from lanosterol through a series of enzymatic reactions catalyzed by several ERG genes, including *ERG11, ERG24, ERG25, ERG26, ERG27, ERG6, ERG2, ERG3, ERG4*, and *ERG5* (Hu et al., 2017). Lanosterol functions as a key intermediate in this biosynthetic route. Previous studies have suggested that genes involved in the terpene backbone synthesis pathway—*IDI, E2.3.3.10, HMGCR*, and *atoB*—annotated in this study as *TgAACT, TgHMGS, TgHMGR*, and *TgIDI*, along with genes from the ubiquinone and other terpene quinone synthesis pathways—*COQ2, ARO8*, and *wrbA*—play important roles in antrodin C biosynthesis (Liu et al., 2020; Jia et al., 2023).

*Taiwanofungus gaoligongensis* Chen and Yang is a newly identified species of the genus *Taiwanofungus*, discovered in the Gaoligong Mountains of Baoshan, Yunnan, in 2018. Its growth characteristics and gene sequence show the closest similarity to *T. camphoratus* within the same genus (Zhang et al., 2024a; Yin et al., 2024). In this study, we systematically investigated the biosynthetic mechanisms of antcins and antrodin C in *T. gaoligongensis* using multi-omics analysis. The associations between these triterpenoids and the corresponding biosynthetic pathway genes were examined by analyzing differences in metabolite profiles and gene expression under various cultivation methods, and putative transcription factors (TFs) involved in their regulation were predicted. The results demonstrated that culturing in *C. kanehirae* and *C. camphora* fungal cultivation bags significantly promoted the accumulation of triterpenoid metabolites, providing a theoretical basis for further elucidation of the biosynthetic mechanisms of active components in *T. gaoligongensis*.

## Materials and methods

### Microbial strains

*Taiwanofungus gaoligongensis* strain YAF008 was deposited in the Yunnan Key Laboratory of Bio-diversity of Gaoligong Mountain, Yunnan Academy of Forestry and Grassland Sciences in Kunming, and the China Center for Type Culture Collection (deposit number: CCTCC M 20232425).

### Cultivation methods

Isolated strains of *T. gaoligongensis* were first inoculated onto potato dextrose agar (PDA) slants and incubated at 28 °C for 15 days, followed by storage at 4 °C. Mycelia were transferred from the slants into 500 mL Erlenmeyer flasks containing 100 mL of seed medium (20 g/L dextrose, 5 g/L yeast extract, 1 g/L KH_2_PO_4_, 0.5 g/L MgSO_4_, and 0.1 g/L vitamin B_1_), and incubated at 28 °C for 15 days with shaking at 150 rpm. After incubation, the culture was filtered through four layers of sterile gauze to obtain the spore suspension, which was then inoculated into different media and cultured at 28 °C for 30 days.

The sample medium formulation used for metabolomic and transcriptomic analyses was designated as GLG, consisting of *T. gaoligongensis* fruiting bodies cultured on *C. kanehirae* wood. NZJB medium was prepared by mixing 200 g of *C. kanehirae* sawdust, 180 g of rice, and 400 mL of MM medium in fungal cultivation bags and incubating for 30 days. XZJB medium consisted of 200 g of *C. camphora* sawdust, 180 g of rice, and 400 mL of MM medium, similarly incubated for 30 days. For the DM medium, 50 g of rice and 50 mL of MM medium were mixed in culture flasks and incubated for 30 days. The MM medium contained 6 g/L sodium nitrate, 0.52 g/L potassium chloride, 1.52 g/L potassium dihydrogen phosphate, and 0.52 g/L magnesium sulfate. Fungal cultivation bags were made of polyethylene (16.5 cm × 37 cm) and had a capacity of 1200 mL. *C. kanehirae* and *C. camphora* sawdust were pre-sterilized by autoclaving at 121°C for 120 min. After the fungal cultivation bags were prepared, they were further sterilized by autoclaving at 121°C for 120 min, and this process was repeated twice. The rice-based medium was sterilized by autoclaving at 121°C for 20 min. After cultivation, the mycelium was separated from the substrate using forceps and transferred into 2 mL centrifuge tubes, rapidly frozen in liquid nitrogen for 5 min, and then stored at −80°C. Three biological replicates of each sample were used for metabolomics analysis, and a pooled transcript sample was used for transcriptome analysis.

### Untargeted metabolomics analysis

Metabolite Extraction: Weigh 60 mg of the sample into a 2 mL centrifuge tube. Add 500 μL of pre-chilled methanol (−20°C) and 500 μL of cold water (4°C), then add 100 mg of glass beads and vortex for 30 s. Place the centrifuge tube into a 2 mL adapter, immerse it in liquid nitrogen for 5 min, then remove and allow it to thaw at room temperature. Mount the centrifuge tube in a grinder using a 2 mL adapter and oscillate at 55 Hz for 2 min, performing two grinding cycles. Centrifuge the tube at 12,000 rpm for 10 min at 4°C. The supernatant is collected, concentrated, and dried by centrifugation. Reconstitute the dried sample in 300 μL of 50% aqueous methanol solution (1:1, 4°C) containing 2-chlorophenylalanine (4 ppm). Filter through a 0.22 μm membrane to obtain the final sample for analysis. The prepared sample is then subjected to LC-MS analysis.

Chromatographic Conditions: An ACQUITY UPLC^®^ HSS T3 column (1.8 μm, 2.1 × 150 mm) was employed. The autosampler temperature was maintained at 8°C. A 2 μL aliquot of the sample was injected at a flow rate of 0.25 mL/min, with the column temperature set to 40°C. Gradient elution was performed using the following mobile phases: for positive ion mode, 0.1% formic acid in water (C) −0.1% formic acid in acetonitrile (D); for negative ion mode, 5 mM ammonium formate in water (A) - acetonitrile (B). The gradient program was as follows: 0∼1 min, 2% B/D; 1∼9 min, 2%∼50% B/D; 9∼12 min, 50%∼98% B/D; 12∼13.5 min, 98% B/D; 13.5∼14 min, 98%–2% B/D; 14∼20 min, 2% D (positive mode) or 14∼17 min, 2% B (negative mode).

Mass Spectrometry Conditions: The instrument was operated using an electrospray ionization (ESI) source in both positive and negative ion modes. The spray voltage was set to 3.50 kV for positive mode and 2.50 kV for negative mode. The sheath gas and auxiliary gas were set at 30 and 10 arbitrary units (arb), respectively. The capillary temperature was maintained at 325°C. Full-scan acquisition was performed at a resolution of 70,000 over an m/z range of 81–1,000. Fragmentation was conducted using higher-energy collisional dissociation (HCD) with a collision energy of 30 eV. Dynamic exclusion was applied to eliminate redundant MS/MS data.

Data Processing and Statistical Analysis: The raw data were converted to mzXML format using ProteoWizard software (v3.0.8789). Peak detection, filtering, and alignment were performed using the XCMS package in R (v3.3.2), resulting in a data matrix comprising the mass-to-charge ratio (m/z), retention time (rt), and peak intensity. After data processing, metabolite identification was conducted by querying several databases, including the Human Metabolome Database (HMDB, METLIN (see text foot note 1)^1^, MassBank^2^, LipidMaps^3^, and mzCloud^4^. Multivariate statistical analyses, including Principal component analysis (PCA) and partial least squares discriminant analysis (PLS-DA), were performed to visualize metabolic differences between experimental groups. Metabolites with significant variation were screened based on variable importance in projection (VIP > 1) and *p*-value (*P* < 0.05). The metabolite content of mycelia cultured on *T. gaoligongensis* fruiting bodies, rice medium, and fungal cultivation bags containing *C. kanehirae* and *C. camphora* substrates was analyzed using one-way ANOVA followed and Tukey’s *post-hoc* test. Each treatment included three biological replicates.

### Transcriptome analysis

The genome of *T. gaoligongensis* (GenBank accession number: JAZIAZ000000000)^5^ was used as the reference genome (Bao et al., 2024). Four samples (GLG, NZJB, XZJB, and DM) were sequenced using paired-end sequencing on the Illumina HiSeq platform using NGS technology. The resulting short reads were aligned to the reference genome, enabling quantitative analysis of gene expression and functional annotation. We quantified gene expression levels in the transcriptome data as fragments per kilobase of transcript per million mapped reads (FPKM) values. TBtools software (v2.142) was used to generate interactive heatmaps to visualize the expression patterns of the target genes.

### Cluster and conserved motif analysis of protein sequences

Known protein sequences of the target genes were retrieved from the NCBI database and aligned with the protein sequences obtained in this study using the ClustalW algorithm in MEGA11 software. Phylogenetic trees were constructed using the maximum likelihood method, with 1,000 bootstrap replications performed under default parameters (Tamura et al., 2021). Additionally, protein sequence similarity was assessed using the Protein BLAST tool (accessed July 17, 2024)^6^. Conserved motifs within the protein sequences were predicted using the MEME Suite^7^.

### Prediction of transcription factor binding sites

Based on the whole-genome and transcriptome data of *T. gaoligongensis*, the 2,000 bp upstream regions of DNA sequences of PKS, TPS, and target genes with expression patterns similar to *tf* were extracted using TBtools software (v2.142). Putative TF binding sites in the promoter regions of co-expressed genes were subsequently predicted using the JASPAR online tool with a confidence threshold of 90% (Rauluseviciute et al., 2024).

### Quantitative real-time PCR analysis

Selection of key terpene biosynthesis genes in *T. gaoligongensis*, along with TFs that may regulate these genes—including *TgHMGR*, *TgHSF4*, *TgErg6 1*, *TgHMG8*, *TgHMGS*, *TgFPPS 1*, *TgSQS*, *TgOSC*, *TgErg11*, *TgErg25*, *TgErg26*, and *TgErg6 2*—was conducted. Primers for these genes were designed using Primer Premier 5.0 software to evaluate their expression levels (Supplementary Table 8). The PCR reaction mixture consisted of 20 μL total volume, comprising 10 μL of PCR mix, 1 μL of DNA/cDNA template, 2 μL of primers, and 7 μL of deionized water. The PCR conditions included an initial denaturation at 94°C for 2 min, followed by 40 amplification cycles (94°C for 15 s, 65°C for 15 s, 72°C for 45 s), and a final extension at 72°C for 10 min. Each treatment included three biological replicates.

## Results

### Untargeted metabolomics analysis

**FIGURE 1:**
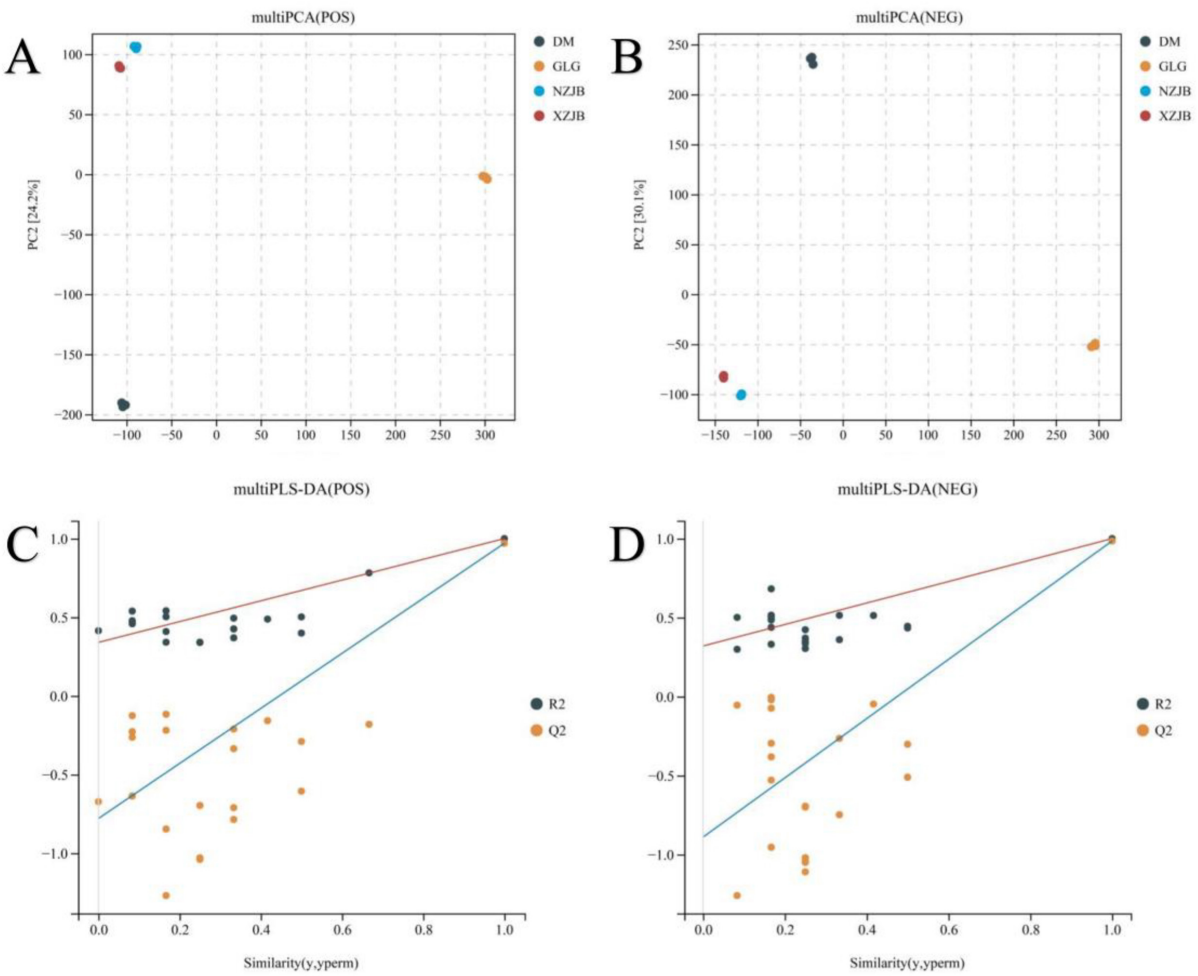
Multivariate statistical analysis of metabolome samples. **(A)** Metabolome samples PCA (POS); **(B)** metabolome samples PCA (NEG); **(C)** metabolome samples PLS-DA (POS) permutation test plot; **(D)** metabolome samples PLS-DA (NEG) permutation test plot. PC1 represents principal component 1 and PC2 represents principal component 2, each point represents one sample, and points of different colors indicate different subgroups. The PLS-DA permutation test plot is reliable and valid when any of the following points are met: (1) all Q2 points are lower than the original Q2 point on the far right (it is possible that the Q2 point on the far right of the plot coincides with the R2 point on the top right corner); (2) the intersection of the regression line of the Q2 point and the vertical coordinate is less than 0.

During the cultivation of *T. gaoligongensis*, it was observed that the growth performance in fungal cultivation bags was superior to that in culture bottles using rice as the substrate. Moreover, when *T. gaoligongensis* was cultivated in fungal cultivation bags with *C. kanehirae* or *C. camphora* as the substrate, no significant difference in growth was observed between the two. The effects of *C. kanehirae* and *C. camphora* substrates on the cultivation of *T. gaoligongensis* in fungal cultivation bags were further investigated by analyzing *T. gaoligongensis* fruiting bodies and mycelia under different cultivation methods using untargeted metabolomics. PCA and PLS-DA revealed substantial differences between groups, indicating that the cultivation condition significantly influenced the fungal cultivation of *T. gaoligongensis* (Figure 1).

**FIGURE 2:**
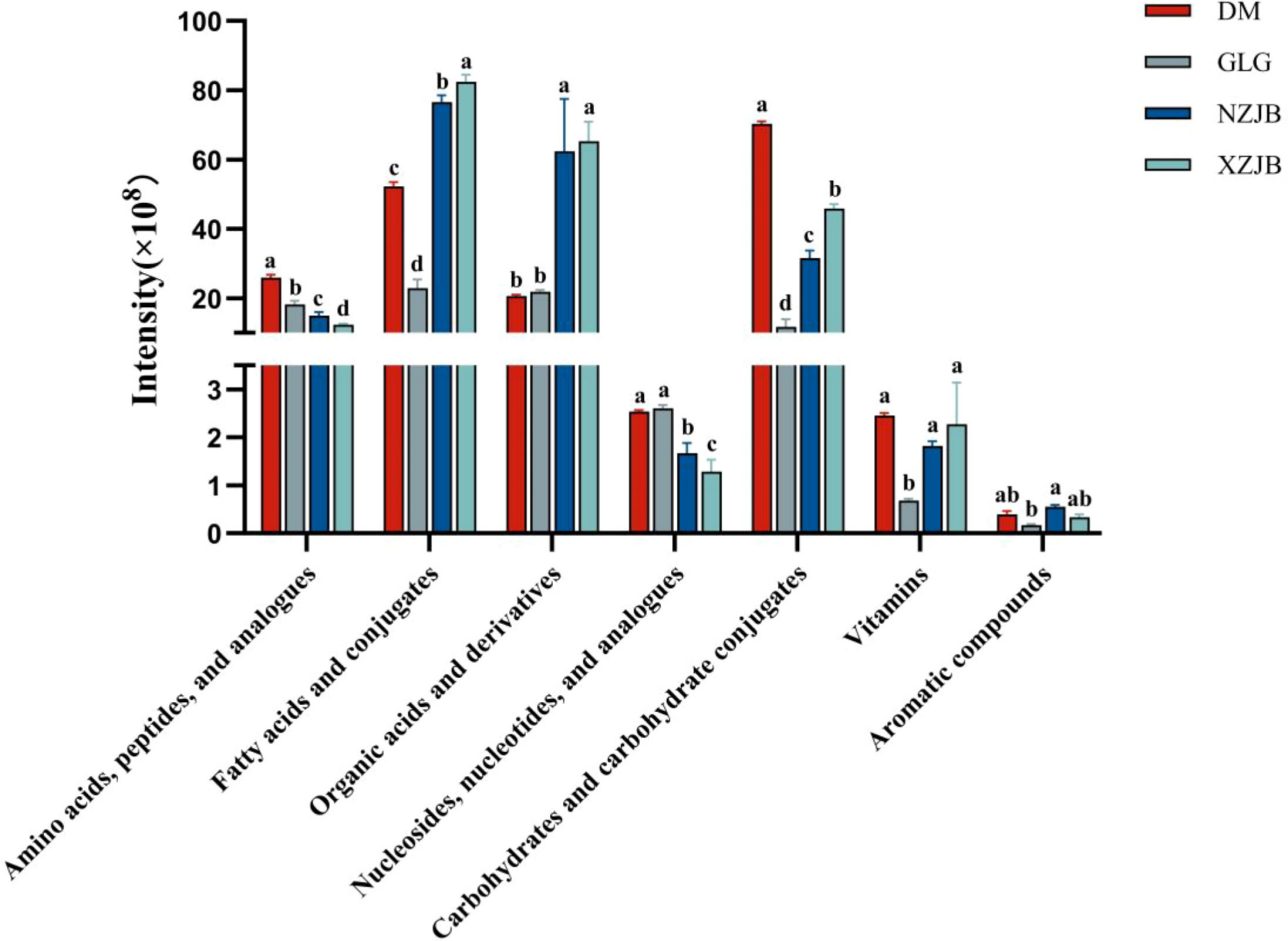
Variation in primary metabolites content among different *T. gaoligongensis* samples. Mean ± SD (*n* = 3) was used, According to Tukey’s multiple range test, samples from different treatments labeled with the same letter are not significantly different at the *p* < 0.05 significance level. DM, mycelia cultured on rice medium; GLG, *T. gaoligongensis* fruiting bodies; NZJB, mycelia cultured in fungal cultivation bags containing *C. kanehirae* substrate; XZJB, mycelia cultured in fungal cultivation bags containing *C. camphora* substrate.

We classified the identified metabolites into primary and secondary metabolites. Primary metabolites included seven classes: amino acids, fatty acids, and organic acids, etc. whereas secondary metabolites consisted of nine classes, including steroids, terpenoids, phenols, flavonoids, etc. A comparison of the total abundance of each metabolite type across the four treatment groups revealed that the levels of organic acids and their derivatives among primary metabolites were significantly elevated in the NZJB and XZJB groups, approximately three times higher than those in the DM and GLG groups. Fatty acids and their derivatives, as well as carbohydrates and carbohydrate conjugates, were significantly increased in the mycelium, with fatty acid levels 2.3, 3.3, and 3.6 times higher in the DM, NZJB, and XZJB groups, respectively, compared to GLG. Similarly, the levels of carbohydrates and carbohydrate conjugates were 6, 2.7, and 3.9 times higher in DM, NZJB, and XZJB, respectively, than in GLG (Figure 2).

**FIGURE 3:**
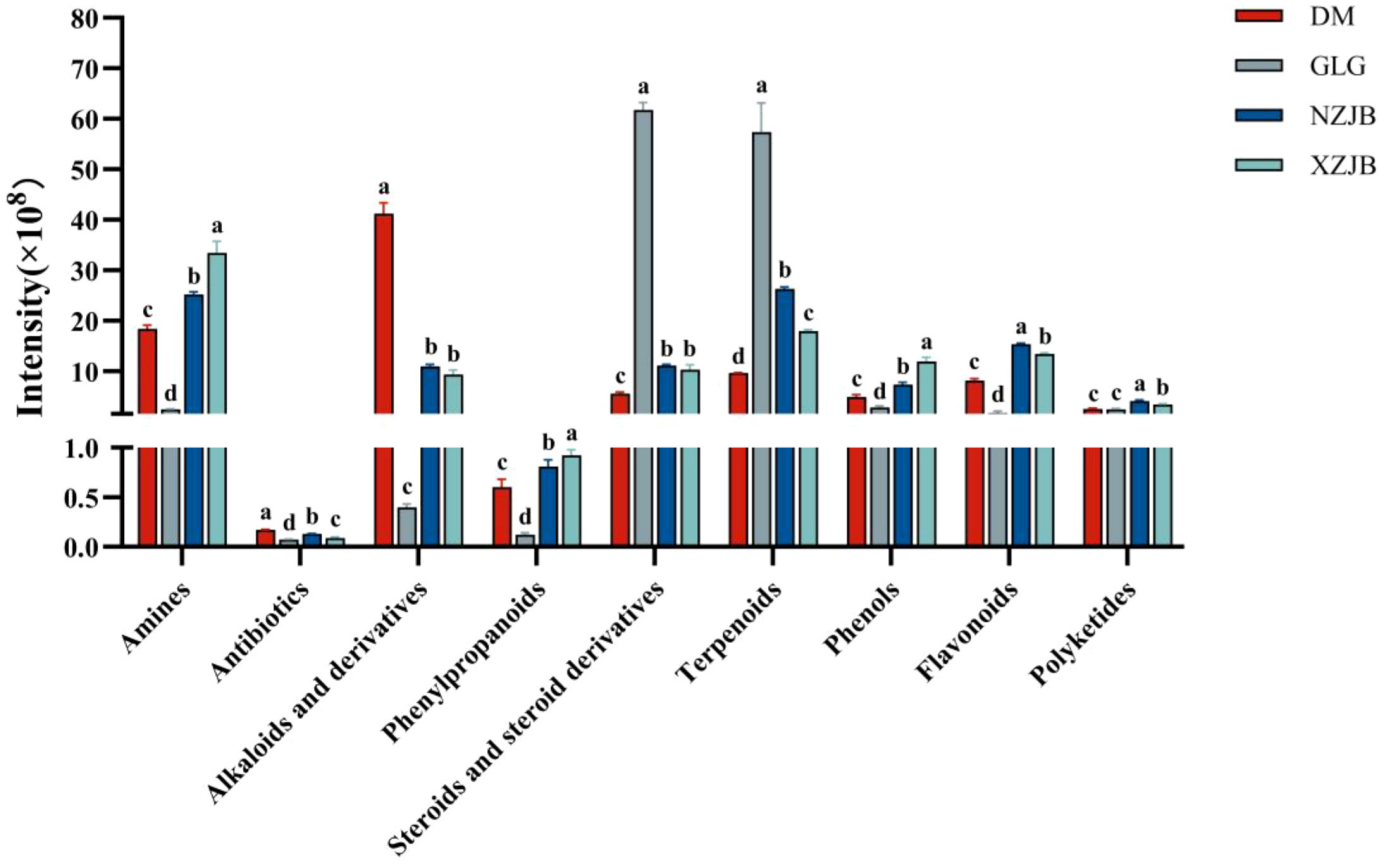
Variation in secondary metabolites content among different *T. gaoligongensis* samples. Mean ± SD (*n* = 3) was used, According to Tukey’s multiple range test, samples from different treatments labeled with the same letter are not significantly different at the *p* < 0.05 significance level. DM, mycelia cultured on rice medium; GLG, *T. gaoligongensis* fruiting bodies; NZJB, mycelia cultured in fungal cultivation bags containing *C. kanehirae* substrate; XZJB, mycelia cultured in fungal cultivation bags containing *C. camphora* substrate.

Among the secondary metabolites, steroids and terpenoids exhibited the highest levels in GLG, followed by those in NZJB and XZJB. Terpenoids were 2.7 and 1.8 times more abundant in NZJB and XZJB, respectively, compared to DM, while steroids were 2 and 1.8 times more abundant in NZJB and XZJB, respectively, than in DM. The levels of steroid compounds in NZJB and XZJB were 2 and 1.8 times higher than in DM. Alkaloids and their derivatives were most abundant in DM, being approximately four times more abundant than in NZJB and XZJB. Amines, antibiotics, phenylpropanoids, phenolics, and flavonoids were significantly increased in the mycelium, whereas polyketides were significantly elevated in NZJB and XZJB (Figure 3).

**FIGURE 4:**
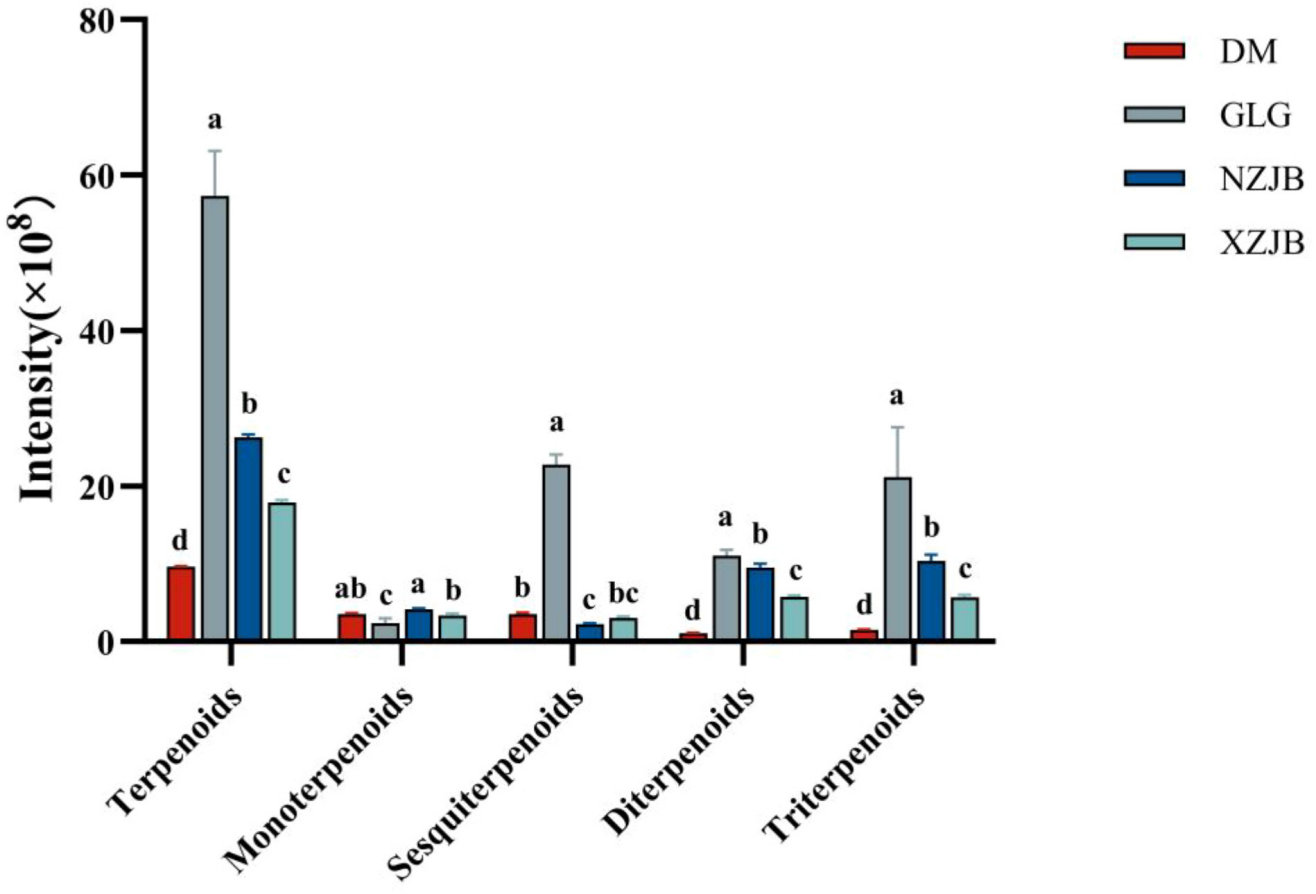
Variation in terpenoid compounds content among different *T. gaoligongensis* samples. Mean ± SD (*n* = 3) was used, According to Tukey’s multiple range test, samples from different treatments labeled with the same letter are not significantly different at the *p* < 0.05 significance level. DM, mycelia cultured on rice medium; GLG, *T. gaoligongensis* fruiting bodies; NZJB, mycelia cultured in fungal cultivation bags containing *C. kanehirae* substrate; XZJB, mycelia cultured in fungal cultivation bags containing *C. camphora* substrate.

The highest terpenoids content was observed in GLG, which was 5.9, 2.2, and 3.2 times higher than that of DM, NZJB, and XZJB, respectively. The compounds identified in the metabolome were categorized into monoterpenoids, sesquiterpenoids, diterpenoids, and triterpenoids. The contents of diterpenoids and triterpenoids in NZJB and XZJB were elevated compared to DM, with the diterpenoids content in NZJB being 9 times higher than in DM, and the triterpenoids content being 7 times higher. Similarly, the diterpenoids content in XZJB was 5.4 times higher than in DM, while the triterpenoids content was 3.9 times higher. The sesquiterpenoids content in GLG was significantly higher than in the other three culture-mode mycelia, ranging from 6 to 10 times higher. In addition, the content of monoterpenoids in NZJB and XZJB was significantly higher than that in GLG (Figure 4).

A total of seven antcins were identified in the metabolomic samples (Supplementary Table 1). The content of antcins in the fruiting bodies of *T. gaoligongensis* was significantly higher than that in the other three culture-mode mycelia. In addition, among the antcins, the content of antcin C was the highest in XZJB, being 9.72 times greater than in DM, and it accounted for 98% of the total antcins in XZJB. Antcin I was the most abundant in NZJB, with a concentration 12.83 times higher than in DM, contributing to 87% of the total antcins content in NZJB. The levels of antcin H and antcin K were lower and did not differ significantly across the mycelia under different cultivation methods. Furthermore, antcin B was found to be lower in all three samples, but its content in NZJB and XZJB was significantly higher compared to DM (Table 1).

**TABLE 1:** The content of antcins in different samples of *T. gaoligongensis*. The results are presented in terms of peak areas.

Antcins	DM	GLG	NZJB	XZJB
Antcin B	217923.57	35926761.55	365685.35	379826.61
Antcin C	13481140.83	833024393.86	7271973.34	131067209.13
Antcin H	449286.45	519909692.68	1109800.1	538755.65
Antcin I	4787334.79	99591540.49	61436850.32	1396800.25
Antcin K	120468.6	83662265.9	416101.12	392753.17

**FIGURE 5:**
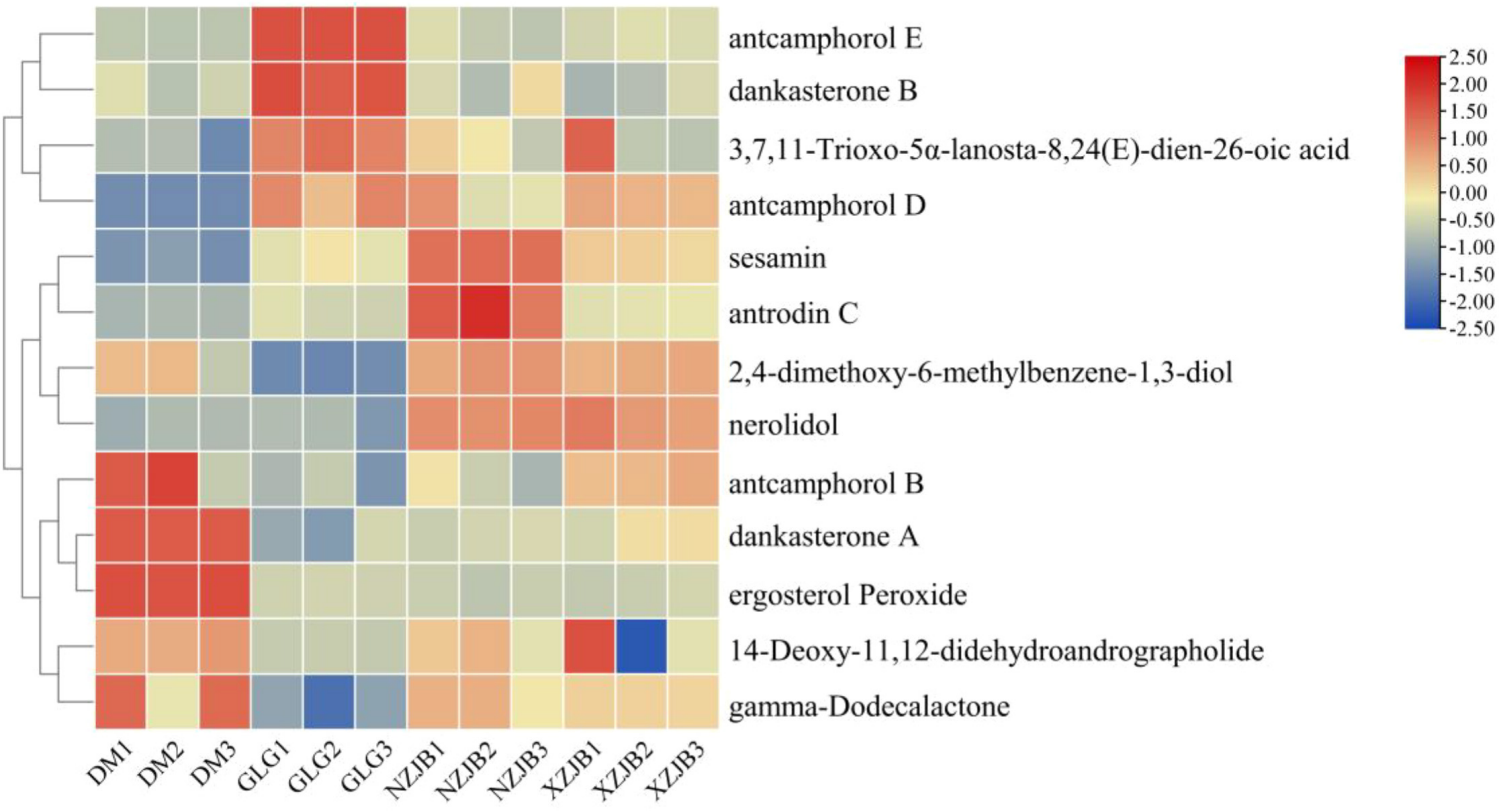
Heatmap the expression levels of *T. camphoratus* metabolites detected in the metabolomic samples, as reported in the literature. Heatmap was generated using TBtools software (version 2.142). DM, mycelia cultured on rice medium; GLG, *T. gaoligongensis* fruiting bodies; NZJB, mycelia cultured in fungal cultivation bags containing *C. kanehirae* substrate; XZJB, mycelia cultured in fungal cultivation bags containing *C. camphora* substrate.

In addition to the antcins, 13 *T. camphoratus* metabolites previously reported in the literature were detected in the metabolomic samples, including 3,7,11-trioxo-5α-lanosta-8,24(E)-dien-26-oic acid, antcamphorol B, antcamphorol D, antcamphorol E, dankasterone A, dankasterone B, 2,4-dimethoxy-6-methylbenzene-1,3-diol, 14-deoxy-11,12-didehydroandrographolide, sesamin, antrodin C, ergosterol peroxide, nerolidol, and gamma-dodecalactone. Of these, antcamphorol D, antcamphorol E, dankasterone B, and 3,7,11-trioxo-5α-lanosta-8,24(E)-dien-26-oic acid were more abundant in the fruiting bodies of *T. gaoligongensis* than in the mycelia. In contrast, nine compounds exhibited higher levels in the mycelia than in the fruiting bodies. For example, dankasterone A (a sesquiterpenoid) was 54 times more abundant in DM than in GLG, ergosterol peroxide (an ergostane steroid) was 5.8 times more abundant in DM than in GLG, nerolidol (a sesquiterpenoid) was 55 and 53 times more abundant in NZJB and XZJB, respectively, than in GLG, and sesamin (lignan) and antrodin C (triquinane-type sesquiterpenoid) were 11 times and 3.2 times higher, respectively, in NZJB compared to GLG (Figure 5).

### Transcriptomic analysis

Low-quality read segments, including chimeric sequences and bases with ambiguous or low-quality scores, were removed through bioinformatics analysis of the sequencing data. Table 2 presents the statistics of high-quality mapped read segments obtained from the RNA-Seq analysis.

**TABLE 2:** Summary of sequencing data quality and the statistics of the transcriptome assembly.

Sample	Clean reads	Q20 (%)	Q30 (%)	GC content (%)	Total mapped	Multiple mapped	Uniquely mapped
DM	45352460	98.84	96.54	53.73	44659543 (98.47%)	1192558 (2.67%)	43466985 (97.33%)
GLG	44720798	98.38	95.23	51.66	39290529 (87.86%)	1134667 (2.89%)	38155862 (97.11%)
NZJB	42392814	98.3	95.6	48.6	40258296 (94.96%)	8508682 (21.14%)	31749614 (78.86%)
XZJB	55424280	98.79	96.4	53.56	54672344 (98.64%)	2430380 (4.45%)	52241964 (95.55%)

Given the similar cultivation methods and metabolic states of NZJB and XZJB, and based on the statistics of mapped transcriptome read segments, XZJB, with higher transcriptome quality, was selected for further transcriptome analysis alongside the GLG and DM. The correlation heatmap results demonstrated that the gene expression profiles of the three samples were significantly distinct from one another, with a higher degree of similarity observed between the artificially cultured mycelia XZJB and DM compared to the fruiting bodies GLG (Supplementary Figure 2).

**FIGURE 6:**
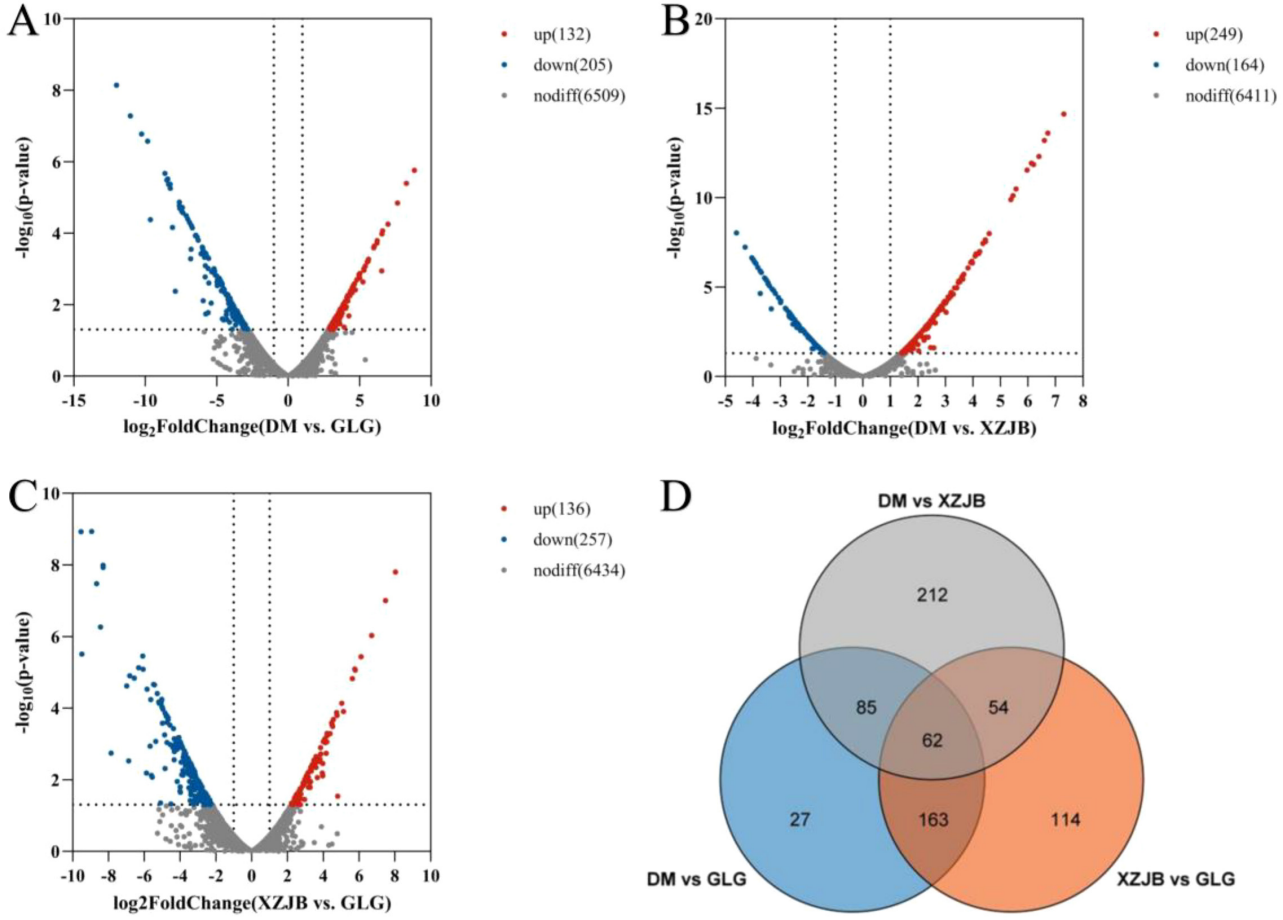
Volcano plot of differentially expressed genes (DEGs) in the comparisons: **(A)** XZJB vs. GLG, **(B)** DM vs. GLG, and **(C)** DM vs. XZJB. Significantly up-regulated or down-regulated genes are labeled with red dots or blue dots, respectively. **(D)** Venn diagram of DEGs.

Differentially expressed genes (DEGs) among the different samples were analyzed using volcano plots and Venn diagrams. As shown, DEGs varied between GLG and XZJB, GLG and DM, and XZJB and DM. Specifically, 393 DEGs were identified between GLG and XZJB, 337 between GLG and DM, and 413 between XZJB and DM. Among these, 114 DEGs were uniquely expressed between GLG and XZJB, 27 were unique to GLG and DM, and 212 were exclusive to XZJB and DM (Figure 6).

Gene Ontology (GO) analysis was conducted to functionally categorize DEGs among the comparison groups: GLG vs. XZJB, GLG vs. DM, and XZJB vs. DM. The top 20 GO terms were classified into three primary categories: biological processes, cellular components, and molecular functions. A substantial number of DEGs associated with metabolic and cellular processes were enriched across all three comparisons.

For GLG vs. XZJB and GLG vs. DM, GO-annotated DEGs were predominantly enriched in molecular functions, particularly in oxidoreductase activity and iron ion binding. In terms of cellular components, DEGs were enriched in the intrinsic component of membrane and membrane part, while in biological processes, the oxidation-reduction process was most prominent. In contrast, DEGs in the XZJB vs. DM comparison were primarily enriched in biological processes, especially the polysaccharide catabolic process (Supplementary Figure 3).

KEGG enrichment analysis was conducted to investigate the metabolic pathways associated with DEGs (Supplementary Figure 4). A total of 15 pathways were significantly enriched in GLG compared to XZJB (*P* < 0.05), of which 4 were highly significant (*P* < 0.001). Among the enriched DEGs, 11 were upregulated and 19 were downregulated. In the comparison between GLG and DM, 10 pathways were significantly enriched (*P* < 0.05), including 1 highly significant pathway (*P* < 0.001); 8 enriched DEGs were upregulated and 9 were downregulated. XZJB showed significant enrichment in 12 pathways compared to DM (*P* < 0.05), with 26 enriched DEGs upregulated and 11 downregulated.

The prodigiosin biosynthesis and biotin metabolism pathways were significantly enriched across all three comparison groups, with downregulation of the *fabG* gene, which is primarily involved in the biosynthesis of polyunsaturated fatty acids. The amino sugar and nucleotide sugar metabolism pathways were significantly enriched in both XZJB vs. GLG and DM vs. GLG, where two enriched DEGs, *E3.5.1.41* and *GME*, were upregulated. The acetoacetyl-CoA thiolases (AACT) gene was upregulated in several significantly enriched pathways in the DM group compared to the XZJB group. This gene catalyzes the conversion of two acetyl-CoA molecules into acetoacetyl-CoA in the mevalonate (MVA) pathway, which is the initial step in terpenoid biosynthesis (Supplementary Tables 3, 5). The integration of GO and KEGG pathway enrichment analyses, along with the expression patterns of DEGs, provides insights into the key pathways and genes involved in the biosynthesis of terpenoid compounds in *T. gaoligongensis*.

### Biosynthesis of antcins and antrodin C in *T. gaoligongensis*

**FIGURE 7:**
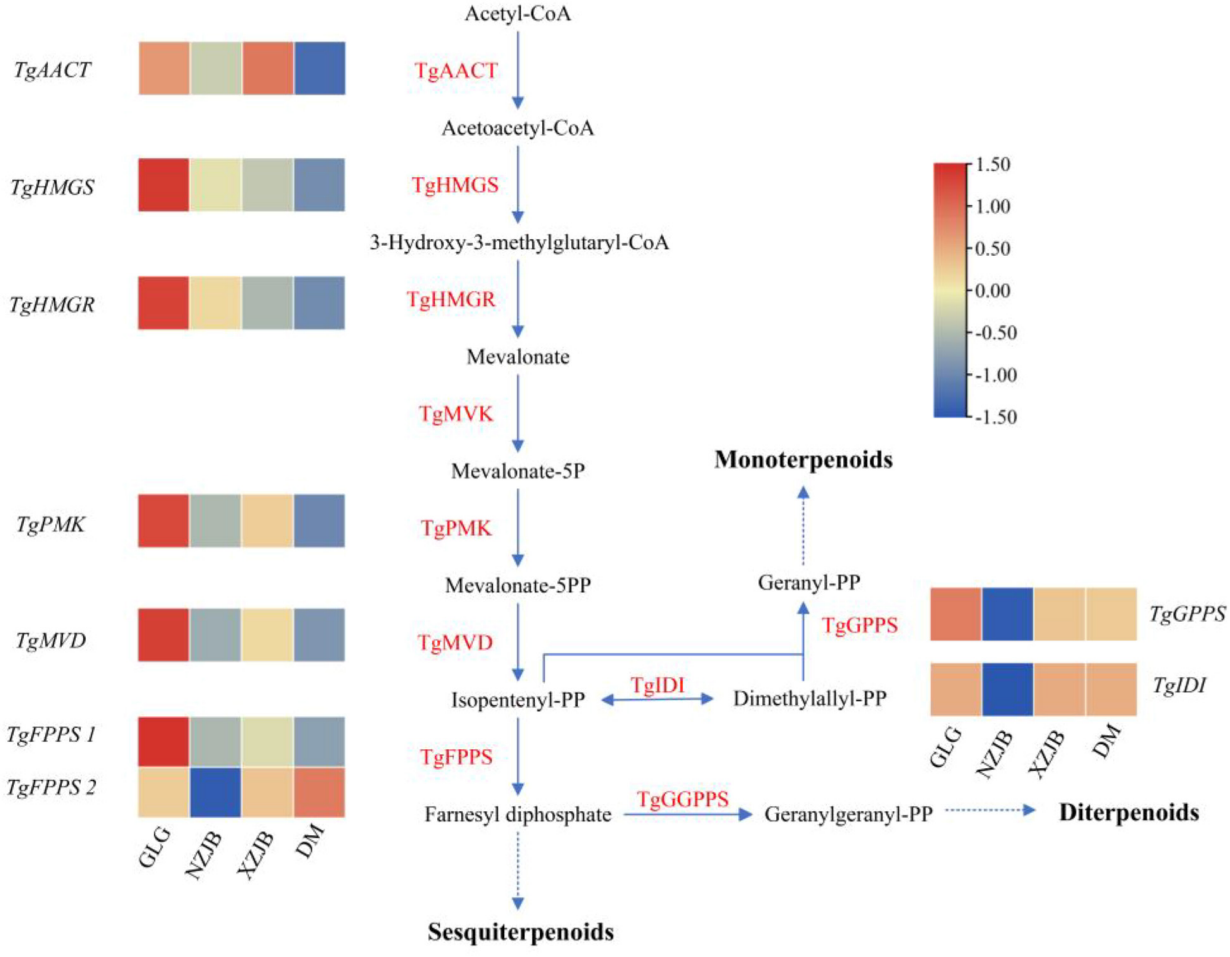
Enzymatic reactions in the mevalonate (MVA) pathway in *T. gaoligongensis*, and the expression of some key genes. GLG, *T. gaoligongensis* fruiting bodies; NZJB, mycelia cultured in fungal cultivation bags containing *C. kanehirae* substrate; XZJB, mycelia cultured in fungal cultivation bags containing *C. camphora* substrate; DM, mycelia cultured on rice medium.

The terpenoids biosynthetic pathway in *T. gaoligongensis* is illustrated in the figure, while the ergosterol biosynthetic pathway is adapted from that of *Saccharomyces cerevisiae*. The corresponding genes in *T. gaoligongensis* were obtained from the protein sequences of the corresponding genes in the NCBI database and the *T. gaoligongensis* protein database using local BLAST. Metabolomic data revealed that terpenoids levels were highest in GLG, followed by NZJB and XZJB, and lowest in DM, with expression levels of the associated terpenoid biosynthetic genes showing corresponding trends. Notably, two *TgFPPS* genes were identified: *TgFPPS 1* was most highly expressed in GLG, whereas *TgFPPS 2* exhibited the highest expression in DM. These findings suggest the existence of two distinct biosynthetic pathways for the conversion of isopentenyl diphosphate (IPP) to farnesyl diphosphate (FPP) (Figure 7).

**FIGURE 8:**
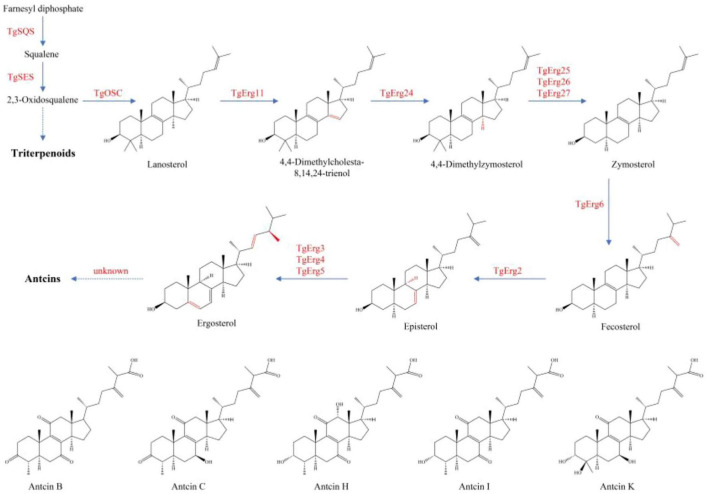
Triterpenoid and ergosterol biosynthetic pathways in *T. gaoligongensis*, and the structures of antcins detected in the metabolome.

**FIGURE 9:**
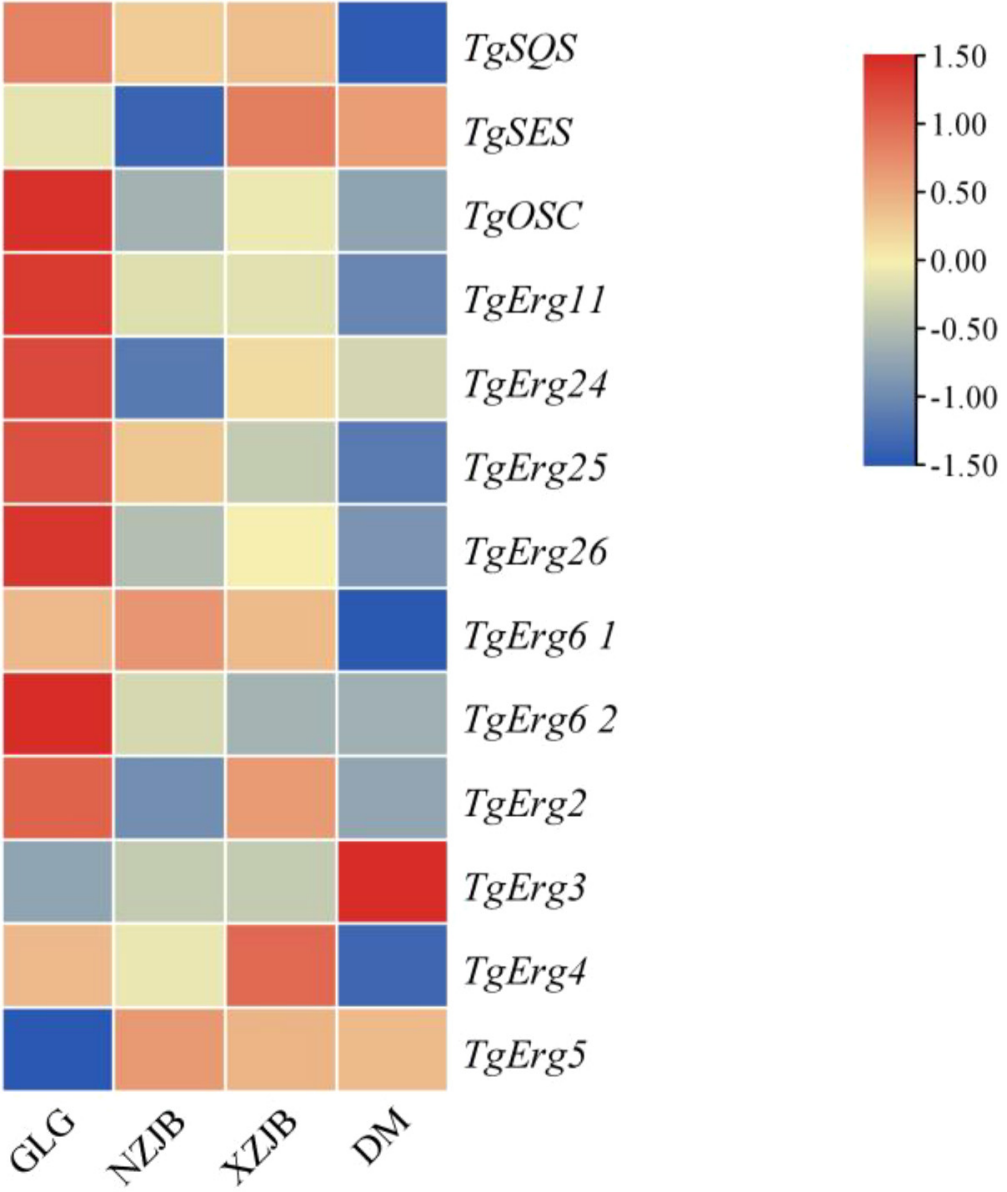
Differential expression of triterpenoids and ergosterol biosynthetic genes in *T. gaoligongensis* across different samples. GLG, *T. gaoligongensis* fruiting bodies; NZJB, mycelia cultured in fungal cultivation bags containing *C. kanehirae* substrate; XZJB, mycelia cultured in fungal cultivation bags containing *C. camphora* substrate; DM, mycelia cultured on rice medium.

Antcins and terpenoids displayed similar patterns of variation across samples, although their levels were higher in XZJB than in NZJB. In addition to antcins, eleven ergosterol derivatives were detected in the metabolome, with several showing high abundance in DM (Supplementary Figure 12). Currently, the exact biosynthetic pathway of ergosterol derivatives remains unidentified, though it is hypothesized to be associated with ergosterol biosynthesis. Gene expression changes in *T. gaoligongensis* were more closely aligned with variations in antcins content across the samples, supporting the hypothesis that ergosterol biosynthesis may be linked to antcin production, and that ergosterol may serve as precursors for antcins (Figures 8, 9). However, *TgSES*, *TgErg3*, and *TgErg5* were expressed at lower levels in GLG and higher levels in DM, possibly explaining the greater abundance of certain ergosterol derivatives in DM. Antrodin C content was highest in NZJB, where its level was 3.2-fold greater than in *T. gaoligongensis* fruiting bodies (Figure 6). This may be associated with higher expression of *TgHMGR* and *wrbA*, genes involved in antrodin C biosynthesis, in NZJB (Supplementary Figure 11).

**FIGURE 10:**
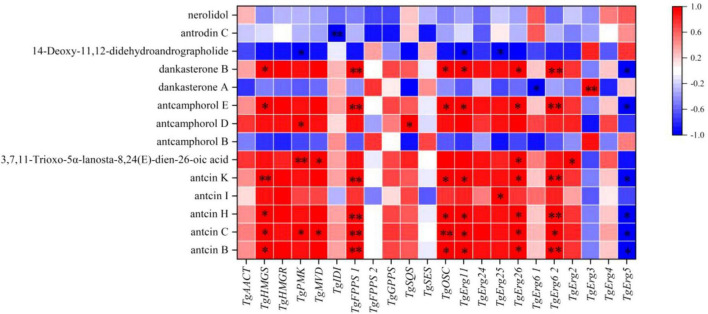
Investigation of the correlation between terpenoid biosynthetic Genes and terpenoid metabolites in *T. gaoligongensis*.

The Pearson correlation coefficient was used to evaluate the relationships between terpenoid biosynthetic genes and terpenoid metabolites in *T. gaoligongensis*. The terpenoid biosynthetic genes were identified through local BLAST searches and gene annotation, while documented terpenoid metabolites previously reported in *T. camphoratus* were selected from the metabolomic data for comparison. Significant positive correlations were observed between Antcin B, antcin C, antcin H, and antcin K and the genes *TgHMGS, TgFPPS1, TgOSC, TgErg11, TgErg26*, and *TgErg62*. In addition, antcin C also showed significant positive correlations with *TgPMK* and *TgMVD*. Antcin I was significantly positively correlated with *TgErg25* (Figure 10). These results indicate that terpenoid biosynthetic genes play a critical role in the biosynthesis of terpenoid metabolites, particularly antcins, in *T. gaoligongensis*.

### Quantitative real-time PCR analysis

**FIGURE 11:**
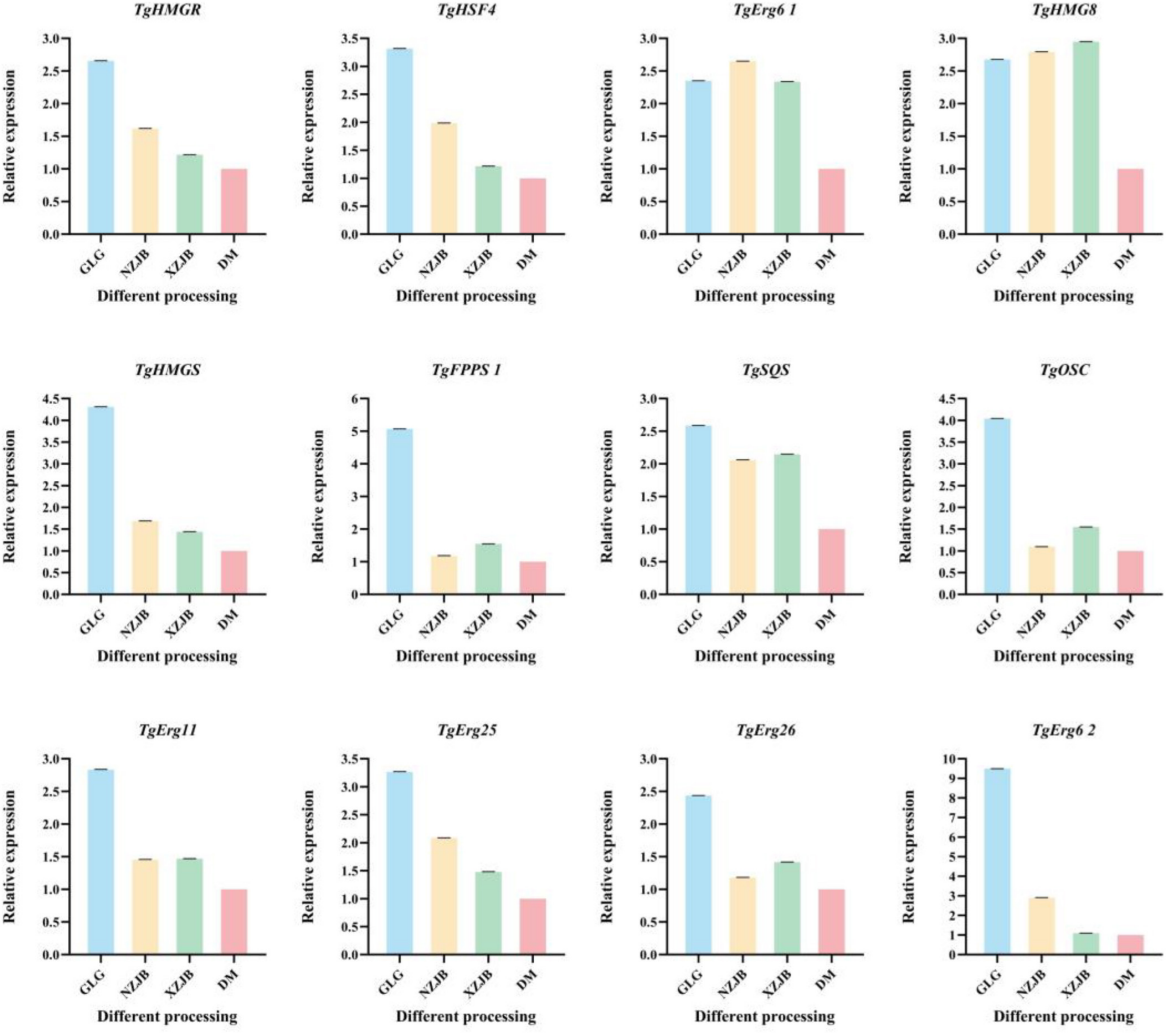
Relative expression of DEGs by qRT–PCR. GLG, *T. gaoligongensis* fruiting bodies; NZJB, mycelia cultured in fungal cultivation bags containing *C. kanehirae* substrate; XZJB, mycelia cultured in fungal cultivation bags containing *C. camphora* substrate; DM, mycelia cultured on rice medium.

The expression levels of 12 DEGs and *tfs* associated with terpenoids metabolism in *T. gaoligongensis* were analyzed using qRT-PCR to validate the transcriptome sequencing results. The findings indicated that the relative expression trends of these genes were consistent with those observed in the transcriptome data. Specifically, the relative expression levels of *TgHMGR* and *TgHSF4* under different cultivation methods followed the order: GLG > NZJB > XZJB > DM. The expression levels of *TgErg6 1* and *TgHMG8* were similarly elevated in GLG, NZJB, and XZJB, and significantly higher than those in DM. In contrast, the expression levels of *TgHMGS*, *TgFPPS 1*, *TgSQS*, *TgOSC*, *TgErg11*, *TgErg25*, *TgErg26*, and *TgErg6 2* were lower in NZJB and XZJB than in GLG but higher than in DM (Figure 11). These results suggest that cultivation methods have a significant influence on the expression patterns of terpenoids biosynthesis-related genes in *T. gaoligongensis*. Different cultivation environments may modulate the activation of terpenoids metabolic pathways and consequently affect the synthesis of various bioactive terpenoids in *T. gaoligongensis*.

### Prediction of *TgTFs* binding sites in the promoter regions of terpenoid and ergosterol synthesis genes

**FIGURE 12:**
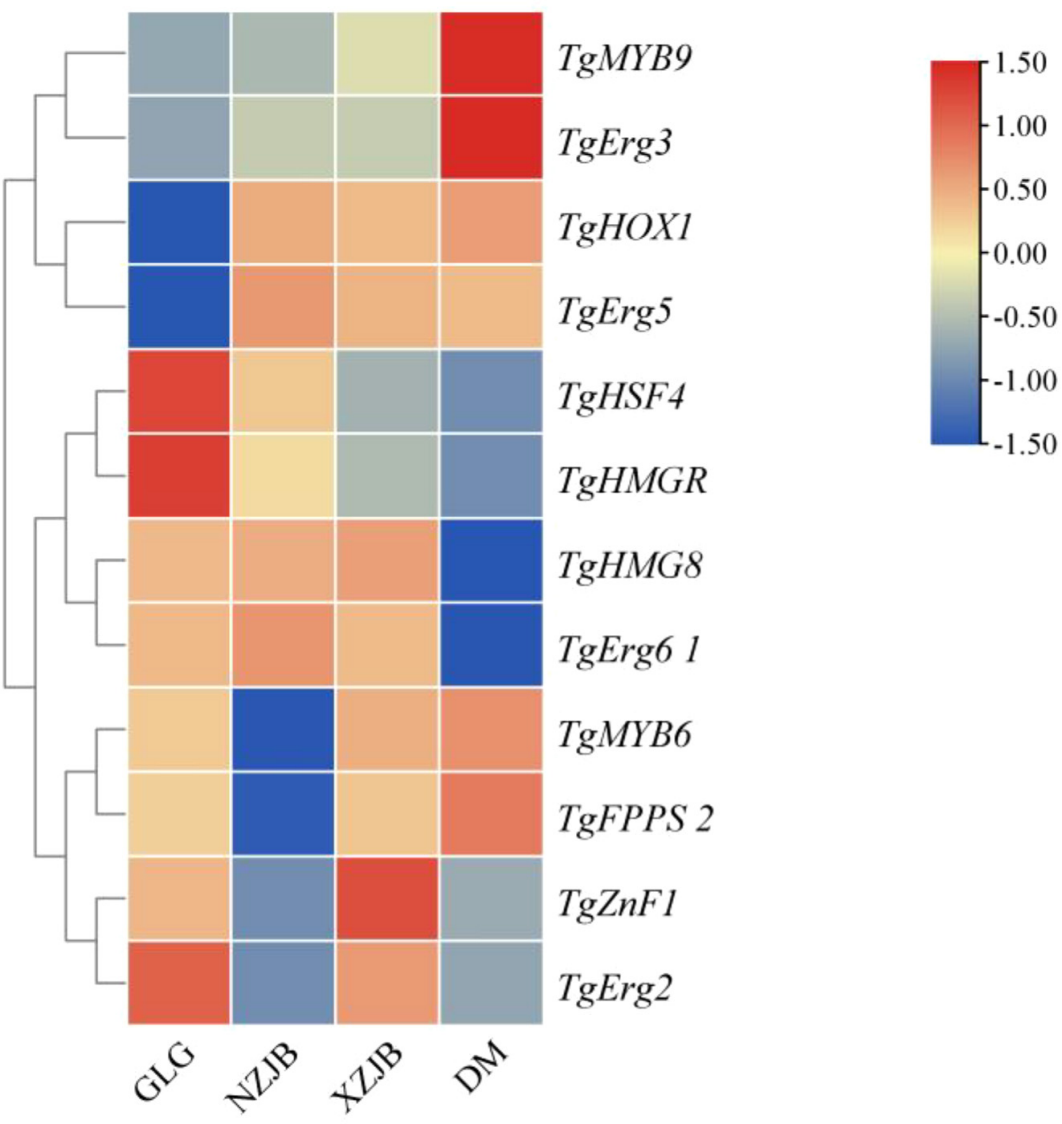
Interactive heatmap of gene expression for terpenoid and ergosterol synthesis genes, and co-expressed *TgTFs* under varying cultivation methods. GLG, *T. gaoligongensis* fruiting bodies; NZJB, mycelia cultured in fungal cultivation bags containing *C. kanehirae* substrate; XZJB, mycelia cultured in fungal cultivation bags containing *C. camphora* substrate; DM, mycelia cultured on rice medium.

The synergistic regulation of terpenoids and ergosterol biosynthetic genes by *TFs* was investigated. Co-expression trend analysis (Figure 12) and binding site prediction of *TgTFs* in the promoter regions of these genes identified several potential binding sites with high relative scores, suggesting their involvement in the regulation of terpenoids and ergosterol biosynthesis. The predicted binding sites and their corresponding scores are presented in Table 3. Notably, *TgHSF4* and *TgMYB6* may regulate the transcriptional activities of *TgHMGR* and *TgFPPS 2*, respectively. In addition, the expression of *TgErg2*, *TgErg3*, *TgErg5*, and *TgErg6 1* may be regulated by *TgZnF1*, *TgMYB9*, *TgHOX1*, and *TgHMG8*, respectively. These findings suggest that the identified *TgTFs* may bind to DNA sequences in the promoter regions of their respective target genes, potentially promoting their transcription.

**TABLE 3:** Binding sites of co-expressed *TgTFs* in the promoter regions of terpenoid and ergosterol synthesis genes.

Matrix ID	Score	Relative score	Sequence ID	Start	End	Strand	Predicted sequence
TgHMG8	8.14735	0.90543175	TgErg6_1	507	512	+	actaga
TgZnF1	8.91578	1.00000001	TgErg2	654	659	−	atccac
TgMYB6	11.1998	0.99616075	TgFPPS_2	1613	1619	−	aacccac
TgMYB9	11.7046	0.92568517	TgErg3	818	827	−	tccgcatcgc
TgHSF4	8.40227	0.99324024	TgHMGR	1602	1607	−	ggccag
TgHOX1	8.92601	0.96724313	TgErg5	1918	1925	−	cagaaatt

## Discussion

The application of multi-omics research methods has gained increasing importance in fungal studies (Shankar and Sharma, 2022). By integrating high-throughput technologies such as genomics, transcriptomics, metabolomics, and proteomics, multi-omics approaches offer novel perspectives for the in-depth analysis of biosynthetic pathways of bioactive metabolites in fungi, their functional roles in cellular and biological processes, and their regulatory mechanisms. This strategy not only facilitates the discovery of biosynthetic gene clusters (BGCs) encoding novel natural products but also enables elucidation of the regulatory mechanisms underlying their biosynthesis, representing an effective means for mining potential bioactive compounds in microorganisms (Palazzotto and Weber, 2018). Among multi-omics technologies, untargeted metabolomics serves as a systematic and high-throughput tool for metabolite profiling, capable of revealing the effects of exogenous variables—such as cultivation methods, co-culture environments, or stress factors—on fungal secondary metabolite biosynthesis. This provides a theoretical basis for optimizing cultivation strategies and enhancing the production efficiency of target metabolites. When combined with transcriptomic, genomic, and other omics data, untargeted metabolomics can further resolve key metabolic pathways and clarify the biosynthetic mechanisms of specific secondary metabolites (Gertsman and Barshop, 2018). For example, in wood-decaying basidiomycetes, multi-omics analyses have revealed the oxidative-hydrolytic metabolic strategy adopted by *Laetiporus sulphureus* for lignocellulose degradation (de Figueiredo et al., 2021); Additionally, untargeted metabolomics was used for confirmation that the addition of methanolic extracts from *C. kanehirae* trunks significantly enhanced terpenoids production during the deep fermentation of *T. camphoratus* (Luo et al., 2023); Furthermore, combined genomic and untargeted metabolomic analyses have uncovered the chemical diversity of terpenoids in fungi of the genus *Suillus* (Mudbhari et al., 2024). In the present study, we employed untargeted metabolomics to reveal significant differences in the metabolic profiles of *T. gaoligongensis* under different cultivation methods. By integrating reference-guided transcriptomic analysis, we further explored the biosynthetic pathways of key terpenoids—including antrodin C and antcins—thus providing valuable insights into their transcriptional regulation and biosynthetic mechanisms.

Genomic studies have revealed inconsistencies between the number of gene clusters predicted to encode secondary metabolites using bioinformatics tools and the actual secondary metabolites produced by microorganisms. Many of these gene clusters are considered “silent,” as their expression is often inactive under standard laboratory conditions but can be triggered by altering culture parameters—an effective strategy for enhancing microbial secondary metabolite production. This phenomenon is summarized by the concept of “one strain, many compounds” (OSMAC; Romano et al., 2018). *T. gaoligongensis* is a slow-growing species that is rare in the wild. However, artificial cultivation, substrate optimization, and the introduction of specific inducers can significantly enhance the diversity and yield of bioactive compounds. For instance, the addition of corn oil, linolenic acid, or *C. kanehirae* extracts to liquid fermentation systems has been shown to induce triterpenoid biosynthesis (Meng et al., 2021; Tang et al., 2024; Luo et al., 2023). Additionally, the production of antrodin C can be effectively stimulated by various strategies, including pH modulation, sugar supplementation, inositol addition, oleic acid extraction, and particulate enhancement (Zhang et al., 2014; Jia et al., 2023; Liu et al., 2020; Liu et al., 2019; Fan et al., 2023). In the present study, the inclusion of sawdust from *C. kanehirae* and *C. camphora* branches in solid-state fermentation fungal cultivation bags markedly enhanced terpenoids production, including antrodin C and antcins. Terpenoid-rich components in *C. kanehirae* and *C. camphora* are likely key contributors to this increase. It is hypothesized that certain terpenoids from these substrates may participate directly in the terpenoid metabolic pathway of *T. gaoligongensis*. The identification of these compounds, their chemical structures, and their metabolic transformations will be the focus of future investigations.

Previous studies have demonstrated that the addition of *C. kanehirae* extract to the deep fermentation of *T. camphoratus* enhances the production of its bioactive compounds, such as terpenoids, polysaccharides, and andrographolide. However, characteristic triterpenoids like antcins from *T. camphoratus* fruiting bodies were not detected in the metabolomic profiles (Luo et al., 2023; Hu et al., 2016). In the present study, the solid-state fermentation fungal bag cultivation system yielded higher levels of terpenoids. Many of these compounds have been previously reported in *T. camphoratus* or other fungi, supporting the credibility of the data. Antcins in *T. gaoligongensis* mycelium were significantly more abundant in the fungal bag cultivation system than in the rice medium (DM). Moreover, in addition to antcins, 13 metabolites previously reported in *T. camphoratus* were detected, including two sesquiterpenoids—nerolidol and antrodin C—and one lignan, sesamin. These compounds were significantly more concentrated in the fungal bag cultivation system, especially those containing *C. kanehirae* Substrate. Nerolidol, which exhibits antioxidant, antibacterial, antitumor, and anti-inflammatory activities, holds promise as a novel therapeutic or agrochemical agent (Chan et al., 2016). Sesamin possesses potent immunoprotective and anti-inflammatory properties and demonstrates therapeutic potential for cardiovascular diseases and diabetes (Dalibalta et al., 2020; Majdalawieh et al., 2022). In addition, 22 triterpenoids known to be produced in other fungi, such as *Ganoderma lucidum* and *Poria cocos*, were also identified. Among these, nine have documented bioactivities, including anticancer, anti-inflammatory, and antimicrobial effects (Supplementary Table 2). The variation in their abundance across samples, particularly in the fungal bag cultivation system containing *C. kanehirae* substrate, suggests that this cultivation method strongly promotes their biosynthesis (Supplementary Figure 1). These findings indicate that the modified culture system not only enhances the biological activity of mycelia but also enables more efficient and large-scale cultivation compared to traditional fruiting bodies cultivation on *C. kanehirae* wood logs.

Using local BLAST searches against known genes involved in terpenoids and ergosterol biosynthesis, candidate biosynthetic genes for terpenoids—including antcins and antrodin C—were identified in *T. gaoligongensis* based on differential genes expression patterns and variation in metabolite profiles across various cultivation methods. These findings provide a foundation for subsequent functional validation of key genes and the large-scale production of bioactive terpenoids. Enhancing the expression of critical biosynthetic genes in the mevalonate (MVA) pathway is an effective strategy to increase terpenoid yields. In *T. gaoligongensis*, terpenoids and ergosterol biosynthesis genes generally exhibited higher expression in GLG. Among them, *HMGR* is a well-established rate-limiting enzyme (Han et al., 2018; Dai et al., 2012), and *TgHMGR* showed significantly higher expression in the NZJB group than in the XZJB and DM groups. Previous studies have implicated both *HMGR* and *wrbA* in the biosynthesis of antrodin C (Liu et al., 2020; Jia et al., 2023), and in this study, both genes also exhibited higher expression in NZJB. Notably, the gene expression patterns observed under different cultivation methods correlated well with the corresponding changes in metabolite levels.

Basidiomycetes exhibit distinct gene expression profiles at different developmental stages, resulting in variations in the types and concentrations of terpenoids and other bioactive compounds produced (Borgognone et al., 2018; Vonk and Ohm, 2021). Therefore, further investigation into the gene expression and metabolic pathways of *T. gaoligongensis* at different developmental stages may help elucidate the transcriptional regulatory mechanisms underlying the biosynthesis of its active metabolites. Although numerous bioactive compounds have been isolated and characterized from *T. camphoratus*, only 18 previously reported compounds were detected in the present study. This discrepancy may be attributed to the inherent limitations of untargeted metabolomics. Specifically, untargeted metabolomics faces challenges in compound identification due to incomplete reference databases, the presence of isomers, and isomerization phenomena. Moreover, it cannot precisely quantify absolute compound concentrations, as it only allows for the comparison of relative abundances across samples, which compromises both data accuracy and reproducibility (Beger et al., 2019). In contrast, targeted metabolomic analysis of individual compounds extracted and purified from *T. gaoligongensis* provides a more accurate approach, enabling the determination of absolute concentrations. Furthermore, the terpenoids biosynthetic genes identified in this study may be functionally validated in future work and heterologously expressed in engineered microbial systems to enhance the production of bioactive terpenoids from *T. gaoligongensis* (Dinday and Ghosh, 2023).

## Conclusion

In this study, we systematically evaluated the effects of different cultivation methods on terpenoids biosynthesis in *T. gaoligongensis* using integrated multi-omics analysis. The results demonstrated that terpenoids accumulation was significantly enhanced in fungal cultivation bags containing *C. kanehirae* substrate (NZJB) and *C. camphora* substrate (XZJB). In particular, the content of antcins, especially antcin C and antcin I, was markedly increased in these cultures. Antrodin C levels were highest in NZJB, reaching 3.2 times the level observed in the fruiting bodies cultivation on *Cinnamomum kanehirae* wood logs. Transcriptomic analysis revealed differential expression of genes associated with the biosynthesis of antcins and antrodin C under various cultivation methods, confirming that *TgHMGR* is likely a key rate-limiting enzyme in the terpenoids biosynthetic pathway of *T. gaoligongensis*. Further co-expression network and binding site prediction analyses suggested that transcription factors *TgHSF4*, *TgMYB6*, *TgZnF1*, *TgMYB9*, *TgHOX1*, and *TgHMG8* may play critical roles in the transcriptional regulation of terpenoids biosynthesis. Specifically, *TgHSF4* and *TgMYB6* may regulate the transcription of *TgHMGR* and *TgFPPS 2*, respectively, while *TgZnF1*, *TgMYB9*, *TgHOX1*, and *TgHMG8* may influence the expression of *TgErg2*, *TgErg3*, *TgErg5*, and *TgErg6 1*, respectively. These findings provide valuable insights into the metabolic regulation and potential industrial application of *T. gaoligongensis*.

## Data Availability

Data associated with this study has been deposited at GenBank and Sequence Read Archive (Accession number: SRR33332703, SRR33333746, SRR33333745, SRR33333744), National Center for Biotechnology Information database.

## Footnotes

1
http://www.hmdb.ca

2
https://massbank.eu

3
http://www.lipidmaps.org

4
https://www.mzcloud.org

5
https://www.ncbi.nlm.nih.gov/datasets/genome/GCA_037127245.1/

6
https://blast.ncbi.nlm.nih.gov/

7
https://meme-suite.org/meme/tools/meme
