# Supplementary material for: Multi-omics analysis of Taiwanofungus gaoligongensis: effects of different cultivation methods on secondary metabolites
Source: Front Microbiol. 2025 Aug 1;16:1620693. doi: 10.3389/fmicb.2025.1620693 (PMC12355660; doi:10.3389/fmicb.2025.1620693)
Supplement: Supplementary file 1 [file Supplementary_file_1.zip › Supplementary file 1/Table 1.DOCX]

Supplementary material

FIGURE S1

Heatmap of the concentrations of bioactive triterpenoids, previously reported to be produced by fungi other than *T. camphoratus*, detected through metabolomic analysis.

FIGURE S2

Gene expression correlation analysis among different samples of T. gaoligongensis.

FIGURE S3

GO terms and the -log₁₀(P-value) of GO term enrichment in the comparisons: (A) XZJB vs. GLG, (B) DM vs. GLG, and (C) DM vs. XZJB. Figure S6. KEGG bubble plots for (A) XZJB vs. GLG, (B) DM vs. GLG, and (C) DM vs. XZJB.

FIGURE S4-S10

Phylogenetic and conserved motif analyses of TgErg24, TgErg26, TgErg6, TgErg3, TgErg4, and TgErg5 in comparison with their homologs from selected Basidiomycota, Ascomycota, and S. cerevisiae.

FIGURE S11

Heatmap of gene expression related to antrodin C synthesis in various *T. gaoligongensis* samples.

FIGURE S12

Differential levels of ergosterol derivatives in the metabolome of T. gaoligongensis.

TABLE S1

Antcins in different samples of T. gaoligongensis.

TABLE S2

Metabolomic detection of triterpenoids produced by fungi reported in the literature other than *T. camphoratus*.

TABLE S3-S5

Annotations of the KEGG pathway for DEGs in the comparisons: (A) XZJB vs. GLG, (B) DM vs. GLG, and (C) DM vs. XZJB.

TABLE S6

Ergosterol biosynthesis genes of T. gaoligongensis.

TABLE S7

The identity and similarity between ergosterol biosynthesis enzymes of *T. gaoligongensis* and the corresponding enzymes of *T. camphoratus*.

TABLE S8

Primer list of genes for qRT-PCR.

TABLE S9

The gene sequence of the synthetic genes.

TABLE S10

Expression matrix for all samples and all metabolites.

TABLE S11

RNA-Seq expression matrix for all samples and all genes.

TABLE S1 Antcins in different samples of *T. gaoligongensis.*

| Identity | Triterpenoid Types | Formula | Annot.DeltaMass [ppm] | Calc.MW | m/z | RT[min] |
| --- | --- | --- | --- | --- | --- | --- |
| Antcin B, S | Ergostanes | C_29_H_40_O_5_ | 1.02 | 468.2881 | 467.2808 | 6.20 |
| Antcin B, R | Ergostanes | C_29_H_40_O_5_ | -2.65 | 468.2863 | 467.2810 | 6.47 |
| Antcin C, S | Ergostanes | C_29_H_42_O_5_ | -2.39 | 470.3021 | 469.2945 | 6.70 |
| Antcin C, R | Ergostanes | C_29_H_42_O_5_ | 1.01 | 470.3037 | 469.2964 | 7.60 |
| Antcin K, S+R | Ergostanes | C_29_H_44_O_6_ | -0.44 | 488.3136 | 487.3063 | 5.56 |
| Antcin H, S+R | Ergostanes | C_29_H_42_O_6_ | 0.18 | 486.2982 | 485.2909 | 7.63 |
| Antcin I, S+R | Ergostanes | C_29_H_42_O_5_ | -3.01 | 470.3018 | 469.2949 | 7.08 |

| **Name** | **Formula** | **Triterpenoid Types** | **Sourse** | **Bioactivity** |
| --- | --- | --- | --- | --- |
| Arjunolic acid | C30H48O5 | pentacyclic triterpenoidal saponin | *Xylaria papulis*(1) | antioxidant, anti-fungal, anti-bacterial, anticholinesterase, antitumor, antiasthmatic, cardioprotective agent, wound healing and insect growth inhibitor activities(2) |
| 20-Hydroxylucidenic acid E2 | C29H40O9 | lanostane-type triterpene acid | *Ganoderma lucidum*(3) | inhibitory effects on EBV-EA induction(3) |
| Fasciculic acid B | C36H60O9 | triterpenoid | *Naematoloma fasciculare*(4) | calmodulin antagonists(4) |
| Spongiporic acid A | C31H44O6 | triterpenoid | *Spongiporus leucomallellus*(5) | antibacterial and antifungal(5) |
| Enoxolone | C30H46O4 | pentacyclic triterpenoid | *Laetiporus sulphureus*(6) | anti-inflammatory, antibacterial, antioxidant(7, 8) |
| 6,7-dehydroporicoic acid H | C31H46O5 | lanostane-type triterpene acid | *Poria cocos*(9) | inhibitory effects on EBV-EA induction(10) |
| Ganoderic acid A | C30H44O7 | highly oxygenated lanostane-type triterpenoid | *Ganoderma lucidum*(11) | promotion of apoptosis; inhibit the release of histamine and enhance the functioning of digestive organs in humans; reduce blood fat, decrease blood pressure and regulate liver functions(11) |
| Lucidenic acid C | C27H42O6 | tetracyclic triterpenoid | *Ganoderma lucidum*(12) | anti-cancer effect(12) |
| methyl lucidenate Q | C28H42O6 | triterpenoid | *Ganoderma lucidum*(13) | inhibitory effects on EBV-EA induction(13) |
| Ganoderiol I | C31H50O5 | triterpenoids | *Ganoderma lucidum* |  |
| Ganodernoid B | C25H34O6 | lanostane nortriterpenoid | *Ganoderma lucidum*(14) |  |
| Ganolactone B | C27H38O6 | lanostane-type triterpenoid | *Ganoderma lucidum*(15) |  |
| Ganorbiformin F | C33H50O6 | lanostane-type triterpenoid | *Ganoderma lucidum*(16) |  |
| Hainanic acid A | C30H44O5 | lanostane-type triterpenoid | *Ganoderma hainanense*(17) |  |
| Poricoic acid E | C30H44O6 | triterpenoid | *Poria cocos* |  |
| Poricoic acid I | C31H46O6 | triterpenoid | *Poria cocos* |  |
| Poricoic acid K | C31H48O7 | triterpenoid | *Poria cocos* |  |
| Poricoic acid L | C31H46O7 | triterpenoid | *Poria cocos* |  |
| Irpeksolactin H | C30H46O6 | triterpenoid | *Irpex lacteus*(18) |  |
| Pisosteral | C33H52O5 | lanostane-type triterpenoid | *Pisolithus tinctorius*(19) |  |
| Fomefficinic acid F | C31H48O5 | triterpenoid | *Fomes officinalis*(20) |  |
| Forpinic acid C | C31H46O5 | C31 triterpenoid and triterpene sugar ester | *Fomitopsis pinicola*(21) |  |

TABLE S2 Metabolomic detection of triterpenoids produced by fungi reported in the literature other than *T. camphoratu.*


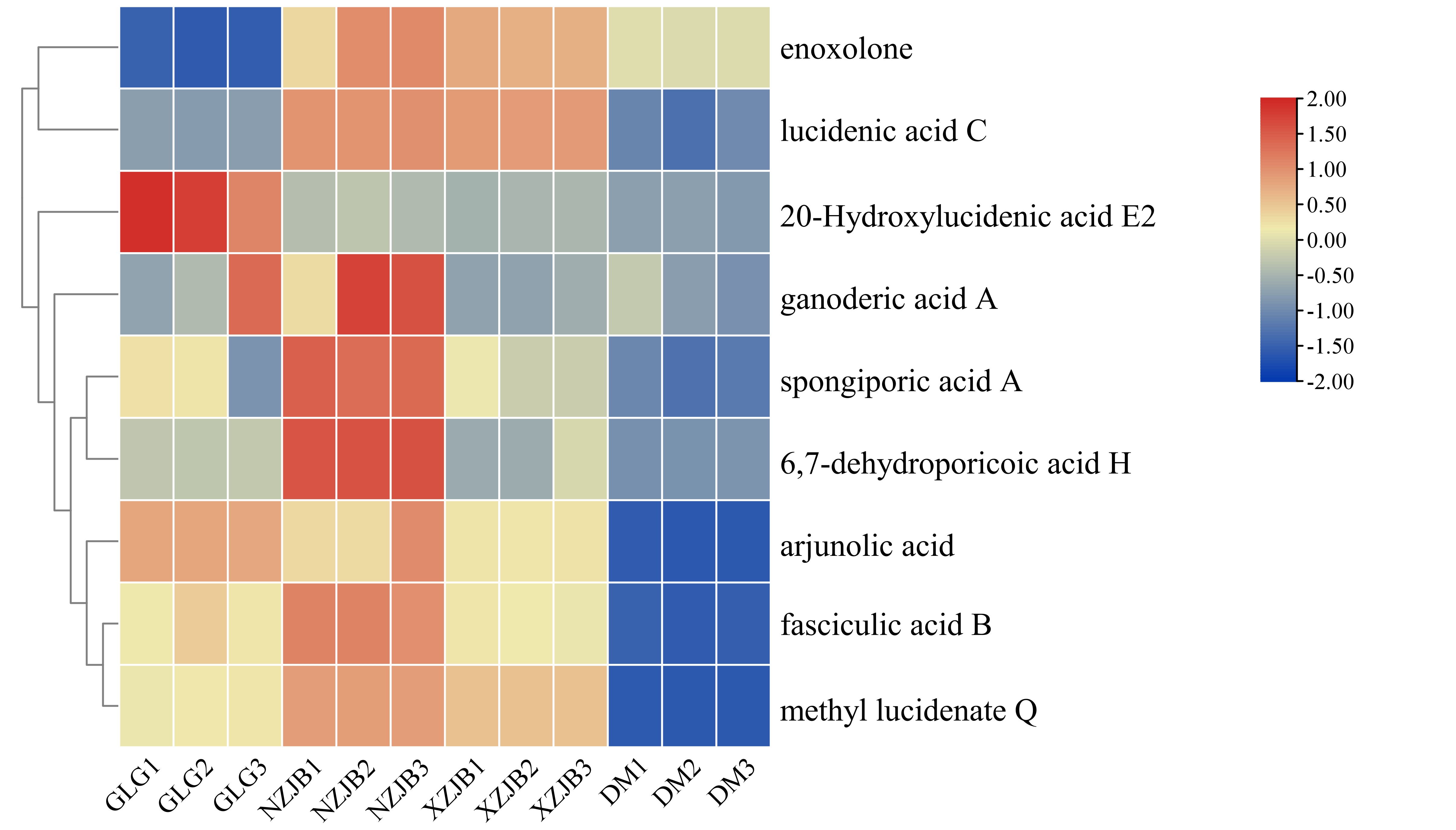


FIGURE S1

Heatmap of the concentrations of bioactive triterpenoids, previously reported to be produced by fungi other than *T. camphoratus*, detected through metabolomic analysis. GLG, *T. gaoligongensis* fruiting bodies; NZJB, mycelia cultured in fungal cultivation bags containing *C. kanehirae* substrate; XZJB, mycelia cultured in fungal cultivation bags containing *C. camphora* substrate; DM, mycelia cultured on rice medium.


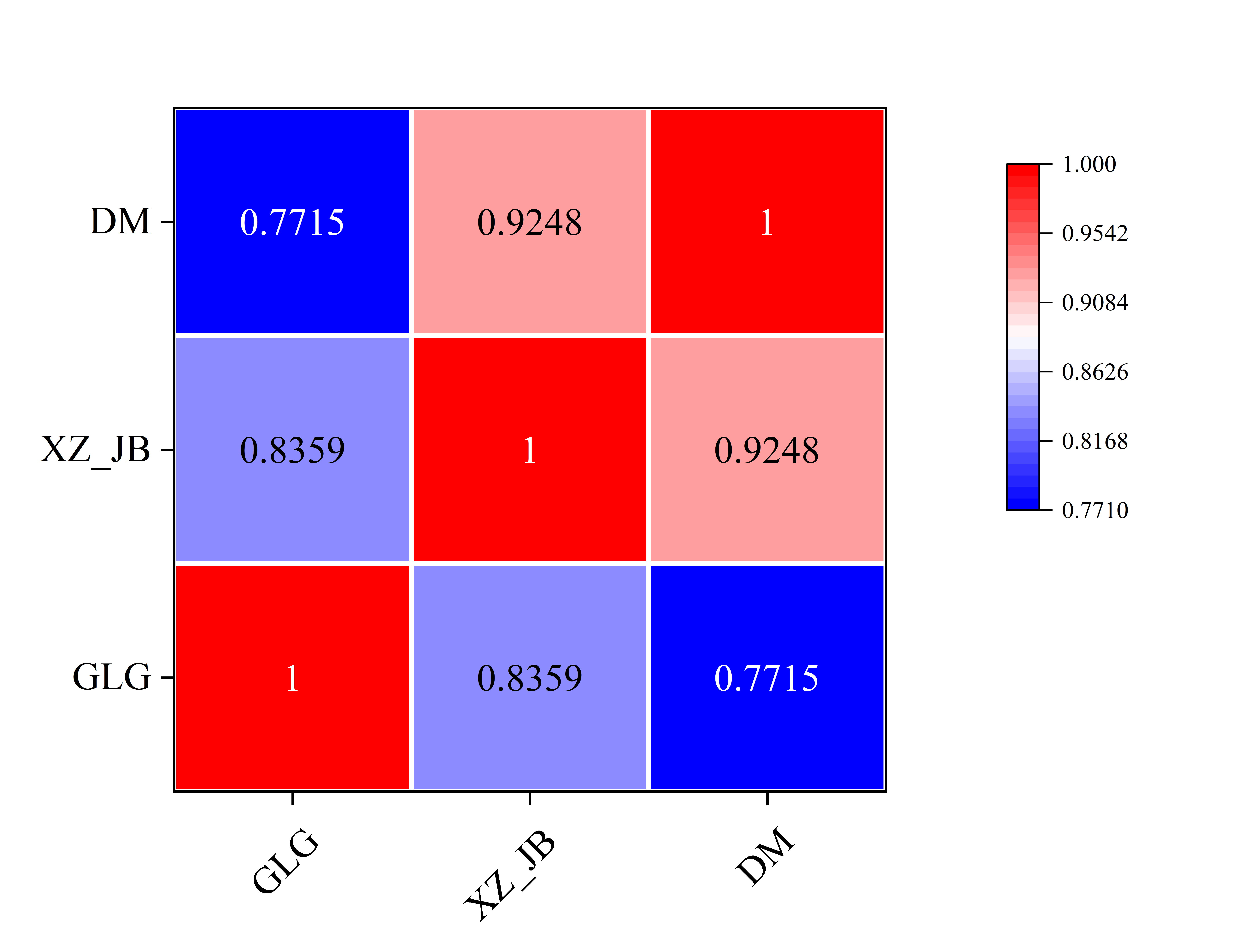


FIGURE S2

Gene expression correlation analysis among different samples of *T. gaoligongensis*.

B

C

A


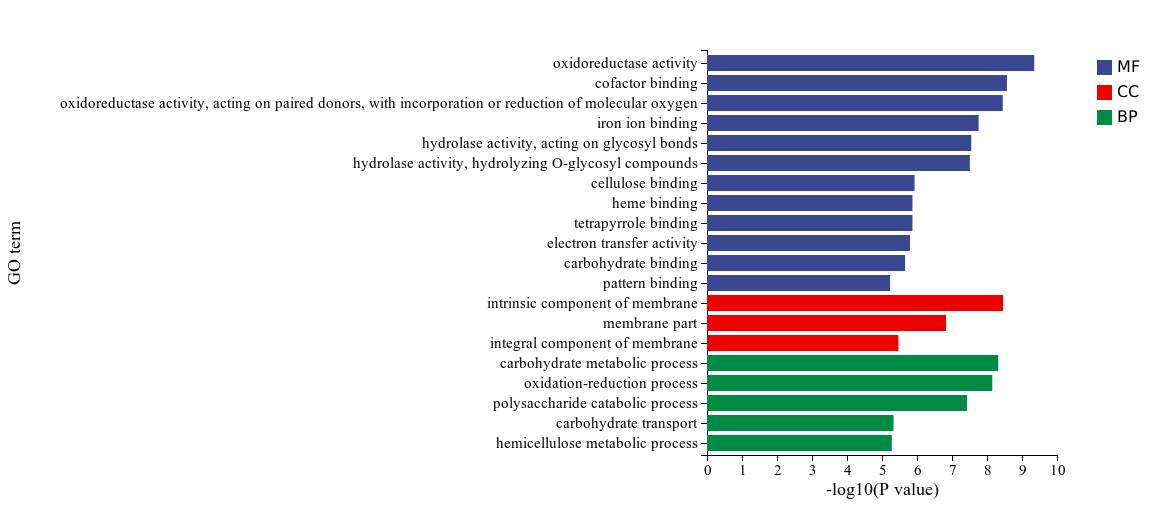


B


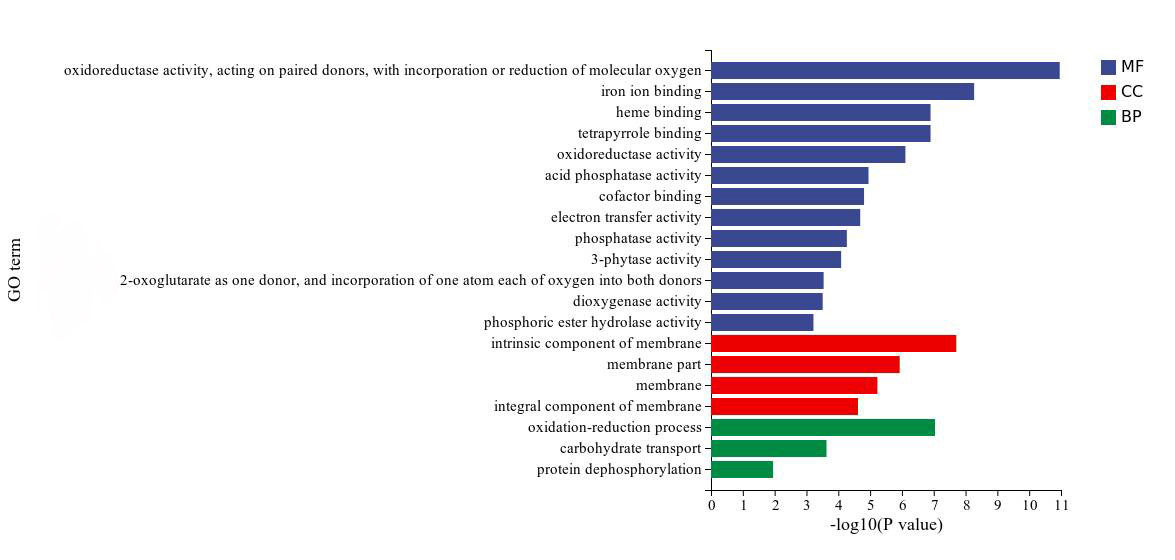


C


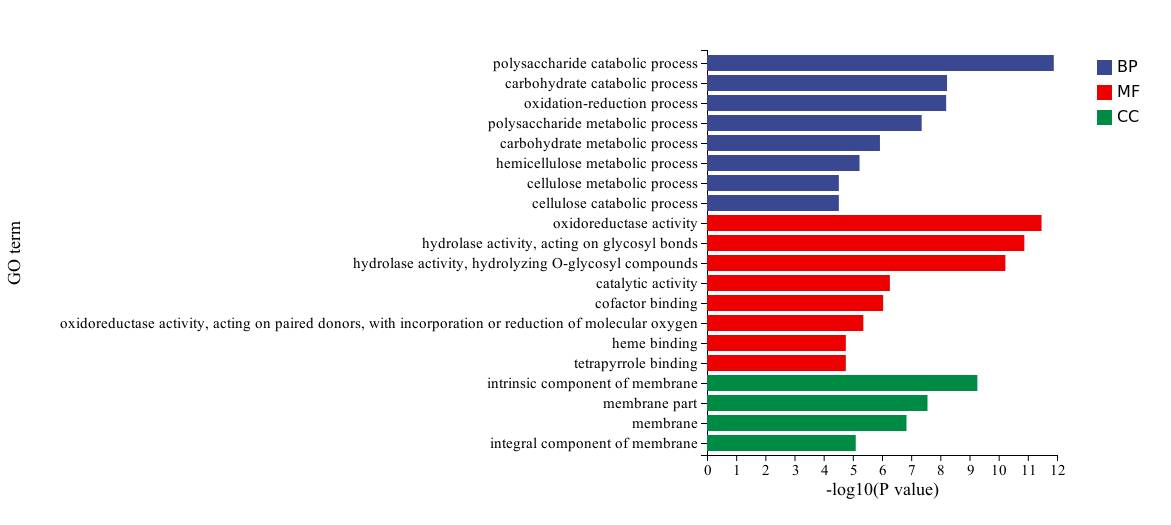


FIGURE S3

GO terms and the -log₁₀(P-value) of GO term enrichment in the comparisons: (A) XZJB vs. GLG, (B) DM vs. GLG, and (C) DM vs. XZJB.

A


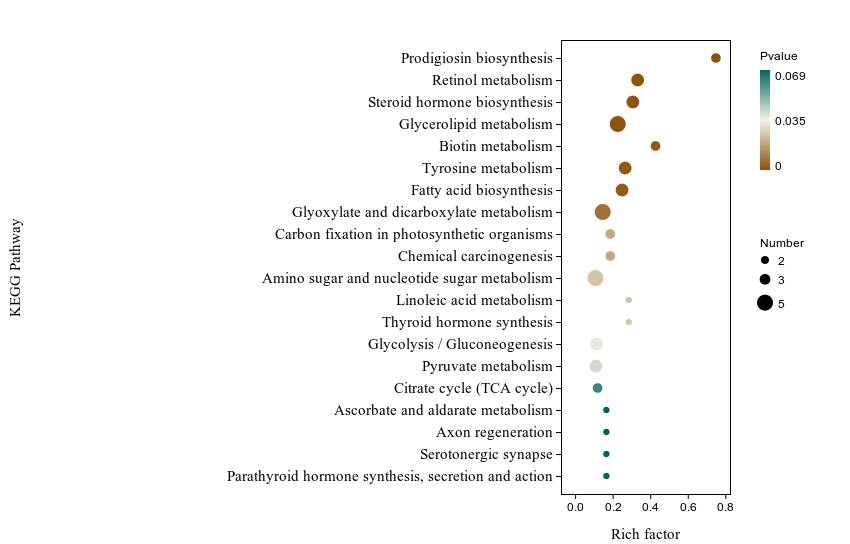


B


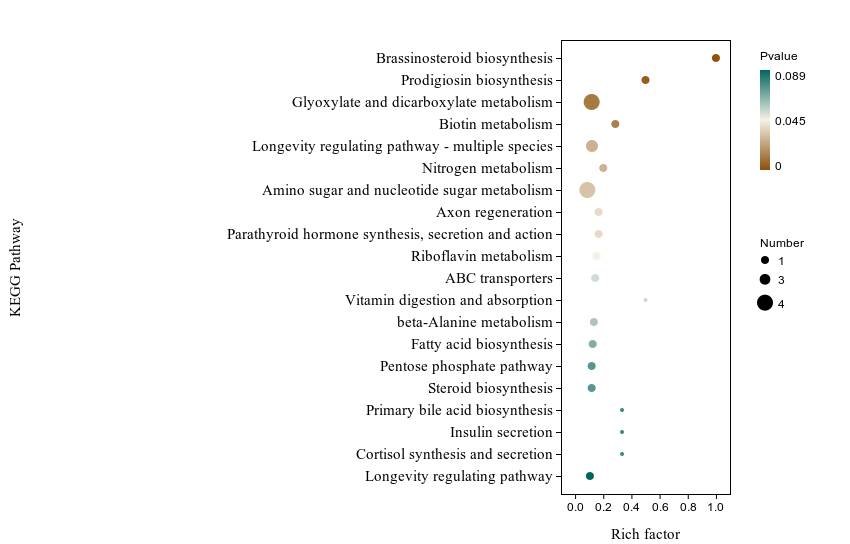


C


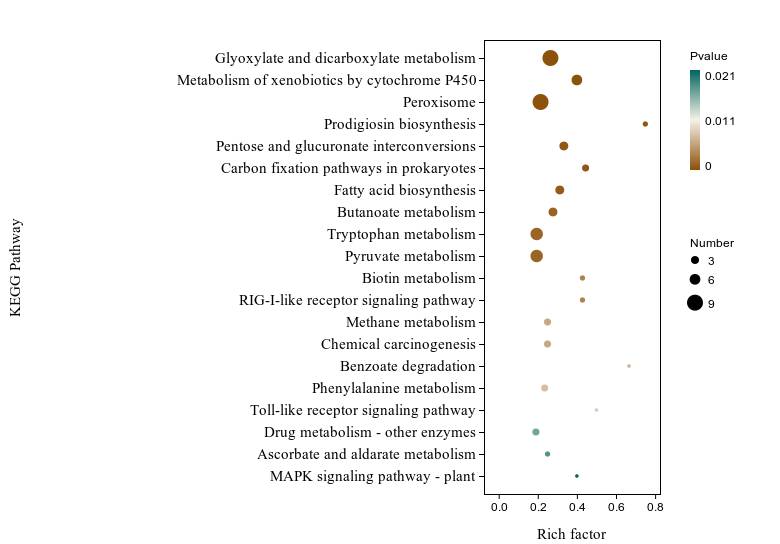


FIGURE S4

KEGG bubble plots for (A) XZJB vs. GLG, (B) DM vs. GLG, and (C) DM vs. XZJB.

TABLE S3 Annotations of the KEGG pathway for DEGs between XZ_JB and GLG.

| Pathway | Pathway ID | RichFactor | P-value | Up Genes | Down Genes |
| --- | --- | --- | --- | --- | --- |
| Prodigiosin biosynthesis | map00333 | 0.75 | 0.00019 |  | fabG |
| Retinol metabolism | map00830 | 0.33 | 0.00066 | CYP3A; RDH8 | frmA; CYP2C |
| Steroid hormone biosynthesis | map00140 | 0.31 | 0.00093 | CYP3A; CYP2D; CYP7B | CYP2C |
| Glycerolipid metabolism | map00561 | 0.23 | 0.00094 | AKR1A1 | lip; yahK; E3.2.1.22B; GCY1 |
| Biotin metabolism | map00780 | 0.43 | 0.0015 |  | fabG |
| Tyrosine metabolism | map00350 | 0.27 | 0.0017 | mfnA | IL4I1; FAHD1; frmA |
| Fatty acid biosynthesis | map00061 | 0.25 | 0.0022 | FASN | fabG |
| Glyoxylate and dicarboxylate metabolism | map00630 | 0.15 | 0.0071 |  | HOGA1; aceB; BNA7; MDH2; ICL |
| Carbon fixation in photosynthetic organisms | map00710 | 0.19 | 0.019 |  | MDH2; pckA |
| Chemical carcinogenesis | map05204 | 0.19 | 0.019 | CYP3A | CYP2C |
| Amino sugar and nucleotide sugar metabolism | map00520 | 0.11 | 0.0247 | E3.5.1.41; GME | XYL4; E2.7.1.46 |
| Linoleic acid metabolism | map00591 | 0.29 | 0.0248 | CYP3A | CYP2C |
| Thyroid hormone synthesis | map04918 | 0.29 | 0.0248 |  | ATF2; GSR |
| Glycolysis / Gluconeogenesis | map00010 | 0.11 | 0.0372 | AKR1A1 | FrmA; yahK; pckA |
| Pyruvate metabolism | map00620 | 0.11 | 0.041 | PC | aceB; MDH2; pckA |

TABLE S4 Annotations of the KEGG pathway for DEGs between DM and GLG.

| Pathway | Pathway ID | RichFactor | P-value | Up Genes | Down Genes |
| --- | --- | --- | --- | --- | --- |
| Glyoxylate and dicarboxylate metabolism | map00630 | 0.26 | 0.00317 | HOGA1; FDH; ACSS1_2; AAE7; AACT | katE; ACO; oxdD |
| Metabolism of xenobiotics by cytochrome P450 | map00980 | 0.4 | 0.0032 | GST; CYPF; EPHX1; DHDH | AKR7; UGT |
| Peroxisome | map04146 | 0.21 | 0.0065 | PIPOX; PROX5; IDH1; DECR2; ACSL | katE; MPV17 |
| Prodigiosin biosynthesis | map00333 | 0.75 | 0.015 |  | fabG |
| Pentose and glucuronate interconversions | map00040 | 0.33 | 0.015 | E3.2.1.15; DHDH; kduD | UGT; UGDH |
| Carbon fixation pathways in prokaryotes | map00720 | 0.44 | 0.015 | ACSS1_2; AACT; IDH1 | ACO |
| Fatty acid biosynthesis | map00061 | 0.31 | 0.016 | ACSL | fabG |
| Butanoate metabolism | map00650 | 0.28 | 0.022 | BDH; butA; paaH; AACT; AAE7 |  |
| Tryptophan metabolism | map00380 | 0.19 | 0.022 | ASMT; E3.5.1.4; AACT; IL4I1 | katE |
| Pyruvate metabolism | map00620 | 0.19 | 0.022 | ACSS1_2; AAE7; AACT; E1.13.12.4; maeB | yvgN; GLX3 |
| Biotin metabolism | map00780 | 0.43 | 0.046 |  | fabG |
| RIG-I-like receptor signaling pathway | map04622 | 0.43 | 0.046 | RIPK1; PIN1 | P38 |

TABLE S5 Annotations of the KEGG pathway for DEGs between DM and XZ_JB.

| Pathway | Pathway ID | RichFactor | P-value | Up Genes | Down Genes |
| --- | --- | --- | --- | --- | --- |
| Glyoxylate and dicarboxylate metabolism | map00630 | 0.26 | 0.00317 | HOGA1; FDH; ACSS1_2; AAE7; AACT | katE; ACO; oxdD |
| Metabolism of xenobiotics by cytochrome P450 | map00980 | 0.4 | 0.0032 | GST; CYPF; EPHX1; DHDH | AKR7; UGT |
| Peroxisome | map04146 | 0.21 | 0.0065 | PIPOX; PROX5; IDH1; DECR2; ACSL | katE; MPV17 |
| Prodigiosin biosynthesis | map00333 | 0.75 | 0.015 |  | fabG |
| Pentose and glucuronate interconversions | map00040 | 0.33 | 0.015 | E3.2.1.15; DHDH; kduD | UGT; UGDH |
| Carbon fixation pathways in prokaryotes | map00720 | 0.44 | 0.015 | ACSS1_2; AACT; IDH1 | ACO |
| Fatty acid biosynthesis | map00061 | 0.31 | 0.016 | ACSL | fabG |
| Butanoate metabolism | map00650 | 0.28 | 0.022 | BDH; butA; paaH; AACT; AAE7 |  |
| Tryptophan metabolism | map00380 | 0.19 | 0.022 | ASMT; E3.5.1.4; AACT; IL4I1 | katE |
| Pyruvate metabolism | map00620 | 0.19 | 0.022 | ACSS1_2; AAE7; AACT; E1.13.12.4; maeB | yvgN; GLX3 |
| Biotin metabolism | map00780 | 0.43 | 0.046 |  | fabG |
| RIG-I-like receptor signaling pathway | map04622 | 0.43 | 0.046 | RIPK1; PIN1 | P38 |

TABLE S6 Ergosterol biosynthesis genes of *T. gaoligongensis.*

| Gene | Function |
| --- | --- |
| SQS | squalene synthase |
| SES | Squalene Epoxidase |
| OSC | 2,3-oxidosqualene cyclase |
| Erg11 | lanosterol 14-alpha-demethylase |
| Erg24 | C-14 sterol reductase |
| Erg25 | C-4 sterol methyl oxidase |
| Erg26 | C-3 sterol dehydrogenase |
| Erg6 | sterol 24-C-methyl transferase |
| Erg2 | C-8 sterol isomerase |
| Erg3 | C-5 sterol desaturase |
| Erg4 | C-24(28) sterol reductase |
| Erg5 | C-22 sterol desaturase |

The gene names of *T. gaoligongensis* were assigned with reference to the *T. gaoligongensis* genome.：GenBank accession numberJAZIAZ000000000.(<https://www.ncbi.nlm.nih.gov/datasets/genome/GCA_037127245.1/>).


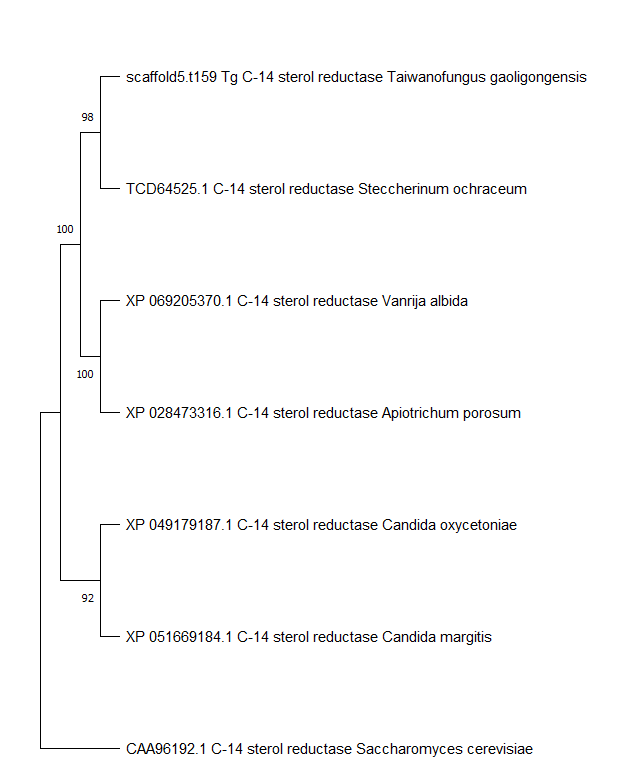


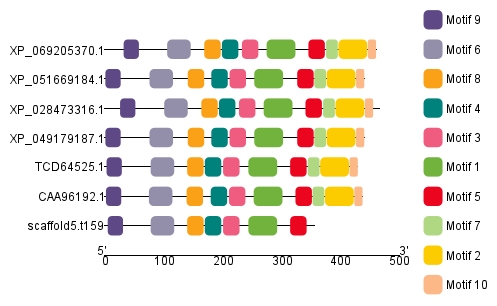


FIGURE S5

Phylogenetic tree of *TgErg24* C-14 sterol reductase with homologs from selected Basidiomycota, Ascomycota, and *S. cerevisiae*. The phylogenetic tree was constructed using the maximum likelihood method in MEGA11 with 1,000 bootstrap replicates under default parameters. Conserved motifs were predicted using MEME, and the results were visualized with TBtools (version 2.142).


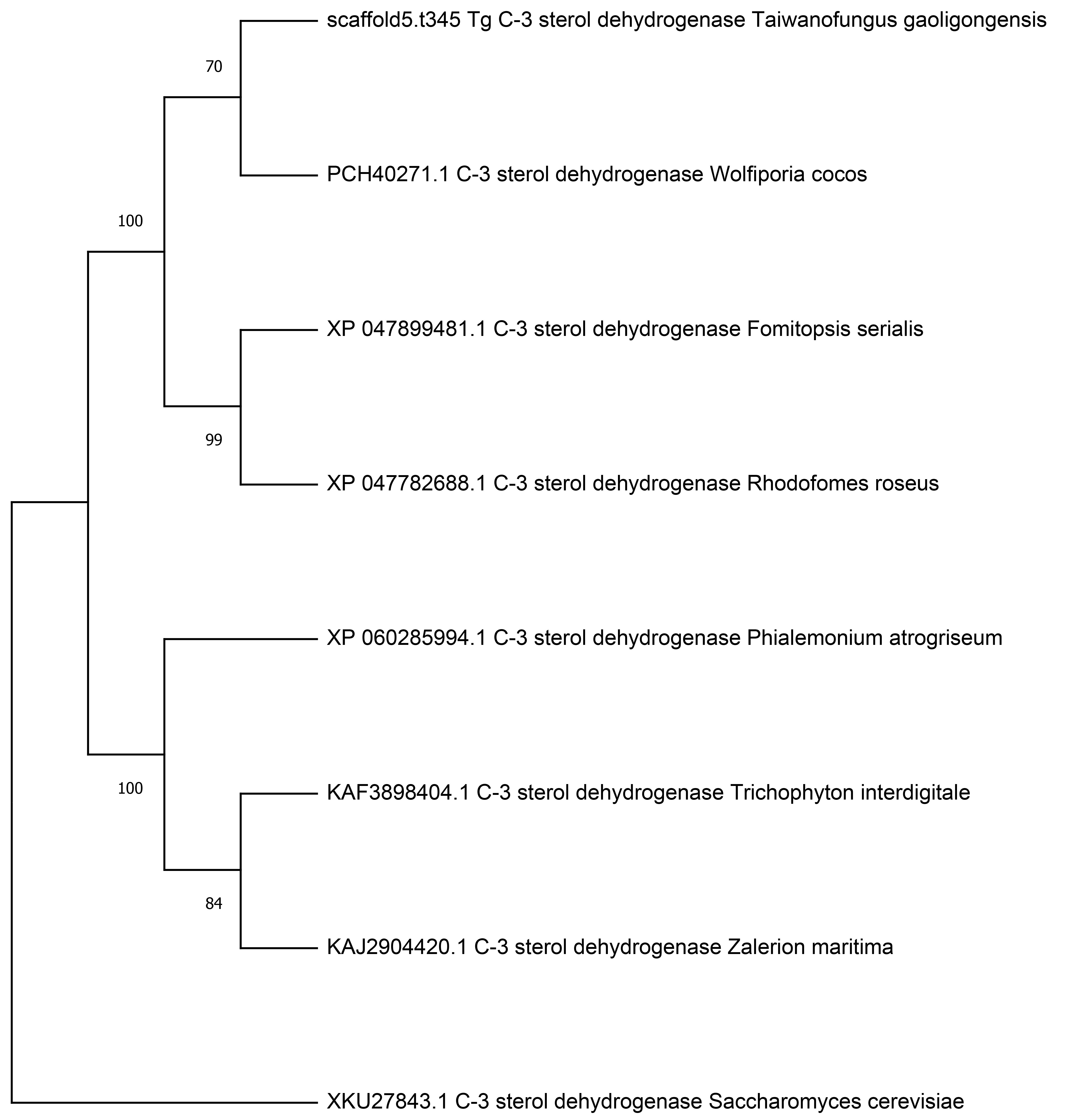


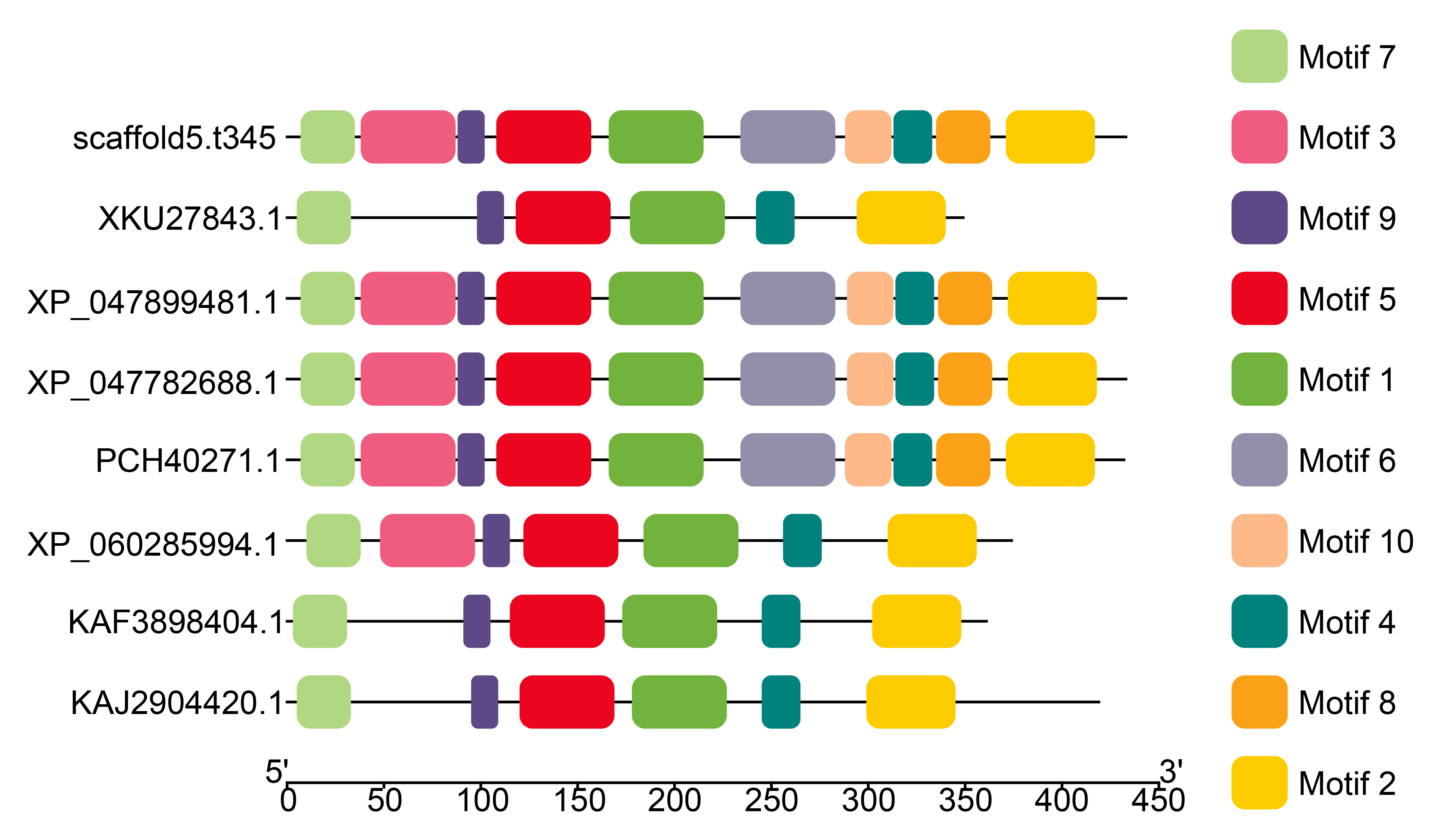


FIGURE S6

Phylogenetic tree of *TgErg26* C-3 sterol dehydrogenase with homologs from selected Basidiomycota, Ascomycota, and *S. cerevisiae*. The phylogenetic tree was constructed using the maximum likelihood method in MEGA11 with 1,000 bootstrap replicates under default parameters. Conserved motifs were predicted using MEME, and the results were visualized with TBtools (version 2.142).


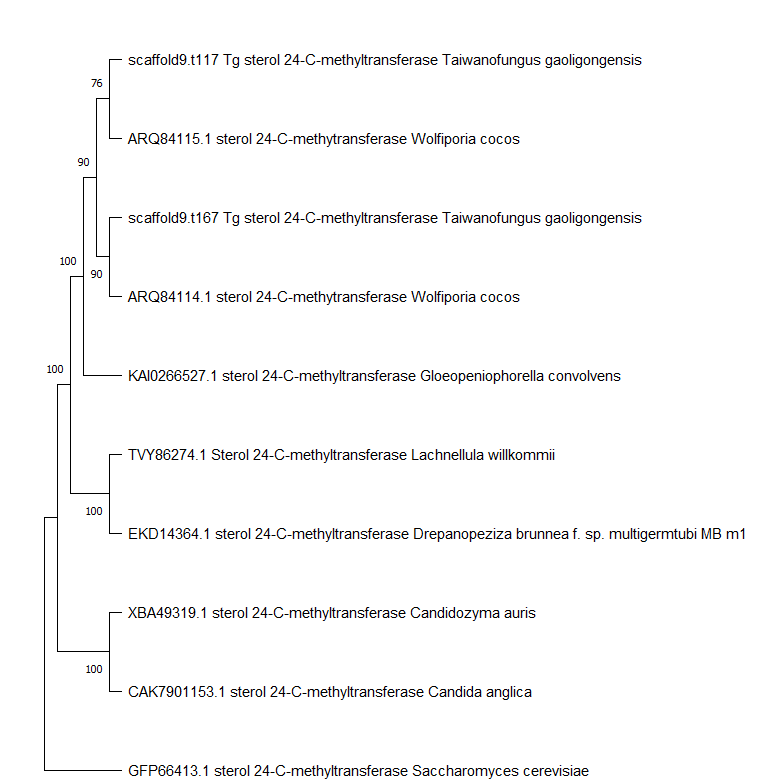


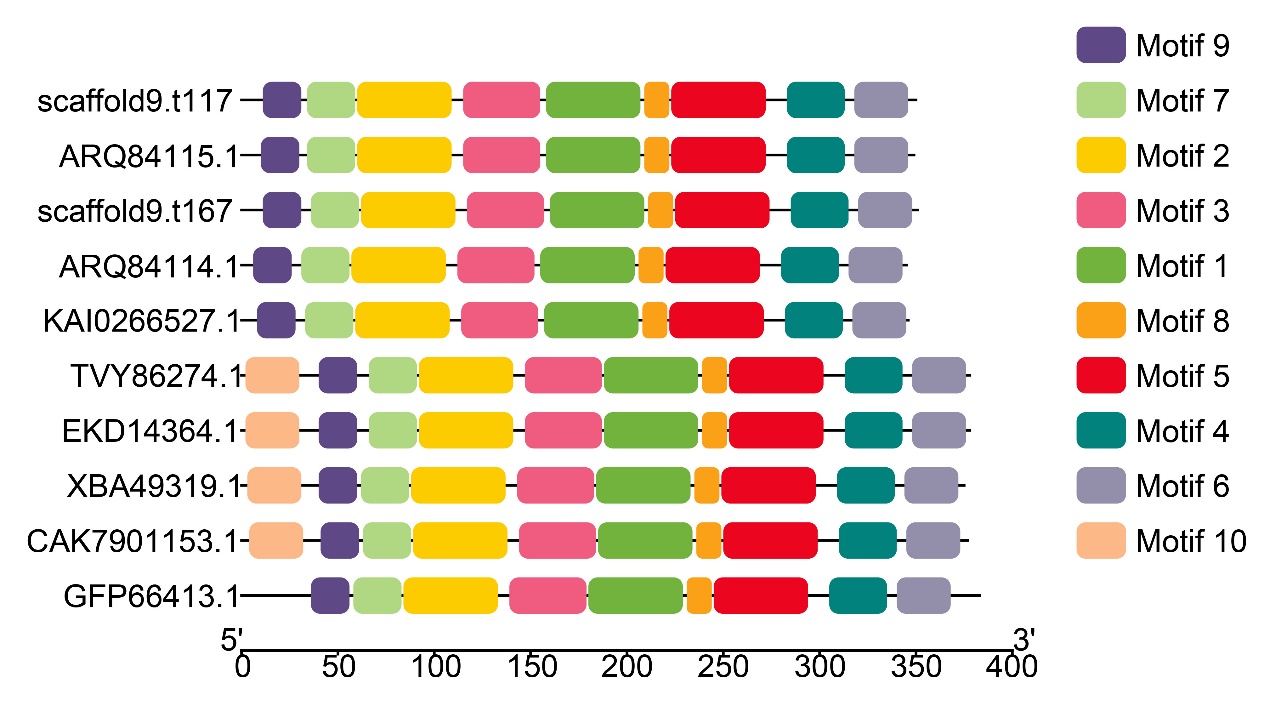


FIGURE S7

Phylogenetic tree of *TgErg6* sterol 24-C-methyl transferase with homologs from selected Basidiomycota, Ascomycota, and *S. cerevisiae*. The phylogenetic tree was constructed using the maximum likelihood method in MEGA11 with 1,000 bootstrap replicates under default parameters. Conserved motifs were predicted using MEME, and the results were visualized with TBtools (version 2.142).


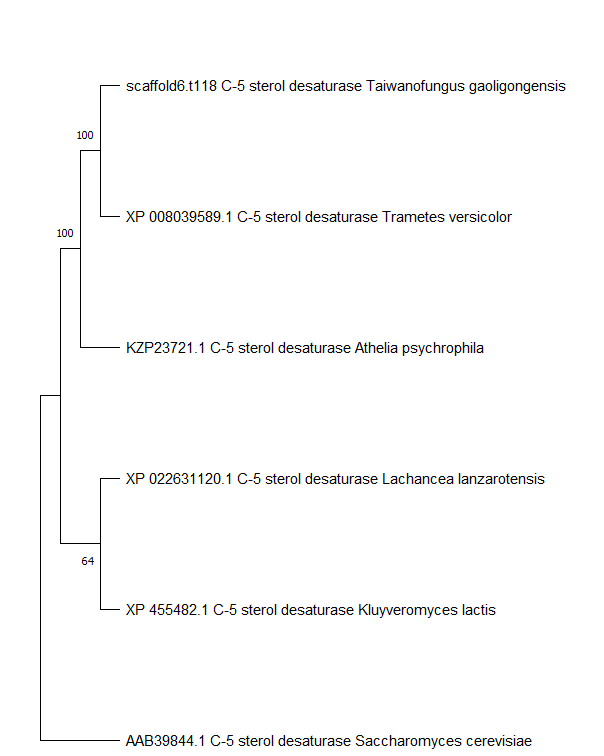


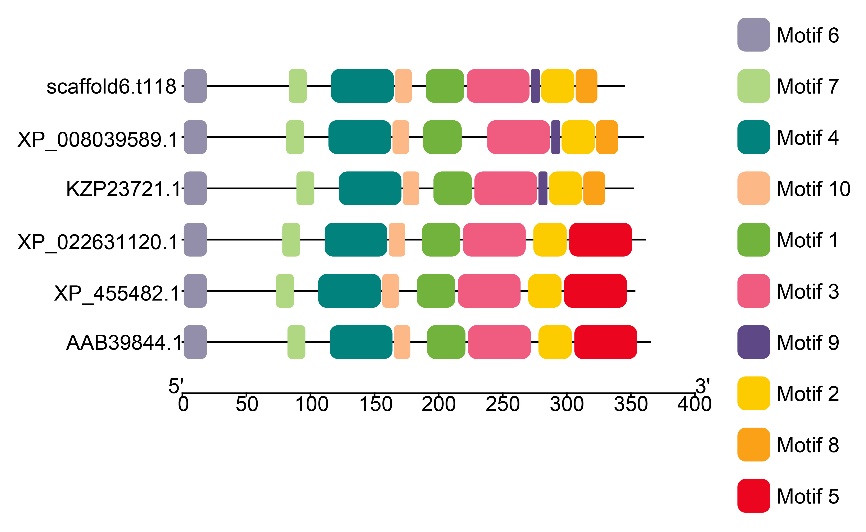


FIGURE S8

Phylogenetic tree of *TgErg3* C-5 sterol desaturase with homologs from selected Basidiomycota, Ascomycota, and *S. cerevisiae*. The phylogenetic tree was constructed using the maximum likelihood method in MEGA11 with 1,000 bootstrap replicates under default parameters. Conserved motifs were predicted using MEME, and the results were visualized with TBtools (version 2.142).


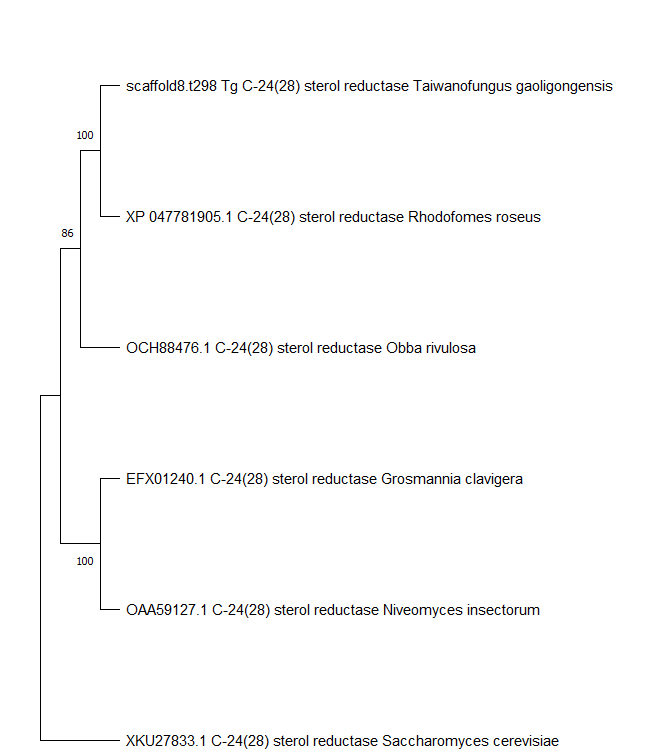


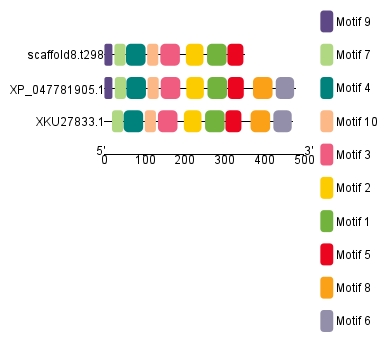


FIGURE S9

Phylogenetic tree of *TgErg4* C-24(28) sterol reductase with homologs from selected Basidiomycota, Ascomycota, and *S. cerevisiae*. The phylogenetic tree was constructed using the maximum likelihood method in MEGA11 with 1,000 bootstrap replicates under default parameters. Conserved motifs were predicted using MEME, and the results were visualized with TBtools (version 2.142).


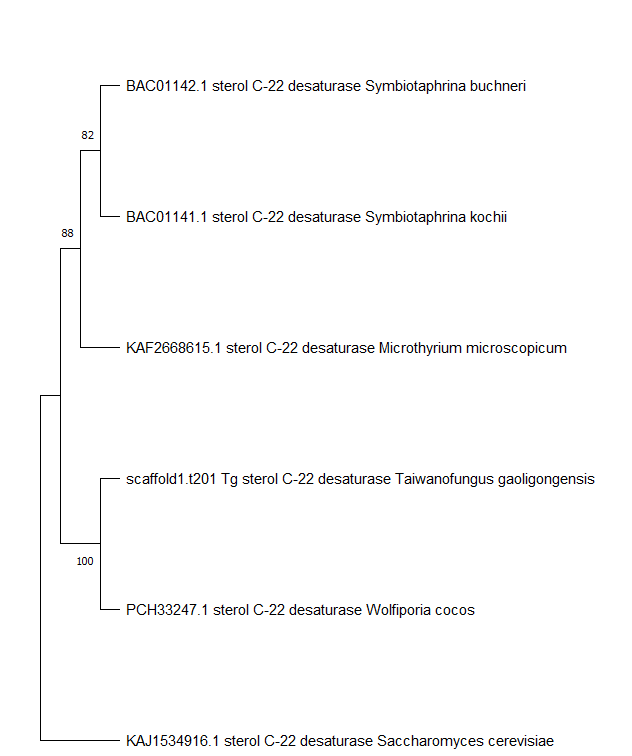


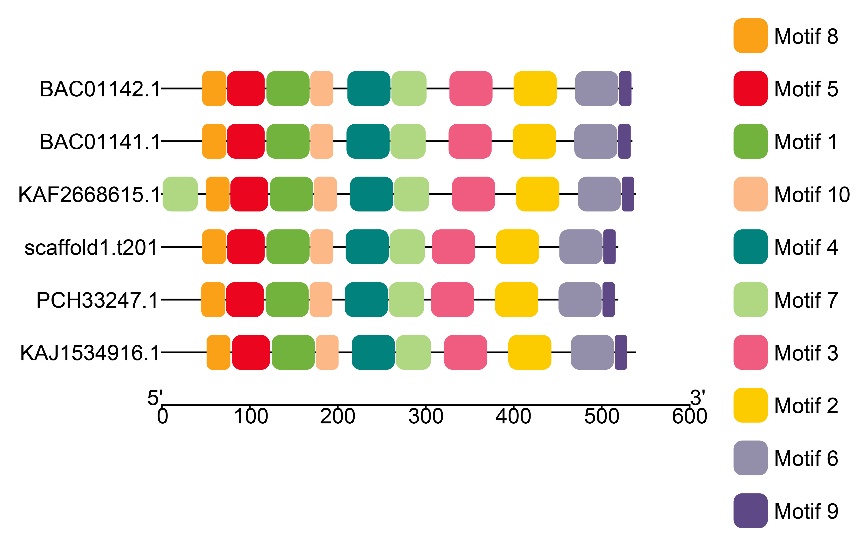


FIGURE S10

Phylogenetic tree of *TgErg5* C-22 sterol desaturase with homologs from selected Basidiomycota, Ascomycota, and *S. cerevisiae*. The phylogenetic tree was constructed using the maximum likelihood method in MEGA11 with 1,000 bootstrap replicates under default parameters. Conserved motifs were predicted using MEME, and the results were visualized with TBtools (version 2.142).

|  | Taiwanofungus camphoratus | Taiwanofungus gaoligongensis |  |  |  |  |  |
| --- | --- | --- | --- | --- | --- | --- | --- |
| Gene name | Accession | | Length | Identity | Similarity | Gaps | Score |
| SQS: squalene synthase | AHF22383.1 | scaffold8.t181 | 503 | 100% | 100% | 0% | 2435 |
| SES: squalene epoxidase | ALJ76841.1 | scaffold13.t22 | 481 | 99.2% | 100% | 0% | 2512 |
| OSC: 2,3-oxidosqualene cyclase | AIO10969.1 | scaffold8.t336 | 734 | 89.9% | 90.1% | 9.9% | 3580 |
| Erg11: lanosterol 14-alpha-demethylase | ABV66226.1 | scaffold6.t285 | 544 | 99.8% | 99.8% | 0% | 2856 |
| Erg25: C-4 sterol methyl oxidase | KAI0925313.1 | scaffold13.t245 | 330 | 99.4% | 99.4% | 0.6% | 1813.5 |
| Erg2: C-8 sterol isomerase | KAI0918405.1 | scaffold9.t270 | 207 | 95.7% | 97.1% | 0% | 1058 |

TABLE S7 The identity and similarity between ergosterol biosynthesis enzymes of *T. gaoligongensis* and the corresponding enzymes of *T. camphoratus* were analyzed using EMBOSS Water (<https://www.ebi.ac.uk/jdispatcher/psa/emboss_water>).


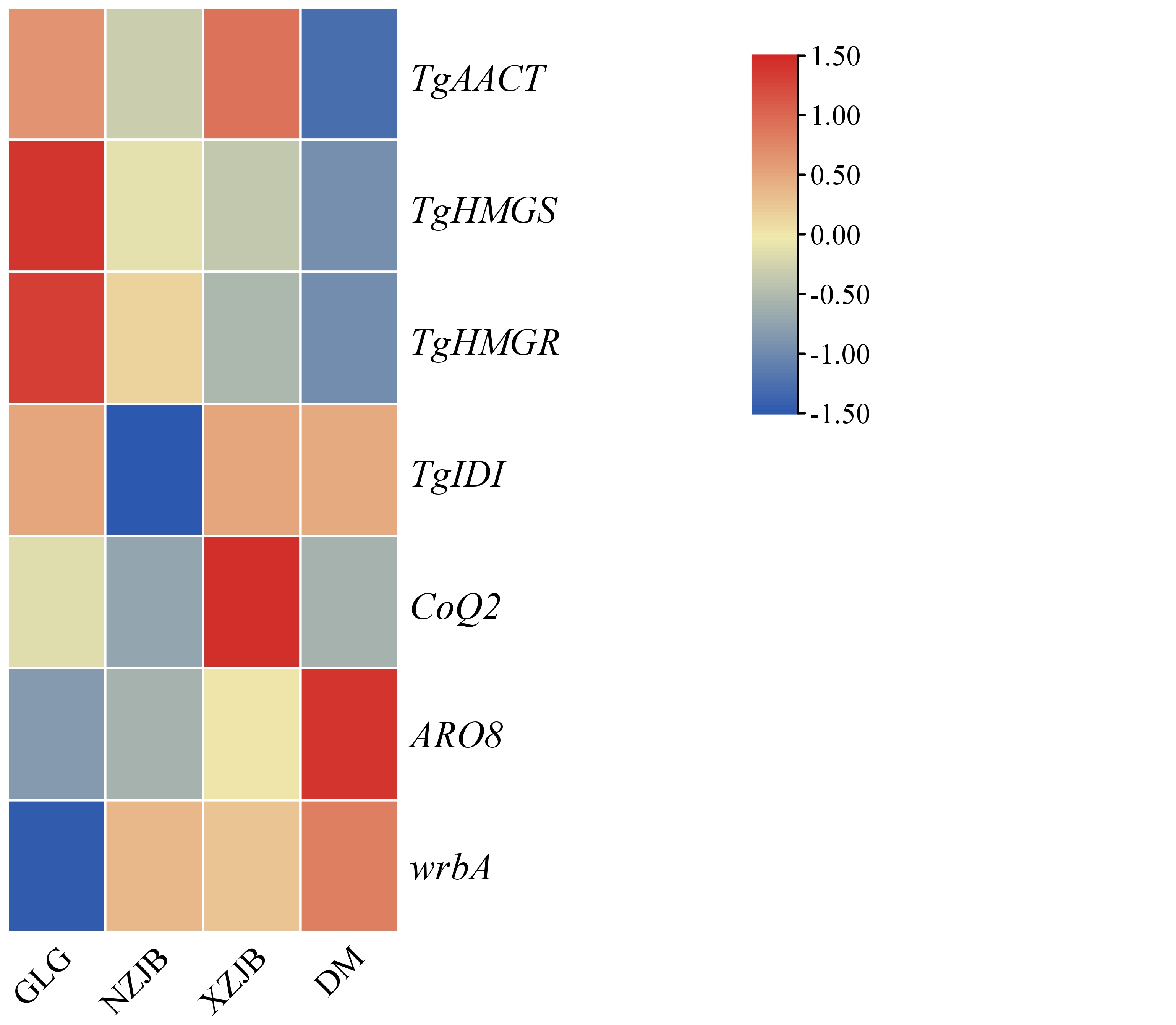


FIGURE S11

Heatmap of gene expression related to Antrodin C synthesis in various *T. gaoligongensis* samples. CoQ2: 4-hydroxybenzoate polyprenyltransferase; ARO8: aromatic amino acid aminotransferase I / 2-aminoadipate transaminase; wrbA: NAD(P)H dehydrogenase (quinone).


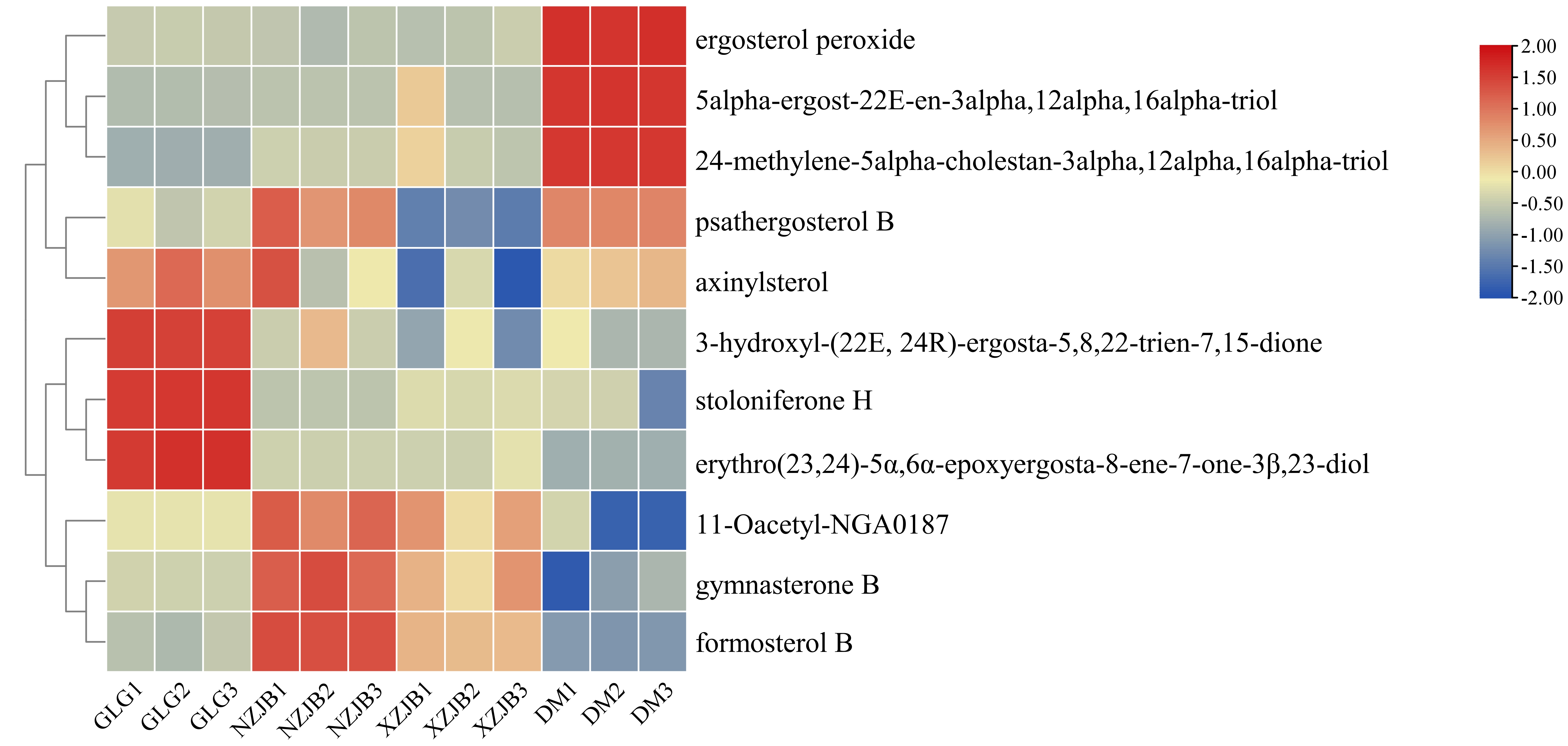


FIGURE S12

Differential levels of ergosterol derivatives in the metabolome of *T. gaoligongensis*.

TABLE S8 Primer list of genes for qRT-PCR.

| Primer Name | Sequence (5'-3') |
| --- | --- |
| actinF | ATGAGCAGGAGATGCGCA |
| actinR | TCGAGCACCACATGTTCT |
| *TgHMGRF* | GACTACGTCTTGGAGATTGT |
| *TgHMGRR* | CGCATTGACCGTGGCACGTG |
| *TgHSF4F* | CGAGTACACGGCTGATCTGT |
| *TgHSF4R* | TTACTCGGTAATAAGGTCTG |
| *TgErg6 1F* | AAGCCTGTACGAATTCGGAT |
| *TgErg6 1R* | ATCCGAATTCGTACAGGCTT |
| *TgHMG8F* | GTTACACCGGGCACGTATGT |
| *TgHMG8R* | TCTATCGACCCTGGAACATG |
| *TgHMGSF* | CGTATCGACGTTGGTACTGA |
| *TgHMGSR* | CGATCAACATTGCACAGGCA |
| *TgFPPS 1F* | GTAGAGTATGTCGACATCTA |
| *TgFPPS 1R* | CGTACGAATTCCACGCACCA |
| *TgSQSF* | TTGAACACGATCGACAACTG |
| *TgSQSR* | CTCTCGCCATGCACGTTACC |
| *TgOSCF* | TTGTGTGCATCCTATACCTT |
| *TgOSCR* | AGTCGTAAACATTGAGCACT |
| *TgErg11F* | CTTCGCTGACTTGGCATGCA |
| *TgErg11R* | AGAACCTCGTTCGGGACGTC |
| *TgErg25F* | CGCTGCATATGGCAAGTGCC |
| *TgErg25R* | TGCCTGGTGGGCTTGAAGAG |
| *TgErg26F* | CTTGTATACACGAGCTCTGC |
| *TgErg26R* | ATGCGTTACAATCGAGACGC |
| *TgErg6 2F* | GCTGCACTTGCTCGCCATGA |
| *TgErg6 2R* | CCTGGTGCGTGTACTTCGCT |

TABLE S9 The gene sequence of the synthetic genes.

| Gene name | Accession | Gene sequence |
| --- | --- | --- |
| *TgAACT* | scaffold3.g165 | ATGATCGCCACACGTTTCATAATCCCTTCTTTTGCTACTCGTCGTAAGCTAACGTCTCTTGTCCGTACAATGgtacgccaacgattccgttgaagaggttattcgctgacgagatttctagTCGACTCACGAAGTTGTCATCGTCGCTGCTTCACGGACACCTGTCGGTTCCTTGAATGGATCTTTGAAGGCATTGACGGCACCCCAGTTGGGTGTCATTGCCCTCAAACACGCCTTTGAGCAGTCCAAGGTTGACCCTGCTATTGTTGAGGAGATTTATTTTGGGAATGTCGTACAAGCTGGCGTCGGCCAGTCTCCCGCACGACAGGTCGCATTGGGTGCCGGTATGTCGCCCACCTCAGATGCGACCACGATCAATAAGGTATGCGCGAGCGGTATGAAGTCTGTCATGCTTGCCGCGCAAAGCATTTCATCCGGTTACAAAGGCGTTGTTGCTGCTGGAGGCATGGAAAGTATGAGTAACGCACCgtaagttgtgttagttttgtgcagagacaagtggactaacatagattacttgtctacctattagATTCCTCCTGCCGCGTCAGAATCCTGCCTTCGGTAAATTCGAGACGAAGGATTCTTTACAGAATGATGGTCTTTGGGATGTCTACAACGATTTTGCCATGGGCAATTGTGGAGAAGCTGCAGCTGAAAAGTTCCAGATCTCGCGAGAATCCCAGGATGCGCATGCTATCGAGTCTTTCAAGCGCGCGGAAAGGGCATGGCAATCAGGTGCTTTTAATTCGGAGGTTGCGCCTGTTACGATAAAGGGGAAGAAGGGTGACACCATTGTGAAGGAGGACGAGCAATATAAGCGCGTCATCTACGAAAAGGTTTCGACACTCAGTCCAGCTTTCAAAAAGGGCGGTACCATCACGCCTGCAAACTCTTCACCGCTGAACGATGGTGCATCTGCTCTGGTATTGATGTCGGCTGAGAAGGCTAAGGAGCTCGGCGTCAAGCCTCTAGCGAAAGTCATTTgtgggttacctccgctggtcaccgtgtgtccgcggtgctgatgaaattgtgtccgttagCCTATGCTGACGCAGGAACGGACCCGATCGACTTTCCCATCGCCCCTACCGTCGCCATTCCGAAGGCGCTGCAGAAGGCCAACCTTAAGGTAGAGGATATCACTCTATTCGAGATCAATGAGGCGTTCTCTGCTGTTGTCCGGATCGCTGAGAAGGTTCTGGGTATCGACCCGGCGAAGATTAACGTGAACGGgtatgtgcattcaacatctatctgactcatctccgtaccgatacgaccgttttccagTGGTGCGGTTGCTCTTGGGCATGCCATTGGAAACTCAGGGTCACGAATTATCGTATCGCTTGTTCATGCCCTGAAATCGGGCGAGTACGGTGCCGCTGGAGTATGCAATGGGgtatgtaccttattttctaggaacgcgattgaagattgaaaagtagctcttccagGGGGGGGCGGCTTCAGCGATCGTTATCCAGAAACTATAG |
| *TgHMGS* | scaffold8.g138 | ATGACCATCCCTCTCAACGGCTCTGCTCGCGTGCACAGCGACATTGAGGCGCCCCTGCGTCCCAAAGACGTCGGAATCCTTGCCATGGAGATGTATTTCCCTCGCAGGgtacgtgttgagcattacgccgcgcgtgaggggctttctctgaggctcatgacgaacagTGCATCTCCGAGGAGGAGCTCGAGGAGTTCGACGGTGTCGCAAAGGGCAAATACACCATCGGTCTTGGCCAGAAATTCATGGCCTGCTGTGATGACCGCGAAGACATCAACTCGTTCGCCTTGACCGgtgtgtattgttcaaacccaccggaatcgtttccaagtcactcacgtcatgttcagCCGTCGCCAACCTGCTCGAGAAGTACGACATCGATCCGAGGTCGATCGGCCGTATCGACGTTGGTACTGAGACCATCATCGACAAGTCTAAGGCGACGAAGACTGTCCTTATGGACTTGTTCGCCGACGCAGGCAACACAGATATCGAGGGCGTCGACTCGAAGAACGCCTGCTACGGCTCTACCGCGTCACTCTTCAACGCCATCAACTGGGTCGAGTCTTCGTCATGGGACGGCCGCAATGCTATTGTCGTTGCGGGCGACATTGCCATCTACGCCGAGGGTACCGGGCGGCCCACCGGTGGCGCTGGTGCCTGTGCAATGTTGATCGGCCCCAACGCGCCTCTGGCGTTTGAGCgtgagtacctcttcgaaacgtatcacatagctgcgggctgcaccgttccatctgtcgtcgttgctgctaaggcgggtctagcggagatggaaacccttcggcgtctttcactcttcggtgttttccatttagactgacttcctccgcttcgtaccatcgagttgtgctgactggtactcttgactcttctagCCATCCATGGCTCGCACATGGTTAACACCTACGACTTCTACAAGCCGAAGCTCGACTCCGAATACCCCGAAGTCGACGGCCCACTCTCTATCACCACCTACGTGTCCGCTATCGATGCATCATACACCGCCTTCCGCCGGAAACATGCCAAGGCGAAGAAGATCGCGGGCTTGAACGGCAACAGTGACGCATCGTCGCTTGCTGCATTCTCACTCGAAGATGTCGACTATCCTGTGTTCCACAGTCCATACGGCAAGATGGTCCAGAAGGCCCATGCCCGTCTCgtatgtctgccattcgttcctatgccgtgtgaaatgctcacgccttcgccttcagGTCTACAATGACTTCATGGCGAACCCCAAATCACCGAAATACACATCCGTCCCAAACCCCGAGACCATCCTCGCGCAGCCATACAAGGAGTCGCTGACGGATAAGACGCTTGAGAAGGCGTTCATGGCCGTCGCCAAGTCCGAGTTTGAGACGACCGTCGAGAAATCGATGAAGTGCGCGCGGCGCTGTGGCAACATGTACACTGCGTCGCTGTACGGCGGCCTCGCGTCGCTCCTCGCGAGCGTCGAGCCCGCGGAGCTGCGCGGAAAGCGCATCAGCATGTTCGCGTTTGGCAGCGGGCTCGCGAGCAGCTTCTTCACGATCCGCGTCAGGGGCGACACGACGGAGATCAAGGAGAAGATGGACCTCGTCCAGCGCCTCACGAGCATGCAGGTGGTGCCGTGCCAGGAATACGTCGATGCGCTGCATgttagtgtcctcgtgctcgttacatgtggatggggttgctgacagctccggctctgccccttgtagCTGCGCGAGAAGAACCACAACGCCGGCTCGTATACCCCTGAGGGCTCCCTCGACAACATCTGGCCGGGAGGGTTCTACCTTGAAAGCATCGATGGCAAGTACAGACGGAAGTACGGCCGTAAGCCCAAGGCATAG |
| *TgHMGR* | scaffold10.g254 | ATGCACGCGACCTTCCTGCGGCTCTTCTTCGCCTCGCGTGCGCTCGGGTCGAACTTCTGGCTCTCCACGGGCATCTTCTTGTCCTCCGTCATCGGCTTCCTCTTCACGCTCCCGCTCTGCCGGTACCTCCGCATCCCGCTCGACCCGATCGCGATGACCGAGGCGCTCCCCTTCCTCGTGTGCACCGTCGGCTTCGACAAGCCGCTGCGCCTCGCGCGGGCGGTGCTCGCGCACCCGCAGACGCTCCGCCCGCAGGAGGACGGGCGCATGAAGTCCGCGGGGGATGTCGTGCTCGATGCGCTCGATCGCGTCGGGAACGGTATCCTGAGGGACTACGTCTTGGAGATTGTGGTACTACTTGTCGGCATCCAGAGCAAGGTCGGCGGGCTCAAGGAGTTCTGTGCGGTTGCTGCGATGGCACTCACTATGGACTGTTTCATGCTGTTCACTCTCTATGCTGCGGAGCTCACAGTTATGGTAGAGgtacgtgtcccattttgccttctatgtcacgtgccacggtcaatgcgtcgggcgtgacatcggcgccgactttcgtcacgccagtcgcggcacatgaccatggctatcctcctgtgctccggatccactttttgctcctcgtgattgtgtacagaacaatgacttacacactcaattctctcagGTTCGCCGAGTCAAGCTAGTTCGCGCTATGTCTCGTTCCCGCTCTCCTTCGATTGTCACACCCTCCGGTAATGTCGTGCGCACTGCGCTCGCCAAGCCTACTAGCAAGCCTGATTTATCCGCTCATAAGACCTGGAGTGAGCGGCTATCGGCTGCTGTGCTCGGTGTTAAGGGCTCCCTTCTCCCTGACGCGCAGGGTGGCAAGGCTTATGTGGCGGATGAGAACCCGATGACGAGACTGAAATTACTTCTGgtgcgctccctctttcaagatgattgtaggttgcgaggtctgatggttttcttgggctgtacagCTCGCGTCCTTCCTGACAATGCACATCCTCAACTTCTGCACGACTCTCACTCCAGCGACGACGCAAGCTCGCCACCATAAGCATCCTGTGCGCATGAATGCGGACGTGGCTCCTCCCGTTCCCCGGGTCGACATCACCAGTTCTGCCATCAGCAGTGTGCTTGCGAATCTTGCGATTGCGGAGCATGTGTACTCCATGAGTAGTGACATCGAAGAGTCAGAGCTCTTCGTCAAGGTTGCGGCCCCAGTGTACGTCCGTGTTGTCCCCACCGCACCCCCTCCATCCTCGACATCGCATTCTGAGGCAATTGAGAACTTCATGTCGAGCTGGTCGCGCCTCGTCGGCGACCCGGTGCTCTCGAAGTGGATCGTGGTCATGCTTGCGCTCAGTGTTATGTTGAACGGCTACCTTCTGAAGGGCATTGCGGCTGGTTCTGGACTTGCGGCCGTCCGTGCGGTGCGAAGCCAGGGCGTGCGATTCCAGTCTCGCGTTCGCTCGAGAGTCCGCCCTGAGAAGGAGCAGGAGCAGAGTGAGGTTCACACGCCCATCGCGCCTACGGTCGTCATGTCTTCGGTCGCACCCGTTGAGGTCGCACCTGAGCCAGTACCTGTCAAGGAGCTTCCCGTGTACAGTTCTCCGATCAACCTTGAGAGTGTCGACCTCAAGCTGCAGGAGAAGCTTGCTCGTGCTGCGGCGCGTCCCTTGACGCCACCAAGCGATTCGGAAAGCTTGAAGATATCGCGCAGCGACTCGAGGCGCTCGCTCGAGGAGTGCATCGATATCTTCGAGAATGGACCTCGGCCTGTGTCTGTCTCGCTCTCGATGTTGAATGACGAAGAGGTCATTTTGCTCGCGCAGAACGGAAAGATTGCGCCGTATGCTCTGGAAAAGATGCTTGGTGATCTGGAGAGAGCCATATCCATCCGCCGTGCACTTATCTgtgagcacgctctgtcgatctgtgtggtttcttgctaatctgatatgtatcaaagCGCGTGCATCCAGGACCAAGACACTCGAGGACTCGGATGTACCAATGTCCGGATACGATTATTCCCGGGTAATGGGTGCATGCTGCGAGAACGTCATTGGATACATGCCATTACCTCTTGGCATTGCGGGTCCCCTCAAGATCGATGGTGATCTCTATCCCATCCCGATGGCCACTGCGGAAGGCACCCTCGTTGCTTCGACCTCGCGTGGTTGCAAGGCCCTCAACTCCGGCGGAGGAGTCACCACCGTTCTGACCTATGACGGCATGACACGCGGTCCTGCCATCGACTTCCCTTCCATCGTTCTCGCAGCCCAAGCCAAGGCATGGGTCGAGTCCCCCGAGGGCTACGCAATCGTCAAGGAAGCTTTCGAGTCGACGTCGCGCTTCGCAAAGCTGCAGAATATCAAGTGTGCAATGGCTGGGCGCACGTTGTTCGTACGGTTCGCTACGCGTACTGGCGACGCCATGGGCATGAACATGATCTCGAAGGCAACTGAGAAGGCGCTTGAGACGATGGCGAAGAAGTTCCCGGAGATGGTCGTTCTCGCACTTTCTGGCAACTACTGTACGGATAAGAAGCCCGCCGCGATCAATTGGATCGAGGGTCGCGGGAAGAGTATTGTTGCGGAAGCTGTCGTACCTGGCAAGGTGGTGAAGACTGTCCTTAAGACAACTGTTGAAGCACTGTGCAATCTGAACACGAAGAAGAATCTGGTTGGCAGCGCGATGGCCGGCTCTGTGGGAGGATTCAATGCACACGCGGCGAACATTTTGACCGCTGTTTTCTTAGCAACCGGTCAAGACCCTGCTCAAAATGTAGAGAGTTCTCAGTGCATCACGCTTATGGAGCCgtaagtgtgctattcaatcactatttccttcctacttatctctgatcatatgtagCACTAACAATGGCGAAGACTTACTCATGACTGTTTCCATGCCCTGTATCGAGGTTGGAACCGTTGGAGGTGGTACCGTCCTTGCACCTCAACAGGCCATCCTCGAAATGCTCGGTGTCAAGGGCGCGCATCCAACTAACCCCGGCGAGAATGCACAACGACTTGCACGTATCATCGCTTCTGCTGTCATGGCAGGCGAGCTGTCCCTCATTAGCGCACTTGCTGCGGGTCACCTGGTGCGGGCGCACATGGCTCACAATCGGTCGCAAGCGAACACAGAAGCAAACACCCCCAGTATGTCACGGCCAGTTACTCCCGGTCCCAATGGCCCTTCCGGTGGCGCATTCTGGGCTAACGACGCGAAGGGCCTGATGACACCTATGAGCATGTCTTCAGGGTCGCATACGCCTGCGCCACCTGGTTACGTAGTAGAAGCGAGGCCATGA |
| *TgPMK* | scaffold4.g356 | ATGACCACTGTCGTGTCTTCCCCCGGAAAGGTCCTCCTCGCAGGGGGGTACCTGGTGCTTGACCCCGCCTATCCGGGCGTCGTCGTCTCGACGAGCTCGCGATTCTTTACCGTCGTACAAGACCTTGACGAAGCCGCGACTATCGCGCGGGCACAGGCTGGCCGCCCGATTGAGATCCGCGTGCGATCGCCCCAGTTCGTAGATGCGACATGGGTCTATTTCGTCCACTTCGACTTTGACGGTGTCCGCGTGGAACAGGTCGCAGACAAgtaagctcgtttcactcgtcgtggagtgtactgagggggtttgcaccccagCTCCTCGACCTCGTCGACAAAAAACAAATTCGTGCACCTCGCTCTACAGCGCACCCTGTCGCTGGCCCTCGAGGCCAGGAGCGCAGGGGCACTACAAAATAGCTTGTCGTATGGCCTCGACATCACCATCGTCGGCGATAATGACTTCTACTCGCAGCGCGCACAGgtccgtatctgctcagtgtgcaaccgctcacgctccgtgctttacctccgcttcgacagCTTGCAGCCCGCAGCCTTCCTCCCACACTTGCCTCGCTCTCGCAGCTCCCGCCATTCAGCCATACGGGCGTCCGCCTGCCCGAGGTGCACAAGACCGGCCTCGGCTCCTCCGCGGCGCTCATCACCTCGCTCGTCTCCAGCCTCCTCCTCCACCTGAACGTCATCCCGCGCGACGCCTTCGCCAGCGACGCCCACGGCGGCACAGCGTCCGCGAGCGCGGGCCGCCGCCTCGCGCACAACCTCGCGCAGTACGTGCACTGCCTCGCGCAGGGCAAGGTCGGCAGCGGGTTCGACGTGTCCGCGGCGGTGTTTGGCAGCCAGCTCTACACCCGCTTCGACCCCGCGGTGCTGCAGCCGCTCATGAGCGACGATCCGgtaagttcgccctccggcgtcaggcccaggggttcttgctcactctcaggcggtgtgcacggtgatagGCTGCTGTCAGCCAACCGCTCCTTCCCATCATCTCTCCGTCGAACGCGGCGTGGAATCATCGCGTAGAACCATTCAAGCTTCCGCCACTGACGCGACTGATGCTCGCAGACGTCGACGCTGGAAGCGACACGCCCTCCCTTGTTGGCAAGGTCCTAAAATGGCGCCAGGAATCCAGCGAGATGGGTCAGCGTCTCCTCTCAGGTGCTGTGCTGAAAGACTCATCGTGTACTGCCCATCTAGCCAGCGCACTATGGAACGCGCTCGACACAGTCAACCAGGCGCTGAGCAAGACATTGCTCAGAATGTCCGAGCTGCACGCACGCGATCCTGCAGCTTACGCGAAAGCTGTCAAGTACTTGTCAACATTGCAGTCTGTACAGgtaagtggacagtgatcctgctcggcgcttttcggacacttgtcgagacaaaaatgtctattctcatgcatttccgctatagTGGCTTGCAAATCCGAACATATCGCAAGATGACCAGGAGATCATCGATGCGTTCACTGAGGCACACGGGCTATCTGAGgtccatttgtttctccactgtccactgccgtcgctaacactcatgcttcgcatcatcctgcagGACATCCGTGCAAAGATGCGCGAAATGGGCAATTTGTCCGGCGTGCCGATCGAGCCTCCCGAACAGACTGAACTGCTCGACGCGTGCATGTCTGGCGCGGGCGTTATTGGGGGTGGTGTTCCCGGAGgtgcgccgtctcccctgtgcctgccaatctcagtcccactcacctcagctcccacagCCGGAGGTTACGATGCGATCTGGCTGCTTGTCTTCGACCCGCTCGAATGCCCACCCGCGGAGCAGCCCTCGAGCCGCGTCGAGCGTGTCTGGGCAAATTGGCCAAGACTCGATGTCTCCCCACTGTCCGCATCGGAGAGCGCCGCGAAGGGCGTGCGGCTCGAAGACGTCGCTGCAGTACCGGGCCTGCAGGCGCTGGTCAACGCCCCCTGGTGA |
| *TgMVD* | scaffold4.g548 | ATGACTATCTATCAAGCGACGGCTTCCGCACCAGTGAATATCGCCTGCATCAAgtgagtgcattcaattggagatttgctaattctgactgtttatagGTACTGGGGGAAGCGGGACACAAAGCTCATCCTGCCCACGAACTCTTCTCTCTCCGTTACCCTTGACCAAGATCACCTTCGGTCGACGACCACATCCCGCGCTGATCCGTCATTCCAGAAGGATAGACTATGGTTGAATGGCGTCGAGGAAGATATCAAGGAAGGCGGTCGACTTGCGACGTGTATCAAGGAAATGAAGCGCTTGCGTCGGGAGCTTGAAGAGAAAGATTCCAACCTTCCTAAGgtgtgttattaacgaagcatattcctcatatattggcctacaacaccacgttttgcaagCTTTCTCCGTACGCTGTCCACATCTGCTCCCGAAACAACTTCCCGACTGCGGCAGGTCTTGCATCCTCTGCATCCGGTTTTGCAGCTCTCGTCGCATCGCTCGCTGCGCTCTACGCTCTCCCTGCATCTCCCTCTCAGCTGTCTCTTATTGCCCGTCAGGGCTCTGGGTCTGCATGCCGCTCCCTCTTCGGTGGCTTCGTCGCCTGGGAGATGGGCACGAAGCCGGACGGTTCGGACTCGCTCGCCGTTGAGGTTGCGCCGCGTGAGCACTGGCCAGACATCCACGCGCTTATTTGCGTCGTTTCCGACGACAAGAAGGGCACGTCCTCCACCTCGGGCATGCAACGCACCGTCGAGACATCCCCGCTTCTCCAGCACCGTATCGCACATGTCGTCCCCGCACGCATGAAGGCAATCTCGGAGGCGATCCATGCGCGTGATTTCGACACGTTCGCGCGCATTACGATGCAGGACTCGAACCAATTTCATGCCGTCGCGCTTGACACGGACCCGCCCATCTTCTACATGAACGACGTCTCACGCGCAATCATCGCTCTCATTGTCGAATACAACCGTGTATCTGTCGCGAACGGAGGTAAACTCAAGGCCGCTTATACCTACGATGCTGGTCCAAATGCCGTTATCTACGCACCAAAGGAGCACTTGAAAGAGATTATTGAGTTGATCGTCAATTACTTTCCCCAGGCCGAACCTTTCAAAGATCCCTTCTCACTGTTCGGTGCCGCAGGCGTACAGGGCAAGGTCGTGGATGGATTCAATACCGCGGTGGCGAAACCATTCAGCGTTGGCGCGGTGAAGGGCCTCATTCACACAAGAGTCGGAGACGGTCCGAGAGTATTGGGTACGGAGGAAGCATTGTTAGGTTCAGACGGGCTGCCAAAGGCGGCTTGA |
| *TgIDI* | scaffold1.g464 | ATGTCCTCCCCTGCCGTCGAAGCGTCTCGTTCCGTATTGTCCACCGTCGACTTGTCGAAATATGACGCTGAGCAGTCACGTTTAATGGACGAGAGGTGTATTCTAGTGGATGAACAGGACCGTCCTCTAGGGGCTGCTGACAAGAAAACTTgtgagtctaccatcgcgtgtatcttgtgttgtatttcggccaacggttcctagGCCATTTAATGGAAAACATCAATAAGGGACTTCTTCACCGCGCGTTCTCCGCTTTTGTTTTCCGGCCAGATGATGGAAAGCTCCTTCTGCAACAGCGCGCGACTGAGAAGATCACCTTCCCTGACATGTGGACCAACACCTGCTGCTCTCACCCATTAGATGACTTCGAAGAGGAAAAGGTAGAGGAGAACCAACTAGGTGTCAAAGTTGCGGCATCTCGGAAACTCGAACATGAACTGGGCATCCCCCAGAGCCAAAGCCCAGTCGATCAATTCCAATACCTCACTCGTATACATTACCTTGCGCCGTCCAATGGTCTCTGGGGTGAACATGAAAgtaagtcgattcgaactagatatcttgaatgacccactgacctgccgccctctaaagTCGACTACATCTTATTCTTGACAGCGAATGTCACTGTCAAGCCGAATTTGAACGAGATTCGTGACTACAAGTATGTAGACAAAGCCGAACTGCAAGCTATGTTCGAGGACCCTGgtacagtttctttactttacgggagaacgccttcctgaccatttttccttagCGAACTCTTTCACCCCTTGGTTCAAACTTATCGCTCGTGACTTCCTCTTTGGATGGTGGGACGAGCTACTCAATCGTAAAGGCAGTAACGGTAAAGTAGTTGCTCAGAGTCTTGCTGGACTAGTGGACGGTACCAGTGTAGTCAACATGGTCTAG |
| *TgFPPS 1* | scaffold13.g337 | ATGACCACGAAGGATGAACTCAAAGCCCTTAAGAGGCAGAAGTTCGAGGATGTGTTTCCGGTCATACGTGACGAGCTTCTCGCGTATACCACTGCTCAGGGCATGCCAAAGGATGCCATAGAATGGTACCGTAGAgtatgtcgacatctacatcggcaccatcgtatagtgctgaccataccaacagAACTTGGACTACAATGTCCCAGGTGGCAAGTTGAACCGCGGCCTGTCTGTGGTCGACACCGTCGAGATCCTCAAGGGTCGGACCTTGGAAGACGACGAGTACTTCAAAGCAGCCATCTTGGGATGGTGCGTGGAATTCgtacgttctacttcctattttgttccgtgattttgtgttgactatagttgtgttttgtagCTGCAAGCGTTCTTCCTTGTCTCAGACGACATGATGGACCAGTCTGTCACTCGTCGGGGGCAGCCATGCTATTTCAGGCTGGAGAGCGTCAACCACCTCGCCATCAATGACTCCTTCATGCTCGAGGCCGCTATCTATCACCTTCTCAAAACCCACTTCCGCTCTGAACCCTACTACGTCCACCTGCTCGAGCTCTTCCACGAAgtgagttaccgagtagaatttttgatcgcagatattcaaatgttgtcgttatcagACGACCTTCCAGACGGAGATGGGTCAACTGATCGATCTGATCACCGCTCCTGAAGACCATGTGGATTTAAGCAAATTTTCCTTGCAAAAgtgtgtgtgtgtggttgacttcatgacgagatgctgctcataccccatgttctccagACACTCACTCATTGTCATTTACAAGACCGCATACTACTCCTTCTATCTTCCTGTTGCTCTCGCCATGTACATGTGCGACATCGCACACTCTGCCACTCCTTCTACAGATCCCTATCAGCTAGCACAGTCAATCCTCATCCCCCTTGGCGAGTATTTTCAAGTGCAAGACGACTTCCTTGACTTTGCCGGTACGCCCGAACAAATCGGAAAGGTCGGCACAGACATCATCGACAACAAGTGCTCGTGGTGTATCAACACGGCCCTCGCCCTCGCGACTCCGGAACAGCGCGCCGTGCTTGATGCACACTATGGACGGAAGGACGCGGCTGCCGAAGCGCGCATCAAGGCGCTCTATGAAGAAATGGCCATTCGTAAGCACTATGCGGAATATGAGGAGCGAATGTATACACGTATCATGGGCCTCATCGAGAAGATTCCTGAGAAACCTAGTGCGACGGATGGCGCAGTGATGCTCAAGAGGGAGGTCTTCAAAAGCTTTTTGGATAAGATCTACAAGAGGCAAAAGTAA |
| *TgFPPS 2* | scaffold13.g339 | ATGACTCGTTCACTCTCGAGGCCGCTATCTATCACCTTCTCAAAACCCACTTCCGCTCTGAACCCCACTACGTCCATCTTCTCGAGATCTTCCACGAAgtgagttaccgtataaaattttcattcgcagacattcaaatgtagTTGCCATACAACCTTCCAGACGGAGATGGGTCAACTGCTCGACGCTCCTGAAGACCATGTCGATCTGAGCAAATTTTCCTTGCAGAAgtgcgtgtggttgaactcatgacgagatggtatttattctccagACACTTACTCATCGTCATCTACAAGACCGCATACTACTCCTTCCATCTCCCTGTAGCTTTCGCCATGTACATGTGCGGCATTGTGCACTCTGTCGCCCCTTCTGCAGATCCTTATCAGCTAGCACAATCAATTCTCATCCCCCTTGGCGAGTATTTCCAAGTGCAAGACGACTTCCTTAATTTTTCTGGTACGCCCGAACAAATCGGAAAGGTCGGCAGAGACATCATCGACAACAAGTGCTCGTGGTGCATCAACACGACCCTCGCCCTCGCAACTCTAGAACAGCGTGCCGTGCTCGATGCACACTATGGACGGAAGGACGCGGCTGCCGAAGCGCGCATCAAGGCGCTCTATGAAGAAATGGCCATTCGTAAGCACTATGCGGAATATGAGGAGCGAACGTATACACGTATCATGGGCCTCATCGAGAAGATTCCCGAGAAACCTAGCACGGCGGATAGTGCAGTGATGCTCAAGAGGGAGGTCTTCAAAAGCTTTTCGGATAAGATCTACAACAGGCAGAAATGA |
| *TgGPPS* | scaffold1.g861 | ATGCTCAATCTCTCAAATCTGCGAAGTCTGCTATCTCAGGTTCTCTCACTACCGGCGCTGCATACCGTGGTTCTATTCACGCCAGAGGGGCAACTTGTTTCGTTCGCTGCAGACGTATACAGGTCAAAAGACAATGTGCGGGTTGTCGTCGGTTTGAGCAGCGAGGTCTGGCAAGAGACCAAGGAACAAGGGATGGGTATGGTTGACAGTGAGgtgagtatggtccgtattgtggcacgtacaaaatgacgttaatctgaactctggcacagCTTGGCCGAGTTCTGGTACTTCCTGTCGAACCGGTCCGGAAGGTGCACGAAGAACGGGTTGATGAACCCCTCATGTTACTCGCGTTGAATGCCGAGGACTCTATTTCGTGGAGCGAGCTCGAGAACAAGgtaatttctcagcaatcatccatgaaattggttggacatccgttcacagGCAAGAGAACTAGCAAAGCATCTAGCAGAGCCGGTCTTGGAACTGCGTGGACGACTGTCTACAGGACCGATTCTGCTCATCAGCCCACGCGCCGAACgtacagcccgctagaggcgaatacgtaaactttttgccaaatagctaattggtatgccatctggaccatcttaccgtttgtagtgcactttgacagttcttccagactcggcggcagcgttgtcaacatgactttatgacagcctgggatgttccctcttataagtatgggttatctccaagagcaaataagacgcaaggctgttgaaccacaagattcgagggttagcgttccctaagggaccaagcagtgtccttaacgcgctgcatccagctcttgtgagccttaggttccaaaagtaacactagccagaaagcatgtgtctggtgcgatattcatagctttggtagtccgtagcgtatgtgagtatttaatcgtggcatgcgttcagcattggaaaataaagtaccggatatacgaaaggaagtgggttgttttttgagtgtatcgggaaaaatacccggtattccccaacgaacccgaccccgggcagcgtccgacgctactgtacagtggactgcgaccgtgacgactcgcgttcgcgtccaattatttacgcccggtccatcagctcaccttgacgtcctccaactcgcgatagtgtccttgctggtcatgagacgttggtctgtcgcaggagtgcacgccccatgtcatagtactattccaactgtcacgcggacttgccgaaaatacagtgggcatgcacaacagaagagaagttttcctgcacaaccaccagccatacggtctcatgaacagaccttctcagCACCTCCACCAAAGCCCCCCGTACGTTCCGACCCGTATTCATTGCTAGGGCCCCAGCTAGAGCGTCTTCGAGAAACGCTCCTCCACCTCTTGGGTTCGTCGCATCCCGGTCTCTCCGAAATTGCCAAATATTACTTCCTTCACCCCTCAAAACAGCTCAGGCCACTGCTGGTACTGTTATTCGCACAGGCCACTAATGGACTGGGCAATGGTTGGCATCTCAAACAGTGGGCAGCAGAGTGCGAGGGCGCAGGCGGGAGGGCGGAGGAGTTGGACCGACCCTTGACGCGTGCGGACGTGCTGAATGACTGGAATCCGAACATGCCAGACAACACTGCGTCTTTCGAGAGTCCATTCTCGCTACGGCCACCGCGTCCGCCTGTGCAACCACCCTTACCTCCACCATTTCCGCCTGCACACGACACGCTGACCCTCTCAATTCCACCTTGTCTCCTTCCCACACAAATACGCCTCGCGCAGATTGTGGAGATGATCCACGTTGCGTCTCTTTTACACGACGACGTGATTGACAAATCCCCTCTCCGTCGTGGTGTGCCATCGGCGCCCGCCGCGTTCGGGAACAAGCTCACCATTCTCGGAGGCGACTTTCTCCTCGGTCGTGCAAGTGCAACGCTCTCTCGTCTCGGCGACAACGAGGTCGTTGAGCTCATTGCCAGTGTGATCGCCAACCTCGTCGAGGGCGAGATCCTGCAGCTTAAATCTGTGCATGGCGAGGAGCTGGGTATTGCAGGTGCACCAACTTTGGGGAAAGACTACTTTAACATTTATTTGCAAAAGACGTATATGAAAACCGCGAGCTTAATGGCGAAAGGTGCACGAGCAGCGGTAGTATTAGGAGGTTGCAAGGAAGGGGAAGTCTGGAAGGAGGTAGCGTATGCGTACGGCCGTAATCTCGGCATTGCATTCCAGgtctgtctccaaattcccagttagttactggacgtcaagtgacttacctccatatactctagCTCGTAGACGATATCTTGGATTATGAAGCTGGGGAGGCTACCTTAGGTAAACCAGGTGGGGCTGATTTGCAGCTGGGGCTTGCGACCGGACCAGCTTTATTCGCATGGGAAGAGCACCCAGAAATGGGTCCCCTTATTAAGCGGAAGTTCGAGCGAGAAGGCGATGTTGAACTTGTAAGTGCACTTTCCGGCCCGTACCGTCGGCGTCACTGA |
| *TgSQS* | scaffold8.g181 | ATGGGCGCCCTTTCTATGCTCGTCTTGTTGCTCACGCATCCTCTCGAGTTCCGCACTCTTCTGCAGTACAAGCTCTGGCACGAATCGAATCGCGACATCACCGCGCCCAGCGAGCTCGAGAACTCGGGATGGAATCGCAAATCCATGCGCAGGTGCTGGGAATTGCTGGACATGACGAGCAGGAGTTTCGCGGGCGTCATCAAGGAGCTCGAGGGAGACTTGGCCAGAATCgtgagtgtcaccaactggtgcttttggcacggcccccctcggcctttcagcgggccagggctcttctaacctcgcgagccgtctgaaatgcatacacgagtattccaatgcttctgtcattcatttctagcattcgaatttgtgcgtactgttccggaaggaatcacttgggcatcggtctagcagttgtgatcgtgttgtgccaggttttgaacacgatcgacaactgagggccgaaatcaggctgtcgcaaacatgtgttggtcattcctcacgctattcacggcgggcggaatgtccaatgttccgctttaggtgttggcataccgtaactcctggtggaggatggtgtgtacatccgagtcgcggatatatttctgtacagaactcggtgctggagttgcaccactcctgatcaccgagttttcatgggtcaaagggtaacgtgcatggcgagagaacttaacaattctcggcaagctgcacagggacatctcgcagctactgtcagtgcgcgcgcgagcaaagatcattattccaacgatctcaaagcctccaaatagatcttggtcagctggaaaatgcacaagaatgtgtgttttgtgttttctggtagtttcatgctttgccatgtgataccttcacgaactccacctaccggtgctgggtggggtactttgggtgttgcccatcataccgtgtgtggcaccacaagaagagtccgactcgaagcccctgagttactggggcttgggtctgggatacgttcatcggtgtttcgtgacgggaagtcctgaatatcctacaggtgttgatggagggtacagggggcactagcttagggggactggcaagttttgcgtacgttcccttgacgccttccgcgtcctaatcagtttcgggatccccaggggagttggggtacacaggaacctcttaatgacatgaaatccttctactgcattgcatactataggttaccattcagaggtctagcctctcggaatgttatctgacattcgtttcctcatttcgcattatctgtgctcggaaggttcagcactggggacctgaccttctgtctctcagATATGCTTATTCTACTTGGTACTGCGAGGACTTGACACCATCGAAGACGACATGACACTGCCAGACGAAAAGAAACAACCTCTCCTGCGGTCCTTCCACGAACTGACCGTGACCCCTGGCTGGACATTCAACGGGAGTGGGCCATATGAGAAGGACCGGCAACTGCTAGTCGAGTATGCTGTCGTCAGCGAGGAGCTGAATCGCGTCGACACGAAgtgagcggtgcctgatttctaccccgtcgaagcgcactaattggtgtacagATACCTCGATGTGATCATCGACATCACACAGAAGATGGAGAACGGCATGGCGGACTACGCGCACCGTGCGGCGACGACAGGAGAGGTGTACGTCGAGAAGATCTCGGACTACGACCTGTACTGCCATTACGTCGCGGGGCTCGTGGGTGAGGGCCTGACGCGGCTGTGGTCGGCGTCGGGGAAGGAGGCACCATGGCTCGGCGAGCAGCTCGAGTTGGCAAACTCGATGGGCCTGATGCTGCAAAAGACGAACATCATCCGGGACTATCGCGAGGACGTCGAGGACCGGCGGTTCTTCTGGCCGCGCGAGATCTGGGGCCGAGAGGTCTACGGCGCGGCGTGCGGCCGGCCGGCATTCACCAAGATGGAGCAGATGTACCAGCCAGGGAGCGAGAAACAGGCGCTGTGGGTGCTCAGCGGGATGGTCGTCGATGTTCTTGGACACGCAGTGGACTCACTTGACTATTTACGCCTGCTCCGGCGGCAGAGTGTGTTCAACTTCTGCGCCATTCCCCAGACGATGGCGATGGCTACGCTTTGCCTTTGCTTCATGAATTACGAGATGTTCCAGCGGAATATCAAGATTCGCAAAGCAGAAGCGGCATCAgtgcgtatcagattaatttctcgtgcgcagtagagtgacataattttgaagCTCATTATGCGATCTACAAATCCACGAGATGTTGCGTACATCTTCCGTGATTATGCCCGCAAGATCCACAGTAAAGCTGTTCCAGAAGATCCCAGCTTCTTGCAAATCTCTGTTGCGTGTGGCAAGgtaagctcatgctcattcatgatgttgattgttgagtaccaaatgttcttctcttcgtcagATTGAACAATGGTGTGAACACCATTATCCGTCGTTCGTGAGCGTGTTGCATTCACCAGCGAGCGGGAACACGCAACAAGTATTCGACAAATCAGACGCCCGCACACGTATCATGGAGGCCTCTGAGAAACGCGATCGCGAGCTTCAATTGAGGAAACGCGCAAAGGAACTCTCTGCCAATGGCAAGATGAATGGAGTCAACAGCTCAGCACACCAGCTACAGGAACAGGGGCCTTCCACCAAAGAAATAATAATGTATATTGCGGCTGCATTCATTATCGTGTTCGCCGTCGGTCTGGGTGGTTTCTGGCTCCTCTTGAAGTATTTTGGATAG |
| *TgSES* | scaffold13.g22 | ATGGGGCACCCAGCAGAAATGTGGTCAACTAACTACGATGTCCTCATCGTCGGCGCCGGCATCGCAGGCTCGGCACTCGCTCACGCGCTCTCGTCCCTCAAGCGGACCAGCTCGAAGCCTCTGAGGATCTGCCTGCTCGAGCGTTCCCTGGCCGAGCCAGACCGCATTGTCGGCGAGCTGTTGCAGCCGGGCGGTATCATGGCGCTGCGGAAGCTGGGGATGGAGTCGTGTCTAGAGGACATCGACGCGGTGCCGGAGCACGGCTACTGCGTCGTGCTCGGCGGCCAGCCGGTGCACATCCCGTACCCCGGCGGACAGGAGGGGCGGAGCTTCCACCATGGGCGGTTTATCCAGAACCTGCGGGCGAAGGCCAAAGAGGCGCCAGGCGTGGACGTCGTCGAGGCGACGGTGTCGGAGCTGATCGAGTGTCCGGTGACGGGCCGCGTGCTCGGCGTGCGCGCGACGCGCAAGGAAAGTGCGGGCGCGAGCACGGTGGAGAAGGAGACCTTCTTTGCAGACCTGACGGTCATCGCGGACGGGTGCTTCTCCAACTTTCGGAGCACGGTGCTGGGCAAGGCGGGTGTGAAGCCGTCGACCAAAGGCCACTTTGTCGGCGTCGTGCTCGAGGATGTGAAGCTGCCAATCGACAACCACGGCACGGTTGTGCTCGTAAAGGGTCATGGGCCGGTGTTGTTATACCAAATCTCTGAGCATGACACGCGTATGCTGGTTGACGTCAAGAACCCCTTGCCTCCGGATCTCAAGgtgaccccttcgtctggtacccagaactctgtctcacgtttcttcgtgacagGACCTCATTCTTTCGGAAATTGTCCCGCAGCTACCGTCATCGTTGCATGTCCCCGTGCAGGAGGCACTGGAAAAAGACCGCTTAAGGCGGATGCCCAACTCGTTCCTACCATCCGCTGAGCAGGGTGGACAGCACACGAAGGAGGGCGTCTTCCTGCTCGGCGACTCGTGGAACATGCGACATCCGCTGACAGGTGGTGGGATGACCGTTGCATTCAATGACGTTGTGCTACTCCGAGATCTCATGGAAGGAGTCAACGACTTTGGGAACTGGAGAGAGATATCTGGTATCCTGCACCGCTGGCATTGGTTGCGTAAGCAGTATTCATCCACGATCAATGTCCTCAGTGTTGCGCTGTACGAATTATTCGGCGCAGAAGgtgggattgctttgtttgattagatacagcagatttcaggctgacaatagtctagACCCTCTACTCGAAGTATTGCGAACGGGCTGCTTCAAATATTTCGAGCTAGGTGGCAACTGCATCCGTGAACCAGTCTCCCTGCTCGCAGGgtaagcatttctcgttatgtcgtaatgcagcggacttatcacatcatactgcagGATTGAGCAGTCGCCTCTGCTACTGGCACGCCATTTCTTTGCAGTCGCACTGTACGCTATGTGGGTGATGTTCACGCACCCGCAGCGGGTGGGCTCTACGGAAAAACCCTTATACGCAGTGCCTCAACTATATGAATACCCGATGCTGTTCGTCAAGGCCATCCGAGTGgtaagggttcagttagactgcacttattacgtgctgataggaatacagCTCTGGACGGCATGTGTGGTTTTCCTTCCGCTGATATGGACGGAGATTCGGTGGTGgtgagctgatgctgatagtttgttacgttagGTCATTCATAATAGACTCCTTGCTCTCGTCGATGTCTGCTTTGTGA |
| *TgOSC* | scaffold8.g336 | ATGTCGTACTCCCCTCTCGACATCGCGGCCACCGGCCAGCATCCATTTACGGACTATGCGCGCTGGAGGCTCCGCGTTTCTGAAGATGGCCGCCACACTTGGCATTACCTGAACACCGACGAGGAATGTCGGGCGTGGCCACAAACAGACCTTGACAAATATTGGCTTGGTCTTCCTTTGAATCTCCCACCCCTGCCAGAGCCCGAGGATGCCCTTGCTGCCGCTCGTAATGGATACACTTTCTATAAGCGTCTCCAGGCTAAGGATGGGCATTGGCCTGGTGAATATGGCGGCCCCATGTTCTTGCTACCTGGTCTTGTCATTGGCTCCTATGTGACCGGGATGGGGTTCAAGATGGAGGAGAAACTGGAGATGATTCGGTACTTACTGAACCGTACTCACCCTGAGGATGGCGGTTGGGGAATgtacgtgttaaattgttgtgtgcatcctataccttatctttgtacagacatgttgaagggcattccaccgtcttcggaaccgcactcaattactgcgttatgcgcatcctcggtgtctctgctgaccatcctgctctcgtgaaggctagggctgttctacacaaacttggtggtgctactggagtccctgcttgggggaaattctggctctcagtgctcaatgtttacgactgggagggaaacaacccggtcacaccggaactctggcgagtcgcagtctccgcgcttatcgctatgatttcaaatgttctgaacgtgctaccagGTTGTTGCCCAATGCATTACCGTTCCATCCGCACAGATGGTGGATCCACACACGGACTGTCTATATCCCTATGTCCTACCTCTTTGGCATTCGTTATAAAATGGAGGAGAACGACCTAATTCTATCCCTGCGGCAGgtcagttcagcatctaagtatttcgtgctgactccttgctgactcagttctagGAATTATACCCTGAAAATTATTATCACATCGACTGGCCTGCACAGCGCAATAATGTCTGCAAAGCGGATCTATATGCACCTCATACCCGTATTTTTGACTTCCTTTACTCCCTTCTTGGTGTATATGAAAACTGTGCCCTACCACCTGCTCGACGTGCAGCTCTGGACTATTGCTATAAACTTATTGTCCAGGAGGATGAGAATACCGGATACCAAACGTTAGGGCCTGTGTCGAAGATGATGAACCTTATTGTCCGCGCACATGTCGACGGTCCAGAGAGTGATGCTTACAAGCTCCACATGGAGAAGCGGCAGGATTTCATGTGGGTCTGCACAGACGGCATGATGATGTGCGGCACCAACGGTAGCCAGCTATGGGATATTGGGTTTATTACACAGGCGCTGGTCGAGACTGGGCTTGCTCACGAGGAAGAGAACAAAGAAAGTATCGTCAATGCACTGCGCTGGCTGGATCAATGCCAGATCCAACAGAACCCGAAATACTATGAAAGTTCATTTAGACATCGTACGAAAGGTGCATGGCCTTTCAGTACCAAGACGCAGGGTTATACCGTCAGCGACTGCACTGGCGAGGGACTAAAGGCGGTATTGTACATTCAGGAACATGTTGAgtgagtattgcgtgtgtgcattgctccgaatcaatgtacttatttccgtctcagGTCGACTCCGAAGCTCGTGTCAGATCAGCGGATCTACGACTCCGTAGATCTCTTGCTGGGAATGCAGAACCATGATGGTGGATTTGCAAGTTACGAACTTATCAGAGGTCCGTGGTGGCTTGAAATGCTAAATCCAGCGGAAGTGTTCGgtgtgtttagatcgtcgcgacatcagctcatcactcacactgctgcagGCAAAATCATGATTGAACATAGCTATCCAGAGTGTACCACTTCCGTTATTACAGCGCTTTCTATCTTCCAGAAGCACTACCCGCAATATCGCCCAGCTGACATACGgtacgtcaatgtgcttgtttgctgcttacaaagtctgaatcgaccatatcagGCGGGTGGTTGAACGAGCGGTTCATTTTCTTCACAAGTCACAGATGCCTGAAGGTGGTTGGTTCGGCTCATGGGGAATTTGTTTCACATACGCCACCCAGTTTGCTTTAGAGAGTCTGTCCCTTGTAGGTGAGACCTATGAGACAAGTCCTTATGCCAGAAAGGCATGCAACTTCTTGATCTCAAAGCAGAGATCAGATGGTGGTTGGGGCGAGAGCTACAAGgtactgtgctcgactaaagggtgataagcattgttattaatgcgttctatctgcgccagTCGTGCGAGCTTACGGCCTGGGTGGAACACAAGCAGACACAGGTCGTGCAGACATGCTGGGCGGTTATGGCACTCATATACGCTCGTTACCCTGATCCTGAGCCTATCGAGCGTGCGGTGCGGTTGGTAATGTCACGTCAAAAACCAgtaagtccaatctgatcattcatatacgtcatctcgcttctaattacgcctctttgtaagGACGGCTCGTGGCCGCAGGAGGCTATGGAAGGCGTCTTTAACAAGAACGTCACCATAGCCTATCCGAACTTTAAGTTTTCCTTCACAATTTGGATGCTGGGACGTGCTCATCGGTATTTGGCGGAGCTGAAGGCTAGCCAAACTTCAAACGAAGCCCGTCAGGTTTAA |
| *TgErg11* | scaffold6.g285 | ATGTCGCTGAACATGAACGTCAGCTCTTCGGAGCCAAGTTGGGCGGCCCAAGCTCATGCTCAACTTGCTTCTCCATCTAGACTGATCTTGCTCTTCGTCGTCAACATACCCCTCATCGCCGTGGTCTTCAATGTCTTATACCAATTAGTGgtaaggcttcgagcctgggcgtcgtggcactcgctgaccttccttcccaaaaaaacgcagATGCCCCGTGACCGCACGGTGCCTCCCGTGGTGTTCCATCTCATCCCGTGGTTCGGATCGGCGGCTGCCTACGGTGGTGATCCCGTCGAGTTCTTCAAGTCATGCAGAGAAAAGgtaatcagcccgtcgtttgttgagaacttgtgacagcttcgctgacttggcatgcagTACGGCAACGTGTTCACGTTCATCCTTCTTGGGAAACGTGTTACCGTGGCGCTCGGTCCCCAAGGAAATGACTTTGTATTCGGTGGCAAGCACACGGTCATCGCGGCGGAAGATGTCTATCAGgtgggtgtcatgagctgtttccgtacgatatgccatgatctgagacacaccatctagCACTTGACGACACCTGTGTTCGGAAAGGACGTTGTGTACGACGTCCCGAACGAGGTTCTGATGGAGCAAAAAAAGTTCGTAAAGGTGTCACTGACAGTGGAAAAGTTCCGGGAGTACGTGGGGATGATCGAGGAGGAAATGACAGAGTTCATGAAGGGGGACCCAGCGTTCCTGATCTGGCAGATGAAGGACATCAACGAGTGGGGCACGTTCGATGCGTACAAGACACTGGCGGAGATGACAATCCTGACGGCGGCGCGGACGCTGCTGGGCAGGGAGGTGCGCGAGAGCTTGGACAAGGGCTTTGCGGACCTGTACTCGGACCTGGACCACGGAATGACACCGCTGCACTGGATGTTCTCGGACCTGCCTCTGCCAAGCTACCGGAAGCGGGACGCTGCGCACCAGAAGATCAGTGGGTTTTACCAGAGCATTTTGCAGAAGCGCAAGAGGCAGGAGAGTGAGgtgggtggtctaagctgttgtcggccactgtatgctcggactgacgttgatgttgggtctcagTATGAAGACGACGTGATGGGTTCTCTGATGAAACAGCAATACAGGGATGGTCGTGCGCTGCAGGACCATGAGATCGCACACATACTGATTGCATTGCTGATGGCTGGGCAACATACGAGCTCTTCATCGACGTCTTGGGCGCTGTTGCACTTGGCGGACCGTCCCGACGTCGCgtgagtattttgctggtatacgagcggggtaaatcatacgctaacatctgtctcccgccagCGACGCGCTGTACCAAGAACAAGTGGAGCATTTTGGCACTCCCGATGGCGGCCTGCGGGACATGACGTACGAAGAGATGCGGAAACTGCCCATCCTAGACGCGGTCATCCGTGAGACGCTCCGGATGCACACGCCCATCCACAGCATTATGCGGCAAGTGCGCAGCGACATCACGGTGCCCCCGACGCTTGGTGCGCCATCGGAAGACAATGTATACGTCGTGCCGAAGGGCCACATTGTTCTGGCATCGCCGGCGCTCAGCCAGATGGATCCGAAGATCTGGAAGGATGCAGACAAGTGGGACCCGTCGCGGTGGTATGACGCCGACGGCTTCGCGGCACAGGCGCACAGGCAGTACGACGAAGATGGCAAAGTCGACTTCATCTTCAGTAAAGGCACGGGGAGCCCTTACTTGCCGTTCGGGGCTGGCCGTCATCGGTGCATTGGCGAGCAGgtatgtcgtacttgtggtgttggcggcgtgctcgcttagcgacttgtgcatccagTTTGCGACTTTGCAGATCGGCGTCATCATTTCGACTTTTGTGCGCAAGATTGAGATGCGTCTGGACCAGCCTTTCCCTAAGCCTGATTACGCAgtacgtatcatcagaattaaaggattaacggcgacattctgatcgctttcaaaatagTCTATGATGGTCATACCATTACGCCCCTGCCAAATATTCTATCGCCGCAGGAAGTTTGACTAG |
| *TgErg24* | scaffold5.g159 | ATGTCGTCGCCAGAGACTACCCTCAATCCTCGCACAAAAGGGTATGAATTTTTCGGGCCACCAGGTGCCCTTTTTGTGACTTTGTCTGTCCCAATCATGTCCTATGCGCTTTACTTCGGGTGTTCAGAAGAGACGGGTGGCTGTCCGCCTCCCATTTCCATGCCCGCTATTATTGATGCCTTGACCAGCTGGGACTGGTGGAAGGGCCTTTGGGACACTCAGGCGACGCTCATGTATCTCGGCTGGTACTCATTCTGTGTCGCTGCTTGGTATGTCCTTCCAGGAGATTGGATCGAGGGTGTGCCATTGAGAACTGGAGGAAAGAAGAAATACAAAATCAATGgtacgggtgttgtgttcgtattctcccgtcggaaatgatgtcactcaattttgacagCATTCTCGACGTTTTTGCTCACCTTGGGCCTTACCTCGGGATACATCTGGCGTTACGGAGCGCAGTCTTTCACATTCATTTACGAAAAGTGGGTTGGCTTGGTGACCGCGTCCATCCTGATGTCGGTGGTGCAGGGTTTTGCATGTTACGCTGCGTCTTTCCGTGAAGGGGCGCTCCTCGCGTTAGGCGGCAATACTGGAAGTTTCATCTATGATgtatgtcacttcctaatttcaccgtcctgggcgctgacgatgtggggtcggcggagtagTTCTTCATCGGTAGAGAGTTGAACCCCTCAATAGGCTCTTTTGACGTCAAGTCCTTCAATGAGCTCCGCCCTGGGCTCATTCTATGGGCCGTCATTGACATCAGCATGGTGTGCGAGCAGGCCGTCCGTCGCGGCGGCAGCGTCACCGACTCGATGTGGTCGGTGTCCCTCTTCCAGCTCTGGTATGTTGCCGATGCTCTGTACAATGAAgtacgtatgttgtcctacccagagcacgtgatctgacgttcctgccgctagCCAGCCATCTTAACCACCATGGATATTACGACCGACGGCTTTGGGTTTATGCTTGCCGTTGGAGATTTGGTTTGGGTACCCTTTGTGTACTCTCTGCAGGCGCGATACCTGGTCTTCAAGCAGCTGGAACTCGGGCCTACGTTAACCGCTGTTGTATGTGGAGTGAACCTGCTCGGTTATTACATCTTCCGCAGCGCGAACGGTGAGAAGAACAACTTCCGGAACGGTCATAACCCGAAGAGTAAGCCTGCGCATACTGCCTCATCTTTTGGCGTGCTAATGACCGATGTATGTAAGACCTAA |
| *TgErg25* | scaffold13.g245 | ATGAACTCGACGGCTCCCGTATACGAATCTGCTGAGGTGTTATACGCCAACACCGACTTCAGTAAGCTAAACTGGGCGGAGCAGCAGTGGGCTGCCTGGTACCTATGGATCGGTAATCCCGTCATCGCAACTGGTCTCATGAGCTTCCTTCTCCATGAGgtgtgtgtggtccgaccatgatgttgaggtgtggcgcttcaatatagtctcccgcagTTTGTCTACTTTGGCCGTTGCATTCCCTGGATTATCATCGATGCTATTCCTTACTTCCGCCAGTGGAAGCTCCAGCCCAACAAGATCCCAACCCCACAGGAGCAATGGGAATGCACCAAACAGGTACTCTACTCTCATTTCACAATCGAGCTTCCCATGgtgcgttgtttcttccgctgcatatggcaagtgcccacagataggcgttcagATTTGGTTCTTCCACCCTATGGCCGAGATGTTCAACATGTCTACATGGCAAGTTCCCTTCCCTTCGTGGAAGCTCATGGCACCCCAGGTTGCGTCGTTCTTCGTTTTTGAAGACATGTTTCACTACTTCGgtgcgccgatcactgttactccttgattgttcttacctttgttctcttcaagCCCACCAGGCACTCCATTGGGGCCCTCTGTACAAGCACATCCACAAGCTTCATCATAAGTACTCCGCGCCTTTTGGTCTTGCGGCTGAATTCGCCCACCCTGCTGAGGTGATGATCCTTGGTACTGGGACGATTGCTGGTCCTCTCCTTTACTGCTACTTCCGTCGTGACCTGCACATTTTCACAATGTACATCTGGATCACCCTCCGTCTGTTCCAAGCTGTTGACGCACACAGTGgttatggcaagtttcttgctctccacttgattatttatatgctgatcatcacttctttatacagACTTCCCGTGGTCGCTGCAGCACATCGTTCCTTTCTGGTCCGGAGCTGAGCATCACGACTTCCACCACATGGCGTTTGTCAACAACTTCTCAACATCTTTCCGCTGGTGTGACCGTCTCTTTGGCACGGATGACAAGTATCGCCAGTACCGTGAGCGCATGCGGGCCATGAAGAAAGCTAACATGTCAAAGGAGGAGTTTGCCGCGATGGAGAGGAAGATGCTGGTCGAGATTGAGGCCGAGGGTGTGAAGGCGGAGGCCCAGACCGAGTCTTACAAGTATGGCAAGGCAAAGACTGCTTGA |
| *TgErg26* | scaffold5.g345 | ATGTCTGCACTGATCCGCGACGTCTACCTAGTAATCGGTGGAAGTGGCTTTCTTGGAAGGCATATCGTTGAGGCTTTGGTGACTCGCGGCGATACCGTATCGGTATTCGACATAGTACAGAGATATCACGATGTCCCCTTCTACTCAGGCGACATCAGTGAAGAGGGACAGGTGGGGGAAGCACTACAGAGGgtaatcatcactccgcttgtgttctacgttttgttagtactgatatttttcaccagAGTGGTGCAACCTGTATCATACACACCGCATCCCCACCGCACGGCATGGATGACCCGGCTCTCTATTGGAAAGTTAATGTTGATGGTACCAAGGCCGTGATAGCTGCCGCCGTCGCTAACGGTGTTCCAAAGCTTGTATACACGAGCTCTGCGGGTGTCGTATTCAATGGAGAGAGTCTTATCGACATTGACGAACGTCTTCAGCCTCCTGCGAAAGCTATGGACGCCTACAATGAGTCGAAGGCAAAGGCAGAAGAAATGGTCCTTGCCGCCAATGGGAAGGGCGGCCTCTACACCGTTGCCTTGCGCCCCGCAGGTCTGTTTGGgtgagcgcgtctcgattgtaacgcatagcacgaattgacggaccgctagTCCAGGAGACAGACAGATGATGACAGGTCTGTATGAGGTCTATGAGCGCAATCAGACCCATTTTCAGGTTGGAGACAACACCAATCTCTTCGACTGGACCTATATCACAAATGCCGCCCATGCGCACCTCCTTGCTGCTGATCGTCTTATACCCATGTCACCTGACCAGGCCGAAACAGTCAAGCAGGAGCTCAAATACGCCCTTCCTTATATTAGCTGCACTACCGGCAGCAAACGCTTGCCTACCTCCGAGTCTCGCCCTCTCGGGCCATATGTAACCCCACCGCCAAACGCGGAGCAGATCACCGCAAACTGGGACAACCCGCACCACCAGTCCGCCCAGCGACCAGCCATACGGGGGCGGTTTGACCCTCTTTCCGAGACTGCCTTAGAGCGCGAGGATGATGCGTCGCTGCAGGTCGCCGGGCAAGTATTCTTCATCACGAACGGAGAACCGTTGTACTTCTGGGACGTGCCGCGCGTTGCGTGGCGGTTCTTCGACGAGCACTTCGCGACCAACAAGACACAGCGCCGGACATTCCACCTATCAAAGGAAGTTGGAATGGTACTTGCGAGCGCGGCGGAGTGGTGGGCATGGCTGGTTGGCAAGCACCCTGGATTCACGCGCTTCAGGGTGACATTTAGCTGTGTATGGAGATGCCATAACATAGAGAGGGCGCGAAGGGTGTTGGGGTACGAACCGCAGGTTGGCATGGAGGAGGGTTTGAGGAAAACGCTTGAGgtatgtgaccaattttacctctgtaaatggatgtcttaccattatcacagTGGTTCGTTGCGGATCAGAAAAAATTTGCAAAAGCGTGA |
| *TgErg6 1* | scaffold9.g167 | ATGTCGCCCGTTGCTACTAAGGCCGACGCTGACGGCCGTGTCGGGTCGCGCATCCAAAACTATACTGGTTTTTGGCAAAAGGACATCAGCAAGGATGGCAAGGCTGACACCGACAACCGCGTAGAGAACTACACAGACGTAATAAACGgtaaggtcgatcacatgtgtaaagtatgcgtcctgagaatgatctacgtgcagGGTACTACGACGGCGCAACAAGCCTGTACGAATTCGGATGGGCGAGGTCGTTCCATTTCTGCCGGTTCTATAAGGGGGAAGCTTTTCAGGCTGGTCTCGCTCGTCACGAGCATTACCTCGCCGCACAAATGTCTTTGCGGCCCGGCATGAAGGTTCTTGACGTTGGCTGCGGCGTTGGTGGCCCAGCACGCGAGATTGCGCGCTTCGCGGACGTGTCCGTCGTCGGCGTCAACAACAACGATTTTCAGATCGGTCGTGCTAGGAGTTACACAAAAAAGGCAGGGCTCGAGGGTCAGGTCTCTTTCGCGAAAGGCGACTTCATGAAGCTCTCAGAACAGTTCGGCGAGAACTCTTTTGATGCCGgttagtaccaacattgattgaaaatgctgaaggactggcctgacaatgttatgtagTGTATGCCATCGAGGCTACGGTACATGCTCCCACCTGGGAGGGTGTATATGGCGAAATTATGAAGGTTCTGAAGCCCGGAGGAGTGgtatgtattttcggtctccatatcgtgattgcaactcacatttcttgtcagTTTGGTGTGTACGAATGGTGTATGACCGACGCGTGGGACCCATCCATTCCCTCACATCGCGACCTTGCGCATCGTATCGAGATTGGGAACGGCATCCCCGAGATGCGGCCTCTGCAAAAAGCACGCGATGCTATGGTCAACGTCGGCTTCAAGATCGAGCACGAGGAGGACCTCGCCGAGCGGCCAGACGACGTGCCTTGGTTCTACCCGCTCGAGGGTGACATCTTCAAGGCGCAGACGGCTTGGGACTACGTCACGGTTTGGCGCATGAGCTGGAGTGGCAAGCTCGTGACGCACTATGGGTTATGGTTCATCGAGCAGCTCGGTCTTGTGCCAAAAGGTACCTGGGATGTTGGTGAGACCCTCAAAATCGCTGGTGACTCTTTAGTTGAGGGCGGGAGGGCAAAGgtcggtttattatcacgtctcgcacagtctttcacagtcgcgctgatattgttcccgatacagCTTTTCACTCCGATGTATCTTGCCATTTGCAGGAAGCCTGTGAACTGA |
| *TgErg6 2* | scaffold9.g117 | ATGTCTTCTGTTGTAACTCAAACCGCGTCCGATGGCCGTGTAGGCACTCGTATCCAGAACTACACAAGTCATTGGCAAAAGGACTTGAGCAAGGACACTAAGGTCGACAACCGCGTTCAGAATTACACAGACGTCATTAACGgtatcgccagtcttcacatgtgagatcgcagttcttaccatgcgagcagGCTATTATGATGGCGCGACTGAGCTGTATGAATACGGGTGGGCGCAGTCTTTCCACTTCAGCCGCTTCTACAAAGGCGAGGGCTTCGCAGCTGCACTTGCTCGCCATGAACATTACCTTGCGGCGAGAATGTCGTTAAAGCCAGGAATGAAGGTCCTCGACGTCGGTTGCGGTGTGGGTGGCCCGGCGCGCGAGATCGCACAGTTTGCAGACGTAGAGATTGTAGGACTGAATAACAACGAGTTCCAGGTCGGGCGTGCAGCGAAGTACACGCACCAGGCCAGGCTGACGGGTCAAGTCTCGTTCGTGAAGGGCGATTTCATGAAGCTCGCAGAGCAGTTCGGCGAAAATTCATTTGATGCCGgttcgtcaaagactgtccatagtagaagaggtcagggtgttgacagtgccccgcagTCTATGCCATGGAGGCTACTGTGCATGCTCCTACCTGGGAGGGCGTGTATGGCGAAATCATGAAGGTTTTGAAACCCGGCGGAGTGgtatgcaggtcatgcccttgctgtcgctgcattgttgctcatttgctcgtccagTTTGGCGTGTACGAATGGTGCATGACTGACAGGTGGGAGCCCACGATACCCAAGCACAAAGAACTCGCGCACCGCATCGAGATTGGCAACGGTATTCCTGAAATGCGCCCGCTCAACAAGGCACGCAAGGCGCTGCTCTCGGTCGGCTTTACGATAGAGCACGAAGAAGACCTAGCAGAGAGGAACGACGAGGTTCCGTGGTATTATCCTTTGGAAGGCAACCTTTTCCAAGCGCAGACAGCCTGGGACTACTTCACAGTTTGGAGGATGAGCTGGAGTGGCAAGCTTGTGACCCACTATGGATTATGGCTTATCGAGCAACTAAGGTTGGTGCCTAAGGGGACATGGGAGGTCGGAGAGACGCTTAAGGTCGCTGGAGACGCTCTCGTGGAAGGGGGGAAGACTAAGgtgagtctggacgttgcaatgtataacaccggttgatactaaatcttcgcagCTCTTTACGCCAATGTATCTCGTTGTCTGTAAGAAACCTCTCGCATCCTGA |
| *TgErg2* | scaffold9.g270 | ATGACAACACCTAAAACTAAGACTGCCCCCGGCAAAGCGCCTCAGCCGGTGAACGCGACGTTGATAAAATGGGTCCTGCGGTCTGCGTGGCTGTTCTTATTTATCGCAGCGTGCCAGTGGCTAGATGTCATTAAGgtacatctactgtgtcccgtagtgttcagttaggctggcgtggtatattcactggcttgtatagGACCGATGGCATGTCTTCACACCAGAAGGCTTGCACGAGCTCGCACAGGCTGCAGTCGCCGCTGCACCCGAGCCCAGCGACATCAACTTCATGGTCTCGTACATCATTTCCAACCTCACCGAGACCTACCCGTCGACTACCATCGCGCTGAACTCCAATAGCTCCGAATGGGTGCTGAACAACGCAGGCGGGGCCATGGGCGCGATGTACGTTGTGCACGCGAGCGTCACAGAGTACCTCATCGTGTTCGGCACACCTCTTGGGACTGAGGGACACACGGGTCTGCACACTGCAGACGACTATTTCAACATCTTGGTGGGCGAGCAGTGGGCATTCGCGCCGCCTAAGCTGGAGATGGAGGTGTATAAGCCTGGAAGCGTGCACCATTTGCCACGCGGACATGTGAAGCAGTACAAAATGCATGAAGGCTGCTTTGCCCTGGAGTATGCCAGAGGTGGGTCTGCTCTACTTTCGGGTGTAGGTCTGGGCTCACTAGCTCTTGCAACCGCACAGGCTGGATTCCGCTGA |
| *TgErg3* | scaffold6.g118 | ATGGATCTCGTTCTCAGCATCTGTGACGACCTCTTACTAGACAAAGTCTGGGCGGTTTTGCTACCCGCATCTGCCTTCGCTTCATCTCCGAGTGCGAGCTTCATCCAGGCTGCTGTCAACTCTTCATCCTATGTCCCTATCGTAGCCTCCCAATCTAAATGGTCAAATCTAATCTCGTACATCCCGCATCCACCCTTGCCCATCGAGCAGCTCGCTTCACCCGCCTCCCCCTCGTCAAGTCTTGTTTCTGCATGGCCCCGAAATTATGTTCCACGGCAGGTTCTCTCACTCCTTGTTCTCACGCTCATCGGCATCCATTTCCTCTACTTTTCGTTCGCATGGCTATCATACAAATTTTTTTTCAATCACGAGATGATGAAGCATCCGCGGTTCCTGAAGAACCAGGTCAAGATGGAGATTCAAACCAGTTTGAAGGCATTCCCAGCCATGACCCTCCTTACTCTGCCGTGGTTCCAAGGAGAAGTTATGGGCTACTCGTTATTGTATGATAATGTCGCGGACTATGGATGGCCATATTTCTTCTTCTCAATAATCTGgtatgcatgaaactctttagcaagcgatctgtatctgagaatatttgcagGTTCCTCGTCTTCACCGACTTCGGGATCTATTGGGTCCACCGGTGGGAACATCACCCTATCTGCTATAAGTGGCTGCACAAACCTCACCACAAGTGGATTAgtatgcgcattacgttatgtcttacctacacttcgagctgacacacctccagTCCCTACTCCCTTTGCATCTCATGCGTTCCACCCGCTTGACGGCTACCTTCAGTCCGTCCCGTACCACCTTTTCATTTTCCTATTCCCTCTCCAGCGCATACTCTACCTGGGTCTCTTCGTGTTCGTCAACTTTTGGTCGATTTTGgtgagtgattttacgtcaactgtccgatcgcgattgttaagctttttgatctctcagATCCATGATTCGGACATGATCACCGGGCACCCGTTCGAGAAGGTGATCAACGGCCCTGCACACCACACGCTGCACCACTTGTACTTCACAGTGAACTATGGACAGgtcagtgaaatttccaacgactctgtcaccacatgatattcatatttgatccttgtctagTATTTCACATGGGCAGACCGGATGGGTAACTCGTACCGCCAGCCCAAGTCCGAGCTCGACCCTCTCCTGGAAGTGCATACCGCAGAGTCGAAGAAAGAAAAGGAAGCTCTAGGAAAGGAGCAATGA |
| *TgErg5* | scaffold1.g201 | ATGGCGTCCGCGCCCAGAGACCATTCCGTGTCCGCCTCCATGCCTTCCTACACCTATTCCCCCCTTCATGACATGCGCATCCCATCCTTCCTATCCTCTGCTTCCGGGTCTGCGACCTGGCTGTACACGACCGTCGCCATCATCCTCGCCCTCCTCGCCCTCGAGCAGTCCGTCTACAGGTACAAGAAGCGCCATCTCCCCGGGGACAACTGGACCATTCCCGTCATTGGCAAGTTCGCCGACTCTCTCAAGCCCAGTCTCGAGGGGTACATGAGGCAATGGGACTCGGGTGCCCTGAGCGTCGTCAGCGTTTTCAACATgtaagttgtcgcgctggtctctccttaatcggttgctcaggcgtgcaacattgatcgacagCTTCATCGTCATGGCCTCAACCAATGACCTTGCTCGCAAGATCCTCAACTCTCCAAGTCACGCCGAGCCGTGTCTCGTGCACTCTGCGAAGCAGATTCTGCGCCCTGACAATTGgtacgagactgctgtttatggaccgcgatcacagtgatgtctatatatttctttgcagGGTGTTCCTTTCAGGGAAGGTCCACGTCGAATACCGTCGGGTCTTGAATACACTCTTCACTCGGAAGGCTCTCGGgtacgtaacgggagccgaggcctgataacgccctgcttaaagctaatctctccacacttggccgttctagCGTGTACATCGGCATCCAAGACGCGATCACGCGCAAGCACTTCAGCAAGTGGCTCGCGGAGGCCGCGCAGGACCCGGCGCCGAAGCCGATCATGATGCTCGCGCGCGACCTCAACATGGACACGTCCCTCCGCGTCTTCTGCGGCCCGCACATCGGCGACGCGGCGGCGAGGGAGATCACGGACAAGTACTGGCTGATCACCGTCGCGCTCGAGCTCGTCAACTTCCCGCTCGCGCTCCCGGGCACGAAGGTGTACCGCGCGATCCAGGCGCGCAAGGCGGCGCTCGCGCACCTCGAGCGCGCGGCGTGCCTGAGCAAGCGCGCGATGGCCGCGGGCGCGGAGCCGGAGTGCATGCTCGACCAGTGGGTGAGCGACCTCGCGGACCCGGAGTACAAGGGCCGGCGCGACTTTAGCGACCTCGAGATGGCGATGGTGCTCTTCTCGTTCCTCTTCGCGAGCCAGGACGCGATGAGCAGCGGGCTCATCTACGGCCTGCAGCACCTCGCGGACCACCCCGCGGTGCTCGCGCGCGTGCGCGAGGAGCAGGAGCGCGTGCGTGGCGGGGAGTACGGGAAGGCGATCACGCTCGAGATGATGGACGCGATGCCGTACCTGCGCGCGGTGGTGAAGGAGAGCCTGCGGTTGAGGCCGCCGGTGACGATGGTGCCGTACAAGACGACGAAGGCGTTCCCGATCTCGGACGACTACACCGTGCCCGTGAACAGCATGATCATCCCGTCGTTCTACAACTCGCTGCACGACCCGGAGGTGTACCCCGACCCGGATGCGTTCCTGCCCGAGCGCTGGCTCGACCCCGAGAGCAGCGCGAACCAGAACCCGCGCAACTACCTCGTGTTTGGCAGCGGCCCGCACAAGTGCATCGGTCTGGAGTATGCGGTGATGAACATCGGATTGGTGCTTGCGGATGCTGCTGTTCTGCTGAACTGGGAGCATGTGCTGACGGAGAAGAGCAACAAGGTTGCgtgagtggtgtttctcgtcgccgaggatatcggtattgatgttttgtctgccccagTATCATTGCTACGCTCTTCCCCCAGGACGGCTGCTTGGTGAAGCTCAGCCCGCGCTGCTAG |
| *CoQ2* | scaffold3.g419 | ATGGCTTCCTATGCTCTGCAGACGCCATTCACGACCCCACTCACATATATTAGTCTGTTTGGTATCGGGGCTCTCGTCATGCGTGGTGCAGGCTGCACGATCAATGACATGTGGGATAGAAACTTGGACAAGGCTGTGGgtatgtggaccaacgacgtgtcctcgagctctactgactgtgtcgaagAGAGGACGAGGGAGAGACCATTAGCAAGGGGCGATATCTCTCGACCTCAAGCTGTTGGGTTTCTGGGTCTGCAGCTCACCGCTGGACTGGCGGTGTTGACACAGCTGAATTGGTACAGgtacggccctactacgcatacccgaatgctcagtataagtgatatgcgtagTATCCTATTGGGCGCGTCGTCCCTCTCACTCGTGACGATATATCCGTTTATGAAACGAGTCACTCACTGGCCGCAAGCCGTCCTTGgtgagtacacatcaccgcatagtccctgacctgactgtttgctgcagGACTCGCGTTCAATTGGGGAGCGCTTCTCGGGTGGTCCGCAGTTGCAGGCTCAGTGAATTGGAGCGTCGCTCTCCCGCTGTATGCTGGTGGAATCTGCTGGACACTCGTCTATGACAGCATTTATGCCCACCAAgtaagcgttgactcgatccccttccagtccatgtgccgatgcttctacagGACAAAACCGACGATATCCAAGTCGGAATCCGTTCGACAGCGCTGCTCTTTGGCGAGTACACGCGCCCAATTCTGAGTGCGCTTTCAGCCTCGTCTATGTCCCTAATCACCTATGCCGGGTACCTTAATGGACACGGGCCGCTATTCTACATGGGCACAGGTATCGCAGCCATTCAGCTCGCACGTGTCCTCGTGAGGACAGACTTCGATGACCGCCCGAGCTGCTGGAAGGGCTTTGTCGGATGTGGATGGTCTGGTTTCTGGGTGTGGATGGGTGCACTGGCCGATTATATCTTCATGATCTCTGGATTGGCTCTATAG |
| *ARO8* | scaffold2.g697 | ATGTCGAGCAATACTGTGGTAGACCTATCGCATCATCTCTCCTCGGAGGCTCGTGCGCGCAAGCCCAATCCTATGAAGGAGATATGGAAGCTCATTAAGCGAAAGCCCAACATGGTTTCACTGGCTAATGgtacgcataatatcacctgtcccattgtgcacaactcaacagcttgtccgcggttcagGGGATCCTCATTTCTCCCTATATCCCATCAAGAAGATGGAGTTTGAGGTTGCGTCCGTTGAGGAAGATGATCCGGTAACGTCCTGGCGTACGGCAGGCCCCAGTGCCCCTTCACAGACCCTCTCGTCGTCCAGAGACGAGCCATGCACCCTGTCCGTCAAAACGGCGCTACAATACAGCACCGGTGCCGGCCTTGTCGAAGCACAGCAGGCAGTCATGGAATTAACCAGGTTCTACCATTCGCCTCCTGATCACGTTTGCACCCTTTCACTAGGGAACGCAGATGGCGTCACCAAGTGTTTCCGGCTCTTGGGAGACTCTGGGGACTCTTTTCTGGCCGATGAATTTTCTTTTAGCTCTTTGACCAACGCGGGCCTGCCGCAAGGGATCAATTGGGTTCCTCTTAGGATCGACGACGGTGGTCTGGTACCGGAAGAGCTTGAGAGGCTTTTAGCGGGTTGGAATGAACAGCGAGGACGGAGACCGCATGTGCTATACACGGTTCCgtgagttttgatgtcatggtgaaatatctagaacgtttctgacccatcagacgctcagTTGCGGTCAAAATCCCACCGGATCGACATTGACCGTCGAACGGCGGAAAACAATATACGAGATCGCTCGCCGATACGACCTCATCATCATCGAAGACGgtaagtctaatttgccttatgtattccatatgttttacgcggcagctgcacagATCCGTACTACTTCCTTCAGTACGCCTCGCCAGGCTCGGGGCAGGCCGAAACCGAACGTACTCTCATGCCTTCCTTCCTCTCCATGGACGTTGACGGTCGTGTCATACGGGTAGATACgtgagccggcgtcttcccgtcgcattcaatccttcaacactaacgccgtgtttcctccagGTTCAGCAAAATTGTCGCGCCAGGCATGCGCCTGGGCTGGATCACGAGCAGCGCCATGTTCCACCAGCACCTCGTCTCCTACACCGACTCCTCCACCCAGCACCCGCACGGCTTCGGGCAGGTCTTCGTCACGGAGATGCTCGGCCCGCGGGGGTGGCAGATGCGCGGGTTCGATCGCTGGGTGCGCAGCCTGTGCGCGGAGTACCGCCGGCGGCGCGACTTCTTCCTGGGCCTGTTCGAGCGCGAGGTCGGCGCGACGGGGCTCGCGCGCGCGGCGTCGCCCGAGGCAGGCATGTTTGTCTGGATCAGGGTCAACATCGAGCGCCACCCGCGGTACCGCGACGATCTGCGGATAACGGGCTGCGACTCGCGGACACCCGCACGGACGAATGTCAGGCAGCTCATGGAGGAGCTGTTCGAGAGGTGTTTGGACGGCGGGGTGGTCGTCATGCCGGGGTCGGTCTTTGCTTTGAGGGCTGAACCGGGTTGGGACGATACGGAACACCCTATAGATGACgtgagtgcatagatctggaggcagtcctatctttctctgatagcttaggtgtcgtagCGCTTGAACTTCCTCCGTGCAACGTTTGCTGGAACGGAAGAGACGATGCAGGAAGGTTTATCTATTCTCGGGAAGGTCCTCAAGGAGTTCTTTGAGGACGAACAATAG |
| *wrbA* | scaffold10.g16 | ATGTCGTCCCCCAAGATTGCCATCGTCATCTACTCCCTGTACGGCCACATCGTCAAGAgtgcgttttgcgcgtcttaccatcacccccgctgacatctccgttgcagTGGCCGAGTCCGTGAAGAGCGGTGTTGAGGCTGCCGGCGGCAGGGTCACCATCTACCAgtatgtgctttcagccgttgctcctctacgcgtgcgctgaccggtgtggtgctcagGATCGCCGAGACGCTCCCCCAGGAGGTCCTCGCCAAGATGGGCGCGCCCCCCAAGCCCGCCTATCCCGTCCTCGAGCCCACGGACCTCCCCAAATTCGATGCCTTCATCTTCGGTATCCCGACTCGCTACGGCAACATGCCCGCGCAGTTCAAGgtgcgtctccctcccctgtgtaagtgaaatatagtcatgctgacacgccccctgcgcaactagACCTTCTGGGACGCGACCGGCGGGCTCTGGGCGCAGGGTTCGCTCGCAGGCAAGTACGCGAGCGTGTTCGTCTCGACGGGCACGCCCGGCGGAGGCCAGGAGATCACCGCGTTGTCGATCGTCTCGACGTTTGTGCACCACGGCATCAACTTTGTGCCCCTTGGCTACAGCAAGACGTTCGCGCAGCTCGCCAACCTCTCCGAGGTCCGCGGTGgtgagttcccgttcccgttgccttttgcctgcgttatgggcctctgcatacgcgcatatacgggctgtcttttcacctcgacgttggcgctgactcgttcttacagGCTCCCCTTGGGGTGCCGGCACCTACGCGGGCGCGGACGGCTCGCGCCAGCCCAGCGCGCTCGAGCTCGAGCTCGCGAGGCTGCAGGGCGAGTACTTCTACGGTGTCGTCTCCAAGGTCAAGTTCTAA |

TABLE S10 Expression matrix for all samples and all metabolites.

TABLE S11 RNA-Seq expression matrix for all samples and all genes.

References

1. Cheng M-J, Chan H-Y, Cheng Y-C, Wu M-D, Chen J-J, Chen Y-L, et al. A new pyrrole metabolite from the endophytic fungus of *Xylaria papulis*. Chemistry of Natural Compounds. 2015;51:515-8. <http://dx.doi.org/10.1007/s10600-015-1327-3>

2. Ghosh J, Sil PC. Arjunolic acid: a new multifunctional therapeutic promise of alternative medicine. Biochimie. 2013;95(6):1098-109. <http://dx.doi.org/10.1016/j.biochi.2013.01.016>

3. Akihisa T, Nakamura Y, Tagata M, Tokuda H, Yasukawa K, Uchiyama E, et al. Anti‐inflammatory and anti‐tumor‐promoting effects of triterpene acids and sterols from the fungus *Ganoderma lucidum*. Chemistry & biodiversity. 2007;4(2):224-31. <http://dx.doi.org/10.1002/cbdv.200790027>

4. TAKAHASHI A, KUSANO G, OHTA T, OHIZUMI Y, NOZOE S. Fasciculic acids A, B and C as calmodulin antagonists from the mushroom *Naematoloma fasciculare*. Chemical and pharmaceutical bulletin. 1989;37(12):3247-50. <http://dx.doi.org/10.1248/cpb.37.3247>

5. Duru ME, Çayan GT. Biologically active terpenoids from mushroom origin: A review. Records of Natural Products. 2015;9(4):456.

6. Woldegiorgis AZ, Abate D, Haki GD, Ziegler GR, Harvatine K. LC-MS/MS based metabolomics to identify biomarkers unique to *Laetiporus sulphureus*. Int J Nutr Food Sci. 2015;4(2):141-53. <http://dx.doi.org/10.11648/j.ijnfs.20150402.14>

7. Salari MH, Sohrabi N, Kadkhoda Z, Khalili MB. Antibacterial effects of enoxolone on periodontopathogenic and capnophilic bacteria isolated from specimens of periodontitis patients. Iranian Biomedical Journal. 2003;7(1):39-42.

8. Hong G. Enoxolone suppresses apoptosis in chondrocytes and progression of osteoarthritis via modulating the ERK1/2 signaling pathway. Archives of Medical Science: AMS. 2020;20(3):947. <http://dx.doi.org/10.5114/aoms.2020.93211>

9. Guo ZY, Wu X, Zhang SJ, Yang JH, Miao H, Zhao YY. *Poria cocos*: traditional uses, triterpenoid components and their renoprotective pharmacology. Acta Pharmacol Sin. 2024. <http://dx.doi.org/10.1038/s41401-024-01404-7>

10. Akihisa T, Uchiyama E, Kikuchi T, Tokuda H, Suzuki T, Kimura Y. Anti-tumor-promoting effects of 25-methoxyporicoic acid A and other triterpene acids from *Poria cocos*. Journal of natural products. 2009;72(10):1786-92. <http://dx.doi.org/10.1021/np9003239>

11. Liang C, Tian D, Liu Y, Li H, Zhu J, Li M, et al. Review of the molecular mechanisms of *Ganoderma lucidum* triterpenoids: Ganoderic acids A, C2, D, F, DM, X and Y. European Journal of Medicinal Chemistry. 2019;174:130-41. <http://dx.doi.org/10.1016/j.ejmech.2019.04.039>

12. Zheng C, Rangsinth P, Shiu PH, Wang W, Li R, Li J, et al. A review on the sources, structures, and pharmacological activities of lucidenic acids. Molecules. 2023;28(4):1756. <http://dx.doi.org/10.3390/molecules28041756>

13. Iwatsuki K, Akihisa T, Tokuda H, Ukiya M, Oshikubo M, Kimura Y, et al. Lucidenic acids P and Q, methyl lucidenate P, and other triterpenoids from the fungus *Ganoderma lucidum* and their inhibitory effects on Epstein− Barr virus activation. Journal of Natural Products. 2003;66(12):1582-5. <http://dx.doi.org/10.1021/np0302293>

14. Zhao X-R, Huo X-K, Dong P-P, Wang C, Huang S-S, Zhang B-J, et al. Inhibitory effects of highly oxygenated lanostane derivatives from the fungus *Ganoderma lucidum* on P-glycoprotein and α-glucosidase. Journal of Natural Products. 2015;78(8):1868-76. <http://dx.doi.org/10.1021/acs.jnatprod.5b00132>

15. Qiao Y, Zhang X-m, Qiu M-h. Two novel lanostane triterpenoids from *Ganoderma sinense*. Molecules. 2007;12(8):2038-46. <http://dx.doi.org/10.3390/12082038>

16. Isaka M, Chinthanom P, Kongthong S, Srichomthong K, Choeyklin R. Lanostane triterpenes from cultures of the Basidiomycete *Ganoderma orbiforme* BCC 22324. Phytochemistry. 2013;87:133-9. <http://dx.doi.org/10.1016/j.phytochem.2012.11.022>

17. Peng X, Liu J, Xia J, Wang C, Li X, Deng Y, et al. Lanostane triterpenoids from *Ganoderma hainanense* J. D. Zhao. Phytochemistry. 2015;114:137-45. <http://dx.doi.org/10.1016/j.phytochem.2014.10.009>

18. Tang Y, Zhao Z-Z, Feng T, Li Z-H, Chen H-P, Liu J-K. Triterpenes with unusual modifications from the fruiting bodies of the medicinal fungus *Irpex lacteus*. Phytochemistry. 2019;162:21-8. <http://dx.doi.org/10.1016/j.phytochem.2019.02.017>

19. Zamuner M, Cortez DA, Dias Filho BP, Lima MIS, Rodrigues-Filho E. Lanostane triterpenes from the fungus *Pisolithus tinctorius*. Journal of the Brazilian Chemical Society. 2005;16:863-7. <http://dx.doi.org/10.1590/S0103-50532005000500028>

20. Wu X, Yang J-S, Yan M. Four new triterpenes from fungus of *Fomes officinalis*. Chemical and Pharmaceutical Bulletin. 2009;57(2):195-7. <http://dx.doi.org/10.1248/cpb.57.195>

21. Peng X-R, Su H-G, Liu J-H, Huang Y-J, Yang X-Z, Li Z-R, et al. C30 and C31 triterpenoids and triterpene sugar esters with cytotoxic activities from edible mushroom *Fomitopsis pinicola* (sw. Ex Fr.) Krast. Journal of Agricultural and Food Chemistry. 2019;67(37):10330-41. <http://dx.doi.org/10.1021/acs.jafc.9b04530>
