# Supplementary figures and images for: Multi-omics analysis of Taiwanofungus gaoligongensis: effects of different cultivation methods on secondary metabolites
Source: Front Microbiol. 2025 Aug 1;16:1620693. doi: 10.3389/fmicb.2025.1620693 (PMC12355660; doi:10.3389/fmicb.2025.1620693)

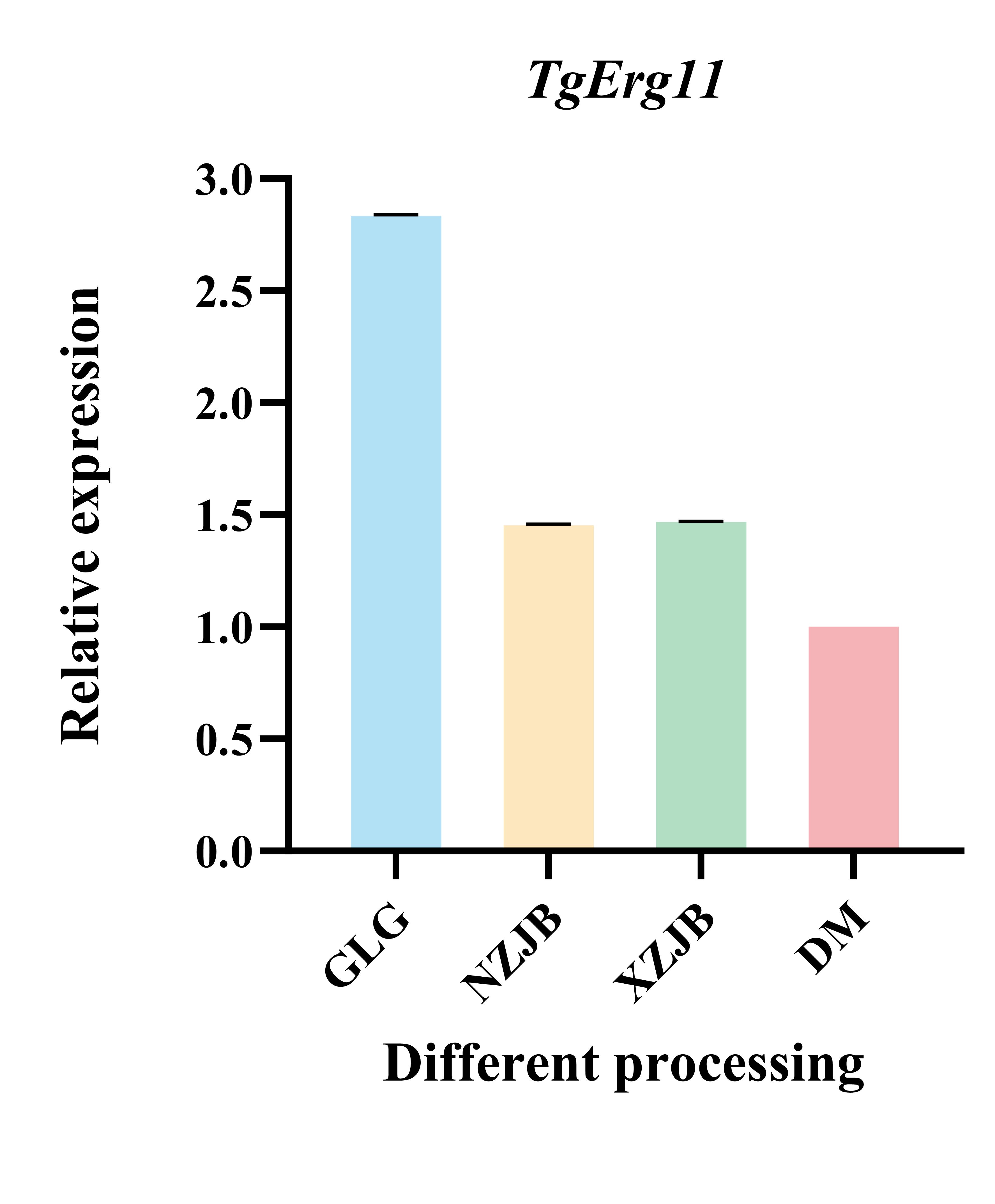

Supplement: Supplementary file 1 [file Supplementary_file_1.zip › Supplementary file 1/Data Sheet 1/PCR图/TgErg11.jpg]

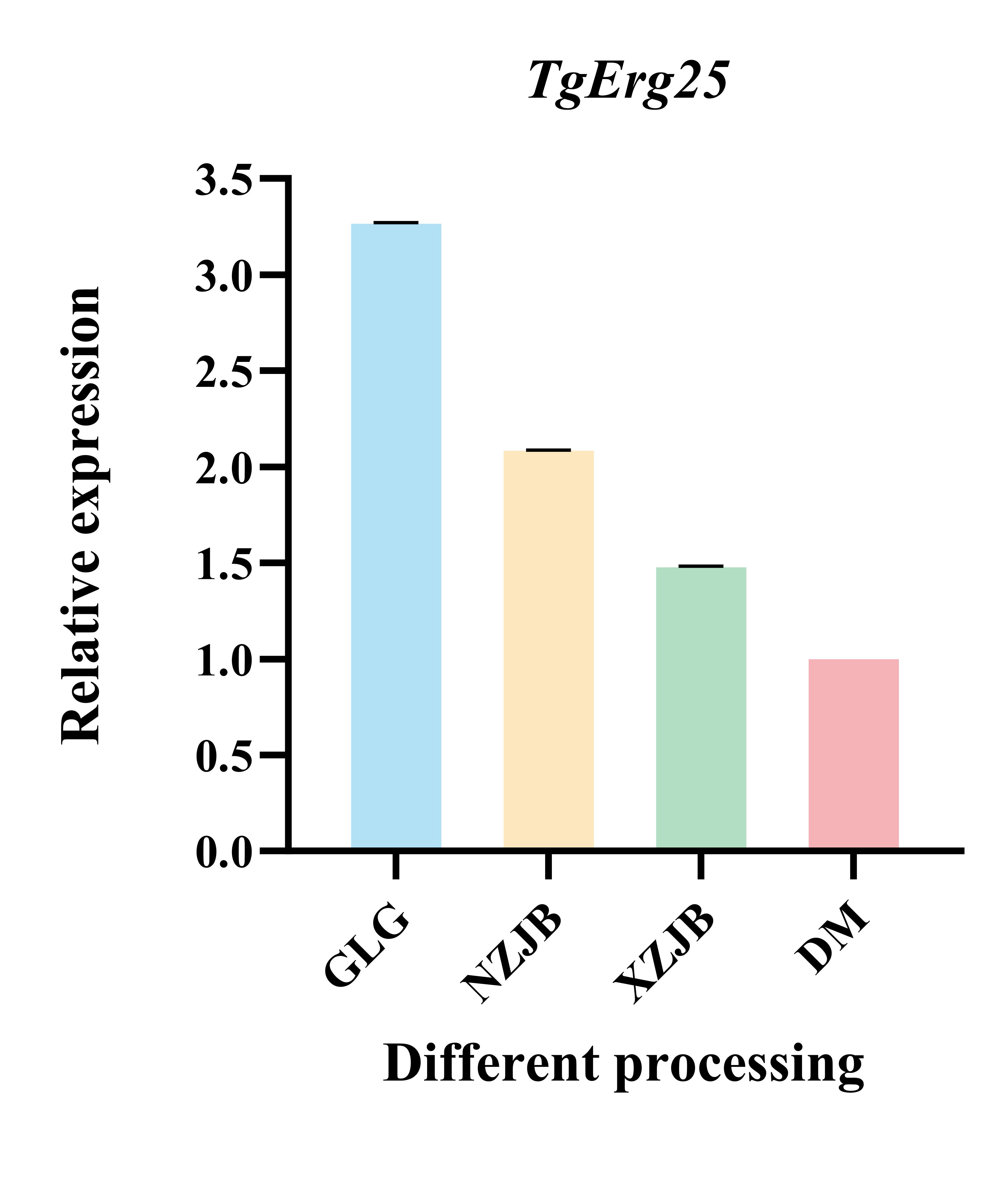

Supplement: Supplementary file 1 [file Supplementary_file_1.zip › Supplementary file 1/Data Sheet 1/PCR图/TgErg25.jpg]

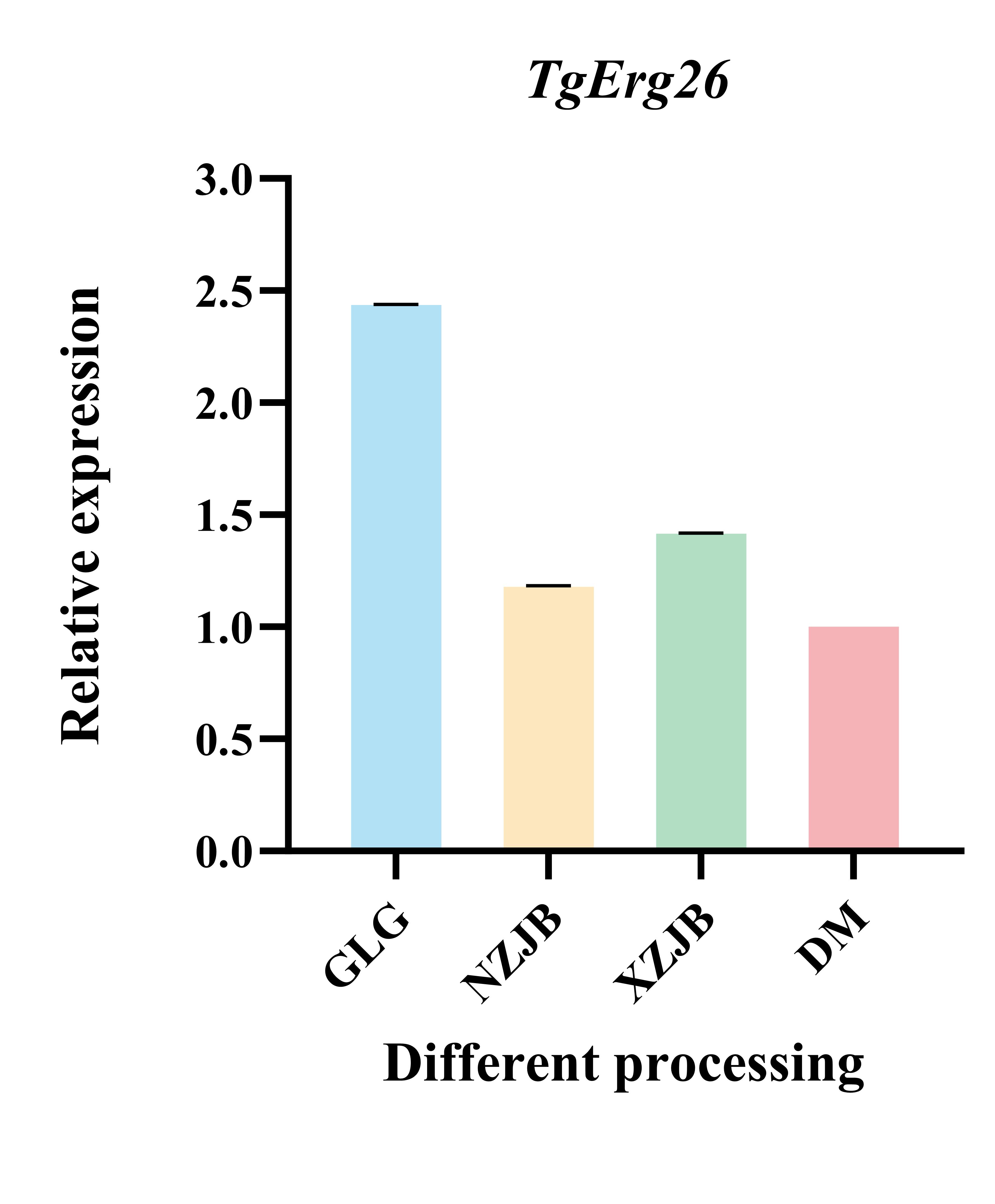

Supplement: Supplementary file 1 [file Supplementary_file_1.zip › Supplementary file 1/Data Sheet 1/PCR图/TgErg26.jpg]

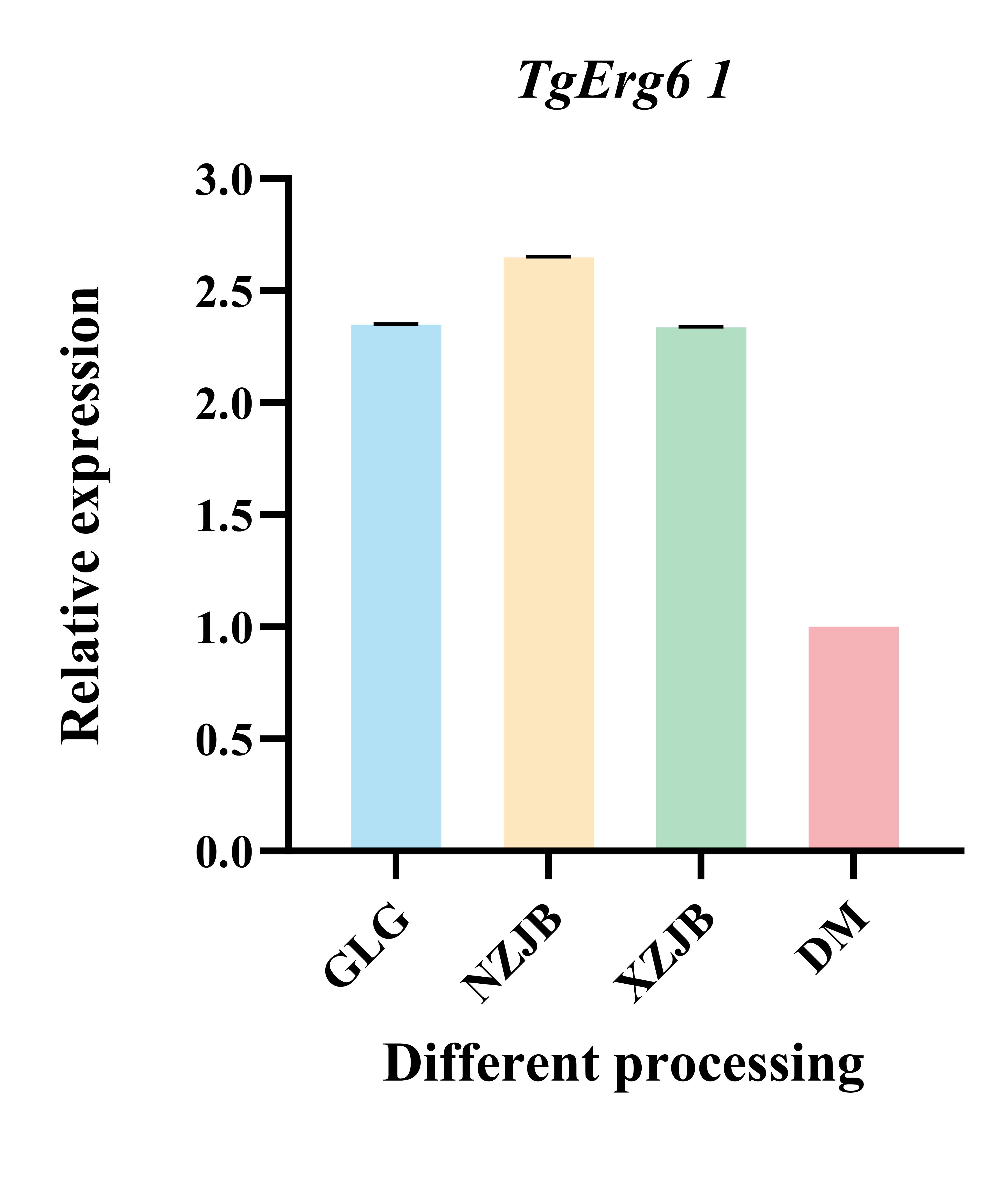

Supplement: Supplementary file 1 [file Supplementary_file_1.zip › Supplementary file 1/Data Sheet 1/PCR图/TgErg6 1.jpg]

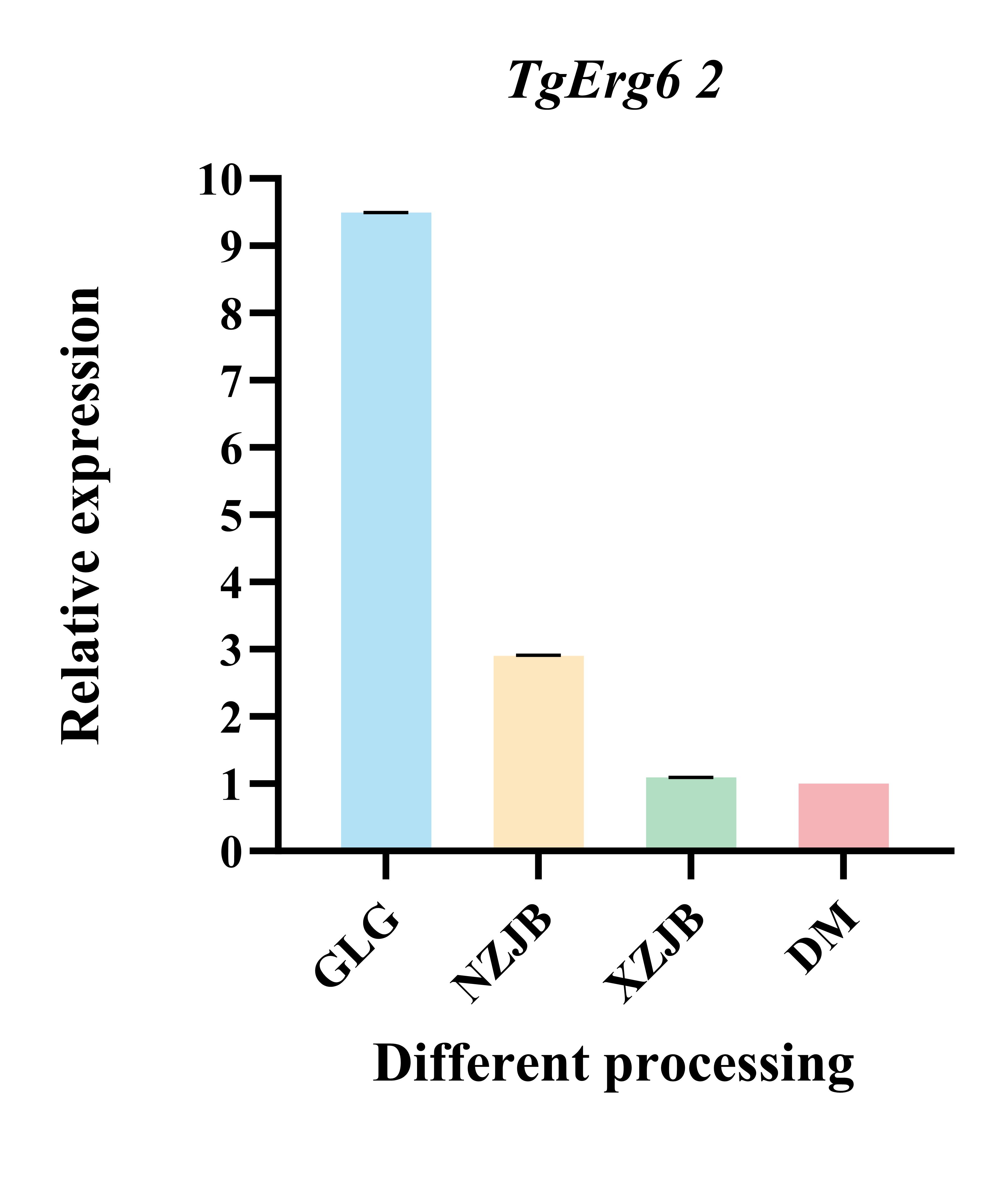

Supplement: Supplementary file 1 [file Supplementary_file_1.zip › Supplementary file 1/Data Sheet 1/PCR图/TgErg6 2.jpg]

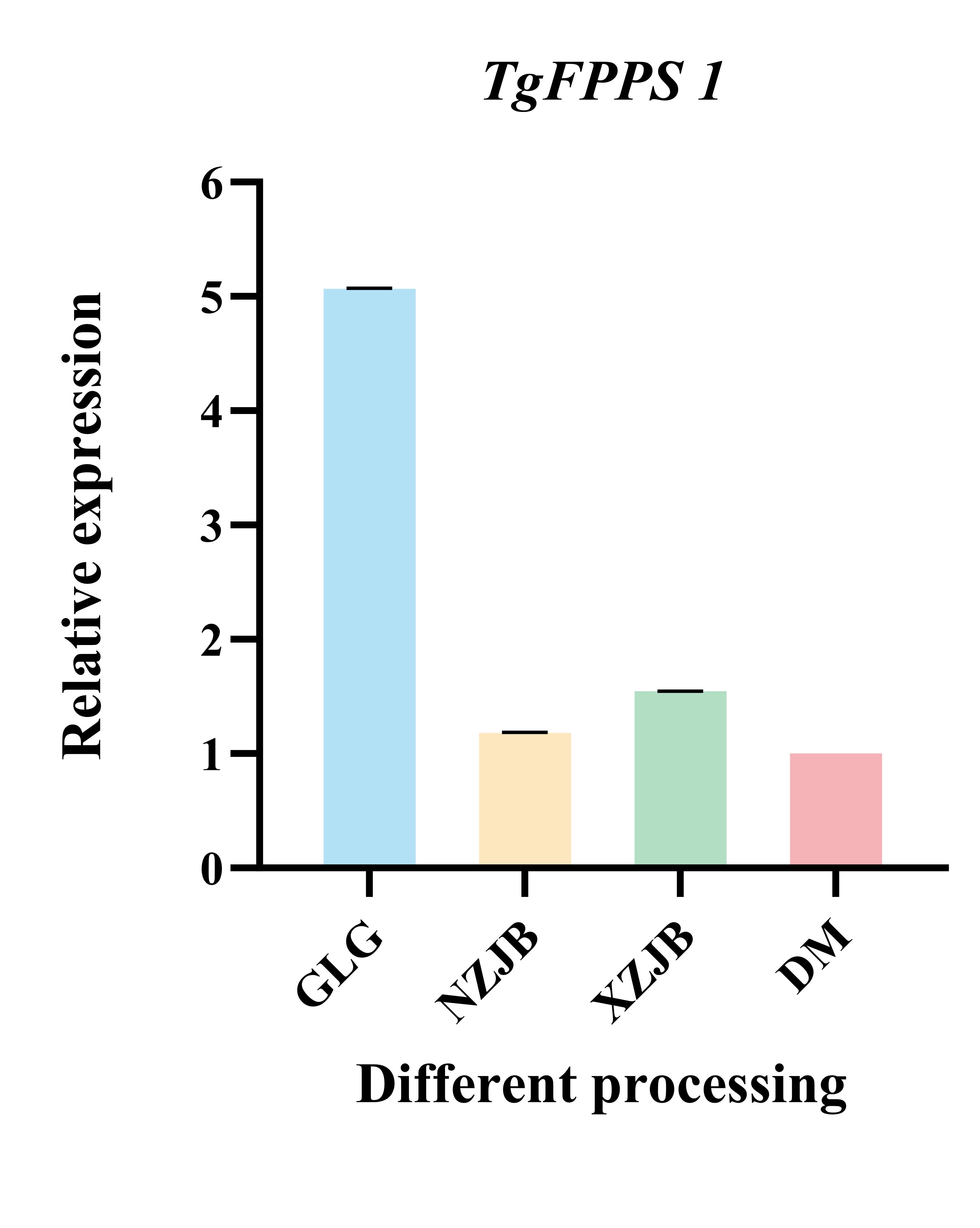

Supplement: Supplementary file 1 [file Supplementary_file_1.zip › Supplementary file 1/Data Sheet 1/PCR图/TgFPPS 1.jpg]

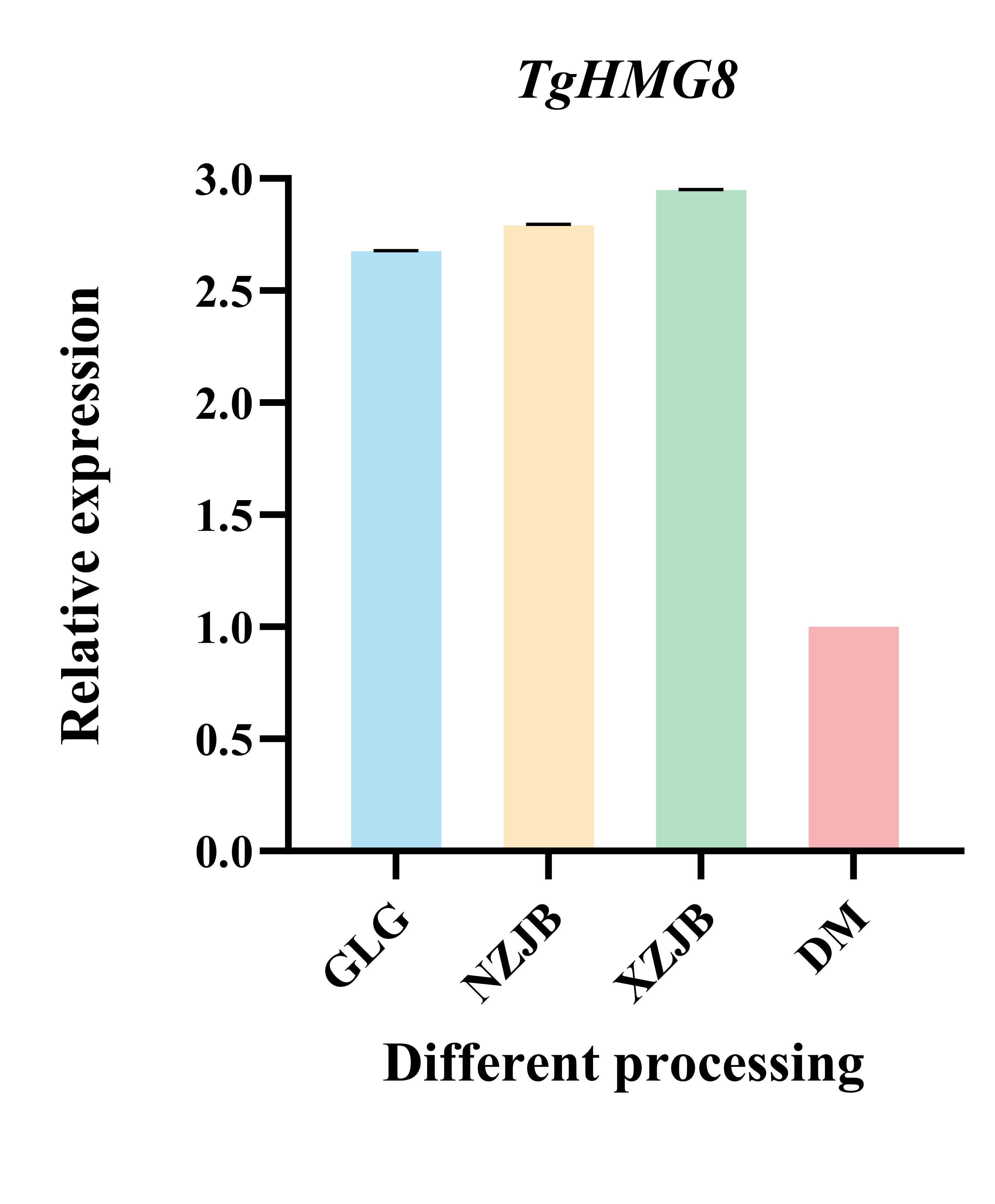

Supplement: Supplementary file 1 [file Supplementary_file_1.zip › Supplementary file 1/Data Sheet 1/PCR图/TgHMG8.jpg]

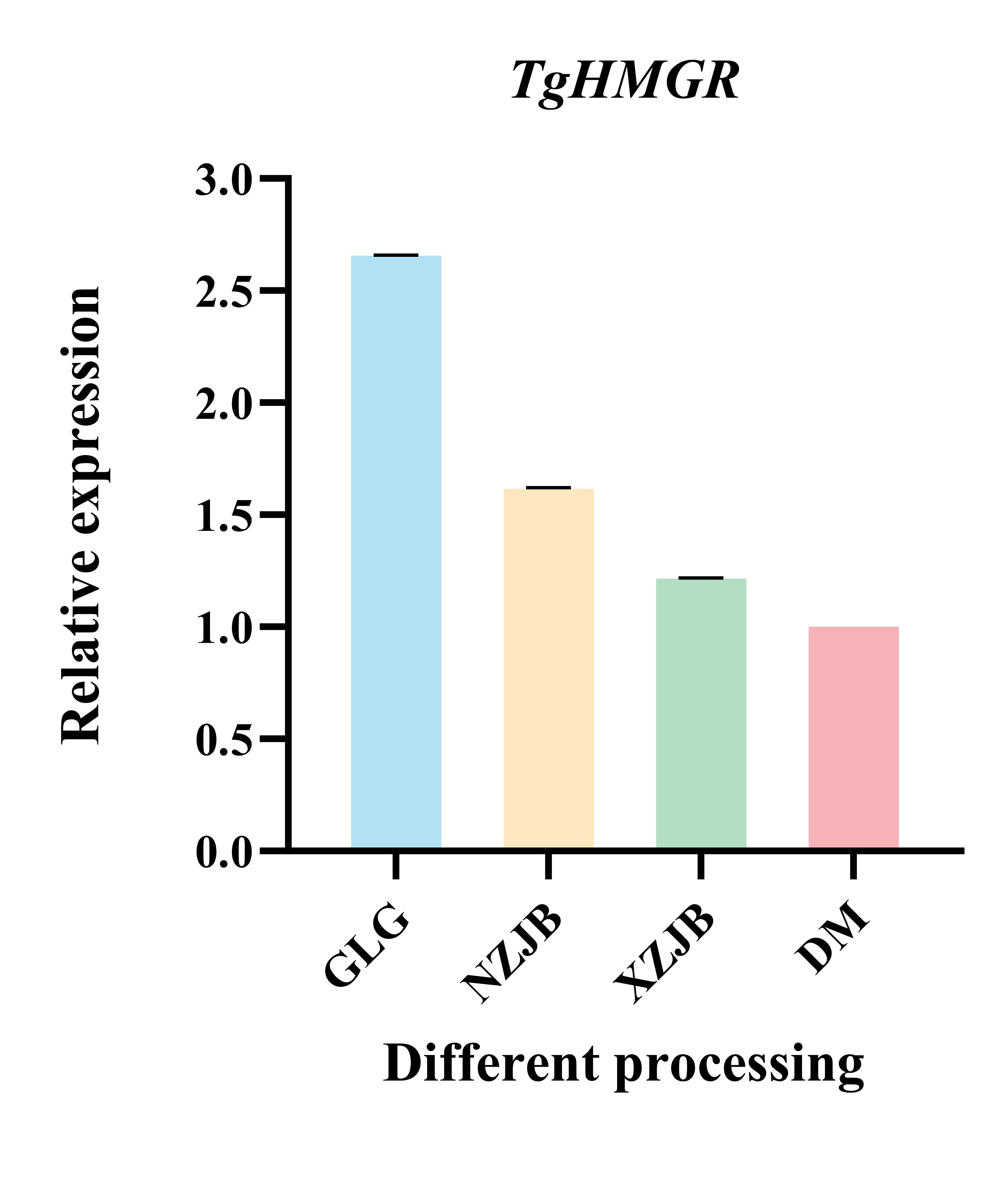

Supplement: Supplementary file 1 [file Supplementary_file_1.zip › Supplementary file 1/Data Sheet 1/PCR图/TgHMGR.jpg]

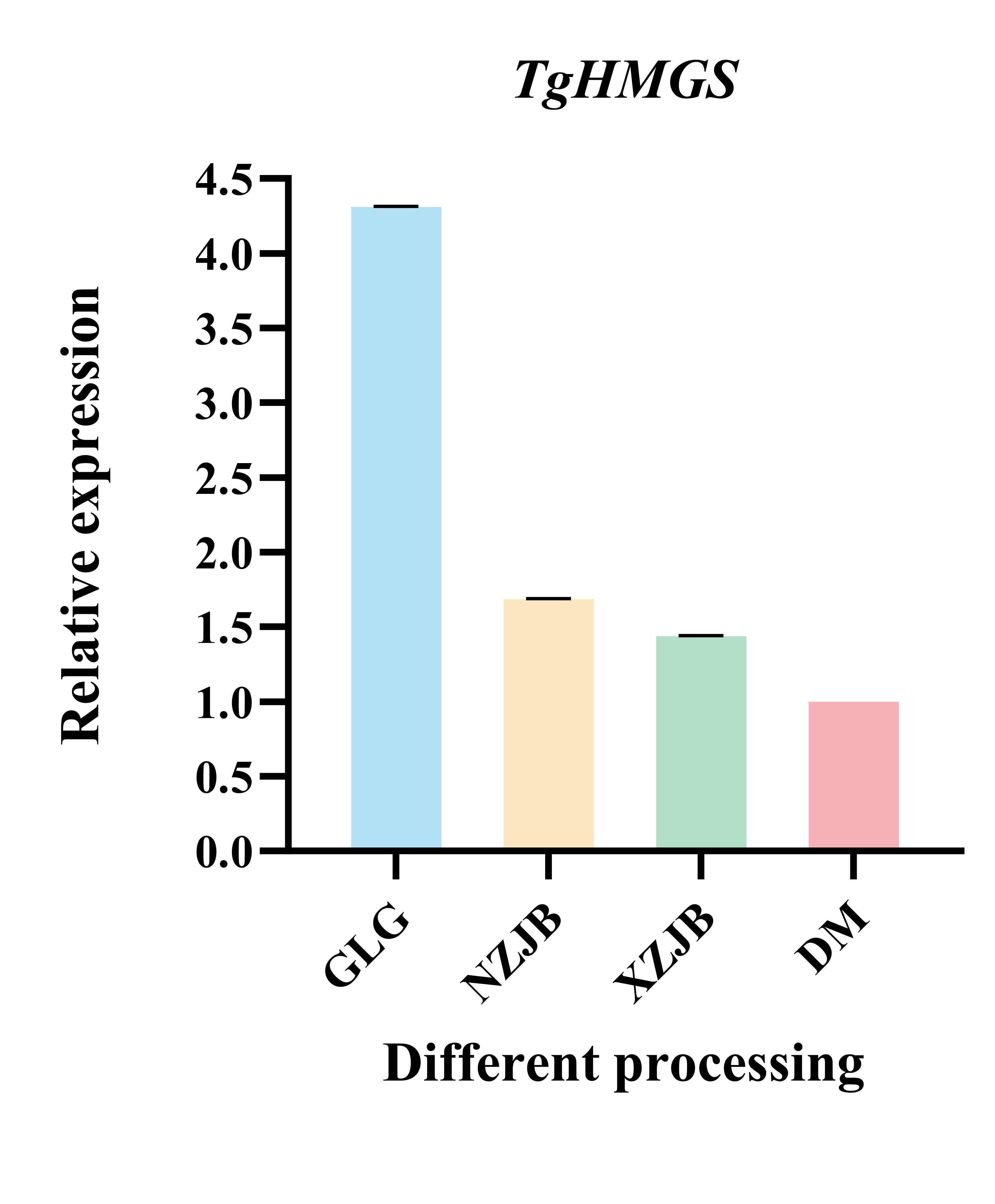

Supplement: Supplementary file 1 [file Supplementary_file_1.zip › Supplementary file 1/Data Sheet 1/PCR图/TgHMGS.jpg]

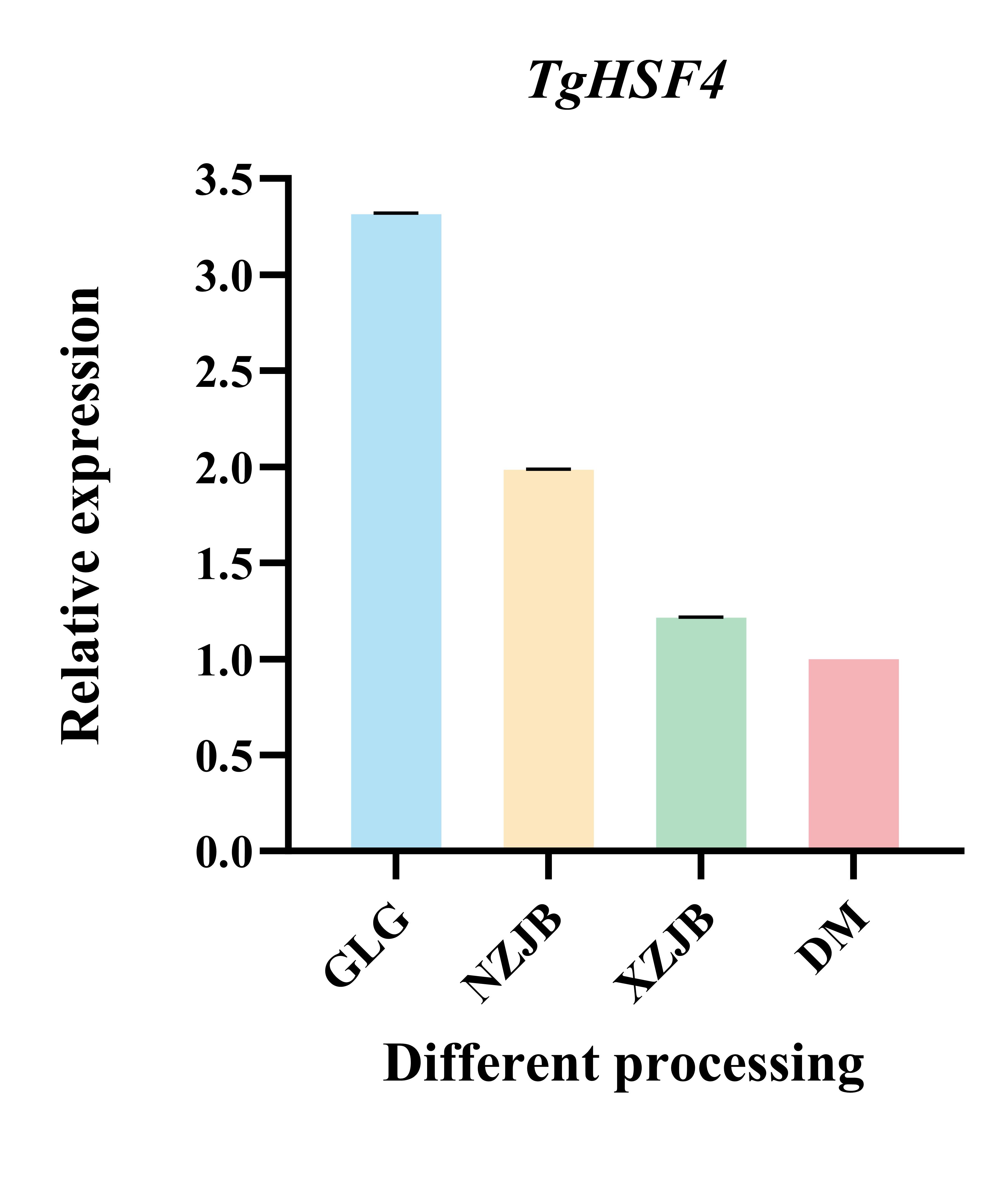

Supplement: Supplementary file 1 [file Supplementary_file_1.zip › Supplementary file 1/Data Sheet 1/PCR图/TgHSF4.jpg]

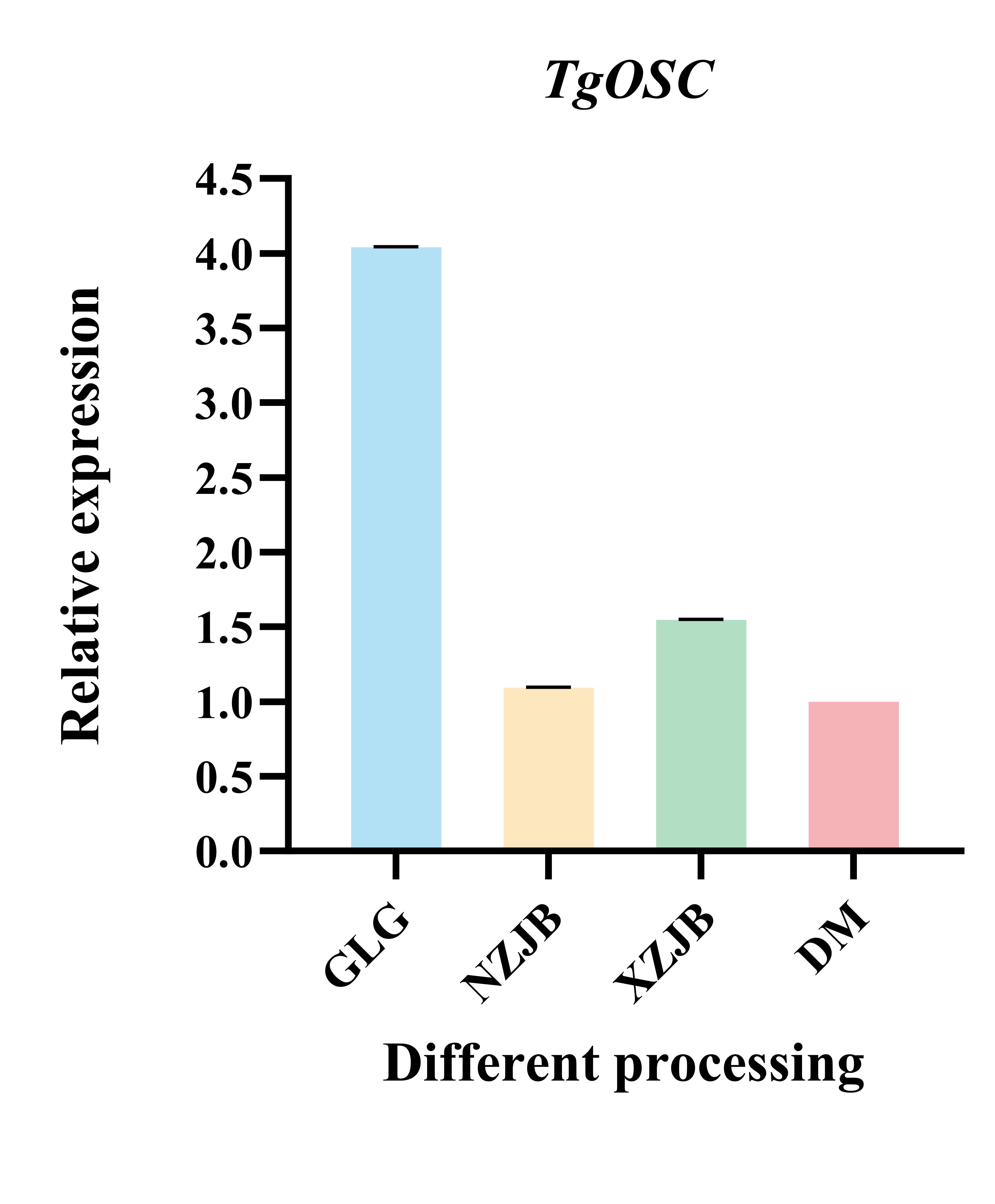

Supplement: Supplementary file 1 [file Supplementary_file_1.zip › Supplementary file 1/Data Sheet 1/PCR图/TgOSC.jpg]

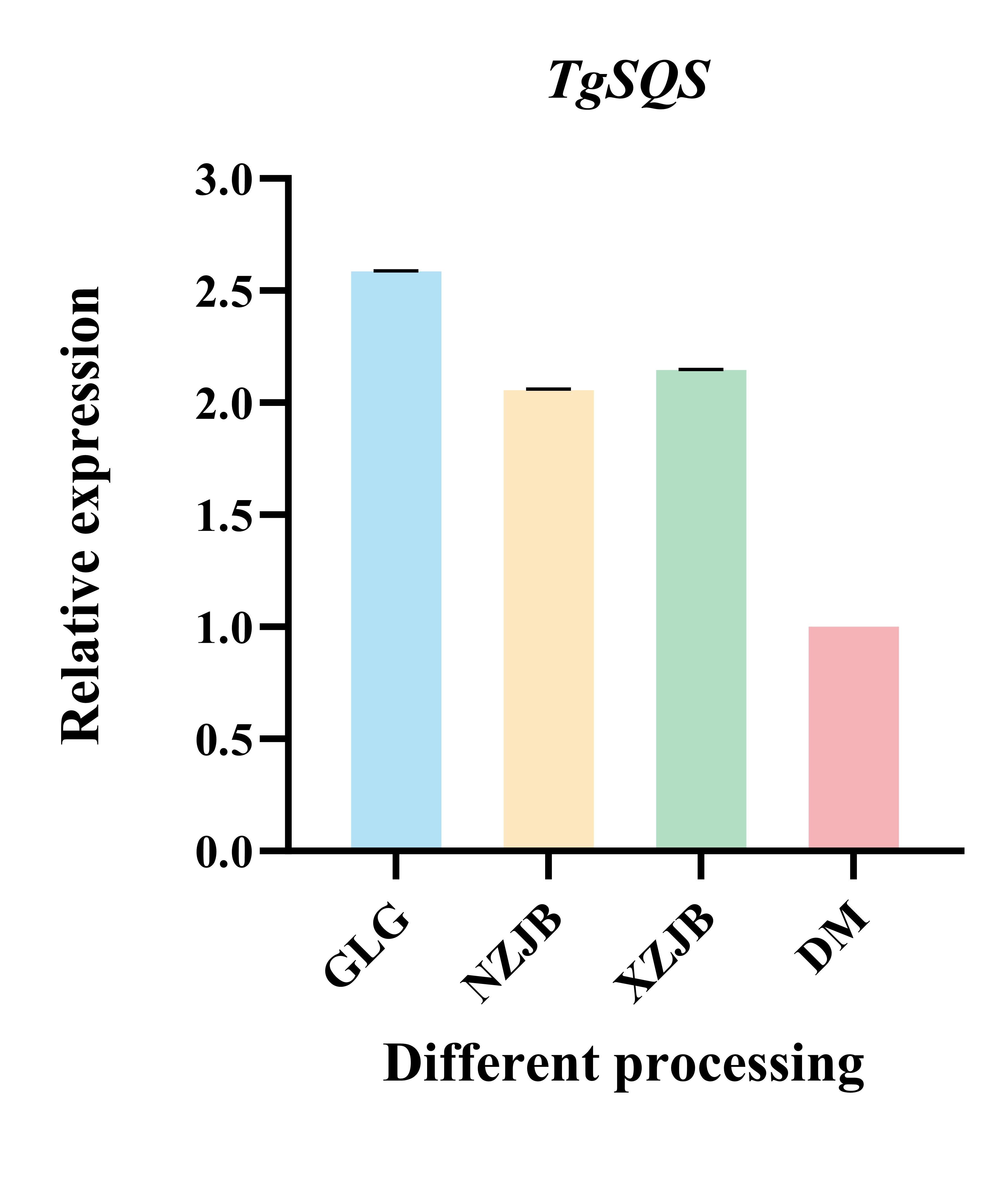

Supplement: Supplementary file 1 [file Supplementary_file_1.zip › Supplementary file 1/Data Sheet 1/PCR图/TgSQS.jpg]
